# A T follicular helper cell origin for T regulatory type 1 cells

**DOI:** 10.1038/s41423-023-00989-z

**Published:** 2023-03-27

**Authors:** Patricia Solé, Jun Yamanouchi, Josep Garnica, Muhammad Myn Uddin, Robert Clarke, Joel Moro, Nahir Garabatos, Shari Thiessen, Mireia Ortega, Santiswarup Singha, Debajyoti Mondal, César Fandos, Julio Saez-Rodriguez, Yang Yang, Pau Serra, Pere Santamaria

**Affiliations:** 1 Institut D’Investigacions Biomèdiques August Pi i Sunyer, Barcelona, Spain; 2 Department of Microbiology, Immunology and Infectious Diseases, Snyder Institute for Chronic Diseases, Cumming School of Medicine, University of Calgary, Calgary, AB Canada; 3 Institute of Computational Biomedicine, Faculty of Medicine, Heidelberg University, Heidelberg, Germany; 4 Department of Biochemistry and Molecular Biology, Cumming School of Medicine, University of Calgary, Calgary, AB Canada

**Keywords:** T-regulatory type 1 (TR1) cells, autoimmunity, T follicular helper (TFH) cells, transdifferentiation, nanomedicine, Autoimmunity, Target identification, Immunosuppression

## Abstract

Chronic antigenic stimulation can trigger the differentiation of antigen-experienced CD4^+^ T cells into T regulatory type 1 (TR1) cells, a subset of interleukin-10-producing Treg cells that do not express FOXP3. The identities of the progenitor(s) and transcriptional regulators of this T-cell subset remain unclear. Here, we show that the peptide-major histocompatibility complex class II (pMHCII) monospecific immunoregulatory T-cell pools that arise in vivo in different genetic backgrounds in response to pMHCII-coated nanoparticles (pMHCII-NPs) are invariably comprised of oligoclonal subpools of T follicular helper (TFH) and TR1 cells with a nearly identical clonotypic composition but different functional properties and transcription factor expression profiles. Pseudotime analyses of scRNAseq data and multidimensional mass cytometry revealed progressive downregulation and upregulation of TFH and TR1 markers, respectively. Furthermore, pMHCII-NPs trigger cognate TR1 cell formation in TFH cell-transfused immunodeficient hosts, and T-cell-specific deletion of *Bcl6* or *Irf4* blunts both the TFH expansion and TR1 formation induced by pMHCII-NPs. In contrast, deletion of *Prdm1* selectively abrogates the TFH-to-TR1 conversion. *Bcl6* and *Prdm1* are also necessary for anti-CD3 mAb-induced TR1 formation. Thus, TFH cells can differentiate into TR1 cells in vivo, and BLIMP1 is a gatekeeper of this cellular reprogramming event.

## Introduction

Interleukin-10 (IL-10)-producing regulatory T cells (Tregs) play a central role in the maintenance of normal immune homeostasis, and dysregulation of Treg cell development and/or function plays roles both in the development of autoimmune disease and in the progression of cancer.

In addition to the well-characterized FOXP3^+^ Treg cell subset, a number of other IL-10-producing but FOXP3^–^CD25^–^ and phenotypically heterogeneous subsets of Treg cells with low IL-4 secretion capacity (often referred to as T regulatory type-1, TR1 cells) have been described [1]. Examples include IL-10-producing FOXP3^–^ subsets characterized by the expression of Latent-associated peptide (LAP) and Lymphocyte-activation gene-3 (LAG-3) or the expression of C-C chemokine receptor type 5 (CCR5) and Programmed cell death protein-1 (PD-1) in the absence of CD25 (CD4^+^LAP^+^CD25^−^, CD4^+^LAG-3^+^CD25^−^ and CCR5^+^PD-1^+^CD25^−^, respectively). These cell types, which were identified as distinct based on incomplete marker sets [1], may correspond to cells of a single lineage. Although coexpression of CD49b and LAG-3 has been associated with IL-10 expression in both murine and human TR1-like cells in association with the expression of Inducible T-cell costimulator (ICOS) or of CCR5 and PD-1 [2–4], some of the above TR1-like subsets were LAG-3^low^ [4], and both markers lack the specificity and sensitivity required to address this conundrum. Other surface markers, such as Cytotoxic T-lymphocyte-associated antigen-4 (CTLA-4), T-cell immunoglobulin and mucin-domain containing-3 (TIM3) and T-cell immunoreceptor with Ig and ITIM domains (TIGIT), or transcription factors (TFs), such as T-BET (T-box expressed in T cells), AHR (Aryl hydrocarbon receptor) and NFIL3 (Nuclear factor, IL-3-regulated), have been found to be variably upregulated in some, albeit not all, TR1-like cells [5–7].

Given this complexity and the challenges associated with the lack of TR1 cell-specific markers enabling the isolation of this T-cell subset for detailed transcriptional and functional studies, whether the IL-10-expressing CD4^+^CD25^−^ T cells (‘TR1-like’) that arise in vivo in response to chronic antigenic stimulation (e.g., a chronic infection) or upon repeated administration of autoantigenic or allergenic ligands are alike or different and whether they can arise from various precursors such as TH1, TH2 and TH17 cells (reviewed in [8, 9]) are unclear. Furthermore, since other CD4^+^ T-cell subsets in addition to TR1 cells can produce IL-10, IL-10 production alone is not an indication of TR1 lineage identity. It is therefore possible that some of the phenotypes that have been ascribed to IL-10-producing FOXP3^−^ T cells correspond to non-TR1 cell types or to cell types at different stages of TR1 cell differentiation along a continuum, rather than to a bona fide subset of terminally differentiated TR1 cells with distinct phenotypic and/or functional properties and either stable or plastic gene expression programs. Unfortunately, the phenotypic correlates that help define true TR1-likeness and the transcriptional regulators that are responsible for the development of TR1 cells in vivo remain incompletely defined.

Systemic delivery of nanoparticles (NPs) coated with monospecific disease-relevant peptide-major histocompatibility complex class II (pMHCII) molecules [10] has been found to resolve inflammation in various organ-specific autoimmune disease models in a disease-specific manner without impairing normal immunity [11–13]. pMHCII-NP therapy functions by systemically reprogramming cognate antigen-experienced CD4^+^ T cells (excluding a role for naïve T cells) of unknown identity into expanded pools of monospecific TR1-like cells. These events result from the sustained assembly of large TCR microclusters on cognate effector/memory T cells, leading to rapid, robust and prolonged TCR signaling and the upregulation of known TR1-like cell markers such as c-MAF (musculoaponeurotic fibrosarcoma), IL-10, IL-21, LAG-3, ICOS and PD-1 in an antigen-presenting cell (APC)- and IL-27-independent manner [10, 11]. This ability of pMHCII-based nanomedicines to trigger the formation and systemic expansion of single-antigen-specific TR1-like cells expressing markers previously ascribed to different subsets of TR1-like cells affords a unique opportunity to unravel the gene expression landscape of these cells, provides insights into their developmental biology and allows the identification of stable gene expression signatures.

TFH cells arise from naïve CD4^+^ T cells in response to antigenic stimulation and, unlike other T-helper cell subsets, are programmed to migrate into the B-cell zones of secondary lymphoid tissues to promote the production of somatically hypermutated, high-affinity, class-switched antibodies (the germinal center (GC) reaction). Whereas BCL6 is a master regulator of TFH cell formation [14–16] and represses BLIMP1 expression [17], BLIMP1 upregulation, at least in B cells, silences the BCL6-induced transcriptional program [18]. T follicular regulatory (TFR) cells are negative regulators of the GC reaction that arise from natural FOXP3^+^ Treg cell precursors in a BCL6- and FOXP3-dependent manner [19]. Here, we show that TFH cells can differentiate into TR1 cells in vivo and identify BLIMP1 as a master regulator of this cell reprogramming event, exposing the TFH-TR1 axis as a potential new target for therapeutic intervention in infections, autoimmune conditions and cancer.

## Materials and methods

### Mice

NOD/ShiLtJ mice were obtained from the Jackson Laboratory (Bar Harbor, ME, USA). NOD.*Cd4-Cre* and NOD.*Flpe* mice were produced by backcrossing the *Cd4-Cre* and *Flpe* transgenes from B6.*Cd4-Cre* mice (Tg(Cd4-cre)1Cwi) and B6.*Flpe* mice (B6;SJL-Tg(ACTFLPe)9205Dym/J), respectively, onto the NOD.Lt background for at least 10 generations. NOD.*Bcl6*^*loxP/loxP*^, NOD.*Prdm1*^*loxP/loxP*^, NOD.*Irf4*^*loxP/loxP*^ and NOD.*Tbx21*^*loxP/loxP*^ mice were produced by backcrossing the *Bcl6*^*loxP*^, *Prdm1*^*loxP*^, *Irf4*^*loxP*^ and *Tbx21*^*loxP*^ genes from B6.*Bcl6*^*loxP/loxP*^ (B6.129S(FVB)-*Bcl6*^*tm1.1Dent*^/J), *Prdm1*^*loxP/loxP*^ (B6.129-*Prdm*1^tm1Clme^/J), B6.*Irf4*^*loxP/loxP*^ (B6.129S1-*Irf4*^*tm1Rdf*^/J), and B6.*Tbx21*^*loxP/loxP*^ (B6.129-*Tbx21*^tm2Srnr^/J) mice, respectively, onto NOD/ShiLtJ or NOD.*Cd4-Cre* background for at least five generations, followed by intercrossing. NOD.*Il10*^*tm1Flv*^ (Tiger) mice were obtained by backcrossing the *Il10*^*tm1Flv*^ allele from B6.*Il10*^*tm1Flv*^ mice (Jackson Lab) onto the NOD/ShiLtJ background for 10 generations [11]. NOD.*Il10-eGFP*.*Cd4-Cre*.*Bcl6*^*loxP/loxP*^ mice were produced by introgressing the *Il10*^*tm1Flv*^ allele from NOD.*Il10*^*tm1Flv*^ mice into NOD.*Cd4-Cre*.*Bcl6*^*loxP/loxP*^ mice.

B6 mice carrying an *Il10*^*loxP*^ allele were generated using the targeted embryonic stem (ES) cell clone EPD0158-4-D-06 from the EuComm consortium (knockout-first allele with conditional potential). The clone, which had chromosomal euploidy, was used to generate chimeric mice by microinjecting the ES cells into blastocysts obtained from the mating of C57BL/6-BrdCrHsd-Tyrc females with B6(cg)-Tyr<c2J > /J males (at the University of Michigan transgenic facility). Mice with high percentages of chimerism were bred for germline transmission, and mice were screened via PCR for the presence of the 5′ and 3′ Loxp sites. The primer sequences used to amplify the targeted alleles were as follows: Il10loxp int1-2 (5′) sense, cttcgtatagcatacattatacg and antisense, cagtatgttgtccagctggtc; Il10loxp int3-4 (3′) sense, gagaagctgaagaccctcag and antisense, cgtataatgtatgctatacgaag). The primers used to amplify the wild-type *Il10* allele were as follows: Il10 wt 5′ sense, cagtatgttgtccagctggtc and antisense, gaaaagctaactaggaggtga; Il10 wt 3′ sense, gagaagctgaagaccctcag and antisense, ctgtcagcccagctctgtgc). B6 mice carrying one copy of the *Il10*^null^ allele and one copy of the above *Il10*^loxP^ allele as well as a Tbx21-Cre transgene were generated by breeding mice harboring the various genes from the corresponding B6 stocks (B6.129P2-*Il10*^*tm1Cgn*^/J, B6.*Il10*^*loxP/+*^ and B6;CBA-Tg(Tbx21-cre)1Dlc/J, respectively).

NOD mice carrying two copies of a conditional (loxP-flanked) *Il2* allele were produced in-house. A NOD BAC containing *Il2* was targeted for the insertion of loxP sites flanking exons 3–4 and a downstream FRT-flanked inverted neomycin (neo)-encoding cassette. A ~15 kb fragment, excised by gap repair, was transfected into 129 ES cells (identical by descent at the *Il2* locus to NOD mice [20]), which were used to produce germline-competent mice. The neo-deleted alleles (upon crossing with NOD.*Flpe* mice) were backcrossed onto the NOD background for >10 generations, and the obtained mice were then intercrossed with NOD.*Cd4-Cre* mice to produce NOD.*Cd4-Cre*.*Il2*^*loxP/loxP*^ mice and NOD.*Il2*^*loxP/loxP*^ mice (Supplementary Fig. 10).

The experiments described herein were approved by the University of Calgary and Universitat de Barcelona Animal Care Committees.

### pMHC production

Recombinant pMHC class II monomers were produced in CHO-S cells transduced with lentiviruses encoding peptide-MHCα and MHCβ chains and IRES-CFP and IRES-eGFP cassettes separately. To express the various pMHCs, transduced CHO cells were grown in 2 L baffled flasks (Nalgene, ThermoFisher Scientific, Waltham, MA, USA) at 125 rpm with 5% CO_2_ at 37 °C. The basal medium was Power-CHO-2 (Lonza, Basel, Switzerland) supplemented with 8 mM glutamine (Cultek, Madrid, Spain) and gentamicin sulfate (0.25 mg/mL) (Lonza). The cultures were started in a volume of 400 mL of basal medium at a cell density of 350,000–400,000 cells/mL and were supplemented with Cell Boost 7a (HyClone, 3% v/v) and Cell Boost 7b (HyClone, GE Healthcare, Chicago, IL, USA; 0.3% v/v) on Days 0, 3, 4, 5, 6, 8, 9 and 10. A temperature shift to 34 °C was performed when the cell density reached 5–7 × 10^6^ cells/mL. Additional glutamine was added on Day 7 to 2 mM. Glucose was added to 4.5 g/L when the glucose level dropped below 3.5 g/L. Cells were harvested on Day 14 or when the viability fell below 60%. The secreted proteins were purified by sequential affinity chromatography on nickel and Strep-Tactin columns and were either used for NP coating or biotinylated in vitro to produce pMHCII tetramers.

### pMHCII tetramers

Phycoerythrin (PE)- or APC-conjugated tetramers were prepared using biotinylated pMHCII monomers and used to stain peripheral T cells. Briefly, pMHCII monomers were subjected to biotinylation using biotin ligase (Avidity, Aurora, CO, USA) following the supplier’s protocols and were then subjected to ion exchange chromatography using an AKTA FPLC system (GE Healthcare, Chicago, IL, USA). The final product was verified by denaturing SDS‒PAGE. Tetramers were generated by adding PE-conjugated streptavidin (Rockland Immunochemicals, Limerick, PA, USA) at a 4:1 molar ratio.

### Flow cytometry

To stain suspensions of mononuclear cells isolated from mice, splenic CD4^+^ T cells were incubated with avidin for 15 min at room temperature (to block biotin binding sites on the cells), stained with tetramer (5 µg/mL) in FACS buffer (0.05% sodium azide and 1% FBS in PBS) for 30 min at 4 °C, washed, and incubated with FITC-conjugated anti-CD4 (5 µg/mL) and PerCP-conjugated anti-B220 (2 µg/mL; as a ‘dump’ channel) antibodies for 30 min at 4 °C in the presence of an anti-CD16/CD32 mAb (2.4G2; BD Pharmingen, BD Biosciences, San Diego, CA or Biolegend, San Diego, CA, USA) to block FcRs. Cells were washed, fixed with 1% paraformaldehyde (PFA) in PBS and analyzed with FACSAria or BD LSRII flow cytometers. Analysis was performed using FlowJo software (FlowJo, BD Biosciences, San Diego, CA, USA).

FITC-, PerCP-, APC-, PerCPCy.5.5- or BV421-conjugated mAbs against mouse CD4 (RM4-5 or GK1.5), B220 (RA3-6B2), CD138 (281-2), TACI/CD267 (8F10), CXCR5 (2G8), PD-1 (CD279, J43), GATA3 (L50-823), RORgt (Q31-371), CD25 (PC61), CD62L (MEL-1), and CD44 (IM7) and streptavidin-APC as well as isotype controls were purchased from BD Biosciences (San Diego, CA, USA). The PB-conjugated anti-CD4 (GK1.5) antibody was purchased from BioLegend. Abs against murine LAG-3 (C9B7 W), CD127 (A7R34), c-MAF (sym0F1), and FOXP3 (FJK-16s) were obtained from eBioscience (ThermoFisher Scientific, Waltham, MA, USA). Abs against T-BET (4B10), ICOS (CD278, C398.4A), and APC (APC003) were obtained from BioLegend (San Diego, CA). CXCR5 and LAG3 were stained first with APC-conjugated Abs and then with biotin-conjugated anti-APC and streptavidin-APC.

### Mass cytometry

Splenic CD4^+^ T cells (5 × 10^6^) isolated from BDC2.5mi-IA^g7^-NP-treated and DNP-KLH-treated NOD mice (*n* = 4 samples each) were stimulated with PMA (50 ng/mL) and ionomycin (500 ng/mL) in lymphocyte complete medium (LCM) for 4 h in the presence of GolgiStop and GolgiPlug (BD Biosciences, 1:1 v:v, using half the recommended amount for each). After a 10 min incubation with an anti-CD16/32 antibody (Biolegend, 5 µg/mL), the cells were incubated with BDC2.5 mi/IA^g7^-tetramer-PE (5 µg/mL) for 40 min at room temperature. Anti-CXCR5-biotin was added during the last 20 min of the tetramer staining step (5 µg/mL, BD Biosciences). Surface markers (shown in purple in Supplementary Table 4) were stained for 30 min at 37 °C. After viability staining with cisplatin (Fluidigm, San Francisco, CA), cells were fixed with a 1:1 mixture of FOXP3-Fixation/Permeabilization Solution (ThermoFisher) and Cytofix/Cytoperm Solution (BD Biosciences) for 30 min on ice. Cytokines and TFs (shown in green in Supplementary Table 4) were stained for 30 min at 4 °C. After staining with Intercalator-Ir (Fluidigm) overnight at 4 °C, cells were acquired with a Helios Mass Cytometer (Fluidigm). FCS files were analyzed using FlowJo to select single viable Tet^+^ (CD4^+^ tetramer^+^) cells and TFH (CD4^+^CXCR5^hi^PD-1^hi^) or Tconv (CD4^+^CXCR5^−^PD-1^–^) cells and were then further analyzed with viSNE using Cytobank (Cytobank, Santa Clara, CA). In anti-CD3-treated mice, an anti-CD49b-PE antibody (2 µg/mL, BD Biosciences) was used instead of tetramer-PE, and viSNE analysis was performed on single viable CD4^+^IL-10^+^ cells.

### Nanoparticle synthesis

Maleimide-functionalized, pegylated iron oxide NPs (PFM series) were produced via a single-step thermal decomposition reaction in the absence of surfactants as described recently [10]. Briefly, 3 g of maleimide-PEG (2 kDa MW, Jenkem Tech USA) was melted in a 50 mL round-bottom flask at 100 °C and then mixed with 7 mL of benzyl ether and 2 mmol of Fe(acac)_3_. The reaction was stirred for 1 h and heated to 260 °C under reflux for 2 h. The mixture was cooled to room temperature and mixed with 30 mL water. Insoluble materials were removed by centrifugation at 2000 × *g* for 30 min. The NPs were purified using magnetic (MACS) columns (Miltenyi Biotec, Auburn, CA, USA) and stored in water at room temperature or 4 °C. The concentration of iron was determined spectrophotometrically at 410 nm in 2 N hydrochloric acid (HCl).

### pMHC conjugation to NPs

pMHC conjugation to maleimide-functionalized NPs (PFM) was performed via the free C-terminal Cys engineered into the MHCα chain/knob. Briefly, pMHCs were mixed with NPs in 40 mM phosphate buffer (pH 6.0) containing 2 mM ethylenediaminetetraacetic acid (EDTA) and 150 mM NaCl and incubated overnight at room temperature. pMHC-conjugated NPs were purified by magnetic separation and concentrated by ultrafiltration through Amicon Ultra-15 centrifugal filter units (100 kDa cutoff) (Merck KGaA, Darmstadt, Germany) and stored in PBS.

### NP characterization

The size and dispersion of unconjugated and pMHC-conjugated NPs were assessed via transmission electron microscopy (TEM, Hitachi H7650, Hitachi, Chiyoda, Tokyo, Japan) and dynamic light scattering (DLS, Zetasizer, Malvern Panalytical, Spectris, Egham, UK). Pegylated and pMHC-NPs were analyzed via 0.8% agarose gel electrophoresis and native and denaturing 10% SDS‒PAGE. To quantify pMHC valency, we measured the pMHC concentration in the pMHC-NP preps using the Bradford assay (Thermo Scientific).

### pMHCII-NP therapy of NOD and C57BL/6 mice

Cohorts of 10-week-old female NOD mice were injected i.v. with BDC2.5 mi/IA^g7^- or Ins_9-23_/IA^g7^-coated NPs in PBS twice a week for 5 weeks. Cohorts of 8- to 10-week-old male C57BL/6 mice were exposed to 1.5–3.5% dextran sodium sulfate (DSS) in the drinking water on 5- to 7-day cycles, with 5- to 14-day wash-out periods without DSS after each DSS cycle. The mice were treated with 40 µg Fla_462-472_/IA^b^- or Fla_501-514_/IA^b^-NPs i.v. or i.p. twice a week for 7–8 weeks starting at the beginning of the second DSS cycle. Treatment-induced formation and expansion of cognate TR1-like cells were assessed by flow cytometry.

To induce EAE, 8- to 10-wk-old female and male mice were immunized s.c. with 200 μg of pMOG_36–55_ in CFA and administered 300 ng of Pertussis toxin i.v. on days 0 and 3 relative to peptide immunization. Since these mice develop a synchronous nonremitting form of chronic EAE, all the mice were randomized into the treatment groups (Cys-NPs and pMOG_38-49_/IA^b^-NPs −10 µg of pMHC/dose–) when the EAE score reached 1.5. Mice were scored and weighed on Day 0 and then daily from Day 7 after immunization, and scores were plotted on a 5-point scale as described [11].

### Pancreatic islet preparation and tetramer staining

Pancreata were injected with ~2 mL collagenase P (Millipore Sigma, 0.66 mg/mL) through the bile duct. They were then digested at 37 °C for 15 min and dispersed by pipetting. The islets were manually selected under a stereomicroscope and incubated with IL-2-containing LCM overnight in a CO_2_ incubator. The islet cells and islet infiltrating mononuclear cells were further treated with trypsin for 3 min to make single-cell suspensions. For tetramer staining of islet-associated T cells, after Fc receptor (FcR) blocking, cells were stained with InsB_13-21_/IA^g7^ tetramers at 37 °C for 45 min in the presence of anti-CD4, and anti-CD45R/B220 antibodies, and a viability dye was added for the last 15 min.

### Anti-CD3 mAb induction of TR1-like cells

Ten-week-old female NOD mice were treated with two doses of an anti-CD3 mAb (15 µg/dose) i.p. at 0 and 48 h [6]. Splenocytes were collected 4 h later, enriched for CD4^+^ T cells using mAb-coated microbeads, stained with metal-labeled mAbs, and analyzed by mass cytometry or used for cell sorting.

### SRBC immunization, plasma cell enumeration and anti-SRBC antibody tittering

Mice were immunized with sterile citrated SRBC (Cedarlane, CL2580). One milliliter of the SRBC suspension was washed twice with 50 mL of PBS, and the resulting pellet was resuspended in 3.6 mL of PBS. Each animal was immunized i.p. with 0.1 mL of this suspension. Twelve days after immunization, the animals were euthanized, and their spleens and cardiac blood samples were collected. Single-cell splenocyte suspensions were prepared, hemolyzed, filtered and counted using a MoxiFlow cytometer. For plasma cell staining, samples were stored at 4 °C in EasySep buffer or FACS buffer (containing FBS) prior to staining. Then, the samples were incubated with 1:50 Fc block (anti-CD16/CD32) for 10 min at room temperature before washing with 1 mL EasySep or FACS buffer and addition of the antibody cocktail (anti-CD138-BV421, anti-CD267/TACI-PE, anti-CD4-FITC, anti-B220-PerCP, and FVS-780 viability dye (1:100 dilution of each antibody and 1:1000 dilution of viability dye in a total volume of 50 µl per sample, for 30 min at 37 °C). Plasma cells (CD138^+^/CD267^+^) were enumerated upon gating on the lymphocyte subset.

Serial dilutions of serum in PBS (from 1:2 to 1:4096; 0.1 mL per well) were incubated with 0.1 mL of the SRBC suspension diluted to 1% in PBS for 30 min at 37 °C. Titers were calculated after overnight storage of the plates at 4 °C as the dilution of serum falling in the middle of the zone of equivalency (the wells with a positive agglutination reaction but no settling of SRBC at the bottom).

### T-Follicular (TF)-like, TFH, Tetramer^+^ and Tconv cell purification; in vitro stimulation; and cytokine secretion

CD4^+^B220^–^Tetramer^+^ (Tet^+^) or Tetramer^−^ (Tconv) cells were sorted from pMHCII-NP-treated mice by flow cytometry. TF-like cells within the tetramer^−^ CD4^+^ gate were identified by staining with anti-CXCR5 and anti-PD-1 mAbs. Briefly, CD4^+^ T cells were enriched from spleen cell suspensions using an EasySep CD4 Isolation Kit (STEMCELL Technologies, Vancouver, BC), stained with pMHCII tetramers and mAbs and sorted into the tetramer^+^ CD4^+^ and tetramer^−^ CD4^+^CXCR5^hi^PD-1^hi^ subsets by flow cytometry, as described above. The purities of TF and tetramer^+^ cells were 85.8 ± 4.4% and 96.5 ± 1.5% (mean ± SE, *n* = 4), respectively. FACS-sorted cells (10^5^) were challenged with anti-CD3/anti-CD28 mAb-coated beads (Dynabeads T-cell Activator, ThermoFisher, Waltham, MA) for 48 h. Cytokine contents in the supernatants were measured via a Multiplexing LASER Bead Assay (Eve Technologies, AB, Canada). Total RNA was prepared for RNAseq using the RNeasy Plus Mini Kit (Qiagen, Hilden, Germany).

TFH cells (PD-1^hi^CXCR5^hi^) were generated by immunizing NOD mice intraperitoneally with KLH (keyhole limpet hemocyanin) or KLH-DNP (Sigma‒Aldrich, St. Louis, MO, USA) once a week for three consecutive weeks for a total of 3 times (100 µg/dose, CFA + IFA + IFA).

### In vivo TR1 cell formation in TFH cell-transfused hosts

We transfused FACS-sorted CXCR5^hi^PD-1^hi^ CD4^+^ T cells from the spleens of NOD, NOD.*Cd4-Cre* or NOD.*Cd4-Cre/Prdm1*^*loxP/loxP*^ mice (*n* = 5 mice each) treated with 5 doses of BDC2.5 mi/I-A^g7^-NPs (1.5×10^5^/host) into two NOD.*scid* hosts/donor type and treated the hosts with 10 additional doses of pMHCII-NPs and, in some experiments (as indicated in the main text), with an anti-CD25 mAb or rat IgG (BioXCell, 500 ug/dose, i.p.). We performed scRNAseq analysis of the sorted CXCR5^hi^PD-1^hi^ CD4^+^ T-cell pool used for transfer as well as the sorted BDC2.5 mi/I-A^g7^ tetramer^+^ CD4^+^ cells arising in the hosts. Cell cluster assignment was performed using the scRNAseq data obtained for the KLH-DNP-induced TFH.1 cells and the pMHCII-NP-induced Tet^+^ TR1-like and TR1 cells described above.

### In vitro and in vivo analyses of TFH-like function

For the in vitro work, female NOD mice (8–10 wk old; *n* = 10) were injected *i.v*. with 10 doses of BDC2.5 mi/IA^g7^-NPs (two doses per week for 5 weeks). Two days after the last injection, CD4^**+**^ BDC2.5 mi/IA^g7^-tetramer^**+**^CXCR5^hi^PD-1^hi^ cells were isolated from splenocyte suspensions by FACS. Ten additional female NOD mice were immunized *s.c*. with the KLH-DNP conjugate (100 µg, Alpha Diagnostic International) in CFA. B220^**+**^ B cells were sorted from draining lymph nodes of these KLH-DNP-immunized mice 7 days later. Half of the sorted B cells were incubated with BDC2.5 mi peptide (GenScript) at 37 °C for 2 h to promote their cognate interaction with BDC2.5 mi/IA^g7^-specific CD4^+^ T cells expanded by pMHCII-NP treatment. Peptide-pulsed and unpulsed B cells were cultured (5 × 10^4^ cells/well) in the presence or absence of CD4^**+**^ BDC2.5 mi/IA^g7^-tetramer^**+**^CXCR5^hi^PD-1^hi^ T cells (3.5 × 10^4^ cells/well) for 6 days. Cells were harvested and stained with anti-B220-PerCP (BioLegend) and anti-GL7-efluor 450/BV421 (eBioscience) antibodies to identify and enumerate GL7^**+**^ follicular B cells via flow cytometry. The culture supernatants were used to quantify the concentration of DNP-specific IgG via ELISA.

For the in vivo experimentation, female NOD.*Cd4-Cre.Prdm1*^*loxp/loxp*^ mice (8–10 wk old) were treated *i.v*. with 10 doses of BDC2.5 mi/IA^g7^-NPs or control (Cys-conjugated) NPs (2 doses per week for 5 weeks; *n* = 5 mice per NP type). Two days after the last dose, total CD4^**+**^ T cells were isolated from each group of donor mice and transfused into NOD.*Scid* hosts (*n* = 5 mice/NP type; 15 × 10^6^ CD4^**+**^ T cells/host). These NOD.*Scid* hosts were then treated with 5 additional doses of BDC2.5 mi/IA^g7^-NPs or Cys-NPs (2 doses per week for 2.5 weeks). Moreover, NOD mice (*n* = 10) were immunized *i.p*. with KLH-DNP (100 µg) in CFA and boosted 3 weeks later. B cells isolated from these KLH-DNP-immunized mice were pulsed with BDC2.5 mi peptide as described above and then transfused into NOD.*Scid* hosts (20 × 10^6^ cells/host) two days after the last NP dose. The NOD.*Scid* hosts were sacrificed 6 days after B-cell transfer to harvest splenocytes and serum from individual mice. Splenocytes were stained with anti-B220-PerCP (Biolegend), anti-GL7-efluor 450/BV421 (eBioscience) and anti-CD4-FITC antibodies and with BDC2.5 mi/IA^g7^ tetramer-PE to identify GL7^**+**^ follicular B cells and BDC2.5 mi/IA^g7^ tetramer^+^ CD4^+^ T cells, respectively, via flow cytometry. Serum samples were used to quantify the concentration of anti-DNP IgG via ELISA.

The wells of an immunoplate (Thermo Scientific, Nunc) were coated with DNP-BSA (Biosearch Tech.) overnight at room temperature (0.5 μg/mL in PBS, 100 μL/well). The wells were blocked with blocking buffer (PBS containing 1% BSA), washed with washing buffer (PBS containing 0.5% Triton X-100), and then incubated with serially diluted serum or supernatant samples for 2 h at room temperature. After washing, the plates were incubated with rabbit anti-mouse IgG-HRP (Dako) for 2 h at room temperature and washed again. The HRP substrate TMB (100 μL/well) was added to each well, and the reaction was stopped by the addition of 2 N H_2_SO_4_ (50 μL/well). The absorbance (450 nm–570 nm wavelength) was measured using a SpectroMax i3X microplate reader (Molecular Devices).

To evaluate the TFH response to influenza infection, 8–11-week-old male mice (3–5 mice per strain) were injected i.p. with 10^7^ EID_50_ of the HKX31 (H3N2) strain twice at 14-day intervals. Fourteen days after the last infection, sera were collected and analyzed for the presence of H3N2-hemagglutinin-specific IgG and IgM using ELISA. The wells of 96-well plates were coated with 75 ng of recombinant H3N2 HA protein (Sino Biological, Beijing, China) at 4 °C overnight. After blocking with 2.5% FBS for 1 h, they were incubated with 1:400 dilutions of serum for 1 h prior to incubation with anti-mouse IgG-HRP (1:6000 dilution) or anti-mouse IgM-HRP (1:5000 dilution) (Southern Biotec, Birmingham, USA). The absorbance was measured at 450 and 570 nm with TMB/2 N H_2_SO_4,_ and the ODs were calculated at 450–570 nm.

### RNAseq

RNA from tetramer^+^ (CD4^+^B220^−^Tet^+^), Tconv (CD4^+^B220^−^Tet^−^), TFH, Th0 and TF-like cells was isolated from nonstimulated cells, unless indicated otherwise. Cells were sorted in lysis buffer (10^5^ cells). For RNAseq, we generated 4 independent samples containing tetramer^+^ and tetramer^–^ cells from 2 mice each (*n* = 8 total mice). We prepared RNA from 3 independent TFH cell pools (CD4^+^CD44^hi^CXCR5^hi^PD-1^hi^) as well as from Th0 cells (CD4^+^CD44^−^CXCR5^−^PD-1^−^) as a negative control. We sorted 3 samples of TFH cells (55,000, 50,000 and 25,000 cells each) and 3 samples of Th0 cells (100,000 cells each). All samples were obtained from a total of 3 immunized mice. For TF-like cells, we generated 4 independent samples from 2 mice each.

Total RNA was prepared from sorted cells using an RNeasy Plus Mini Kit (Qiagen, Hilden, Germany) and used for preparation of RNAseq libraries and sequencing (Center for Genomic Regulation, CRG, Barcelona, Spain). Libraries were prepared using the TruSeq Stranded mRNA Sample Prep Kit v2 according to the manufacturer’s protocol (Illumina, San Diego, CA, USA). Briefly, 10–50 ng of total RNA was used for poly(A) mRNA purification using streptavidin-coated magnetic beads, and the mRNA was then sheared into fragments of ~300 bp. cDNA was synthesized using reverse transcriptase (SuperScript II, Invitrogen, ThermoFisher Scientific, Waltham, MA, USA) and random primers. The second-strand cDNA incorporated dUTP in place of dTTP. Double-stranded DNA was further used for library preparation. dsDNA was subjected to A-tailing and ligation of the barcoded TruSeq adapters. All purification steps were performed using AMPure XP Beads (Beckman Coulter, Brea, CA, USA). Library amplification was performed by PCR using the primer cocktail supplied in the kit. Final libraries were analyzed using an Agilent DNA 1000 chip to estimate the quantity and size distribution and were then quantified by qPCR using the KAPA Library Quantification Kit (Kapa Biosystems, La Roche, Basel, Switzerland) prior to amplification with Illumina’s cBot system. Libraries were loaded at a concentration of 2.75 pM onto the flow cell and subjected to sequencing with read lengths of 1 × 50 bp on Illumina’s HiSeq 2500 to obtain 30–40 M reads.

### Smartseq2 scRNAseq

Single-cell sorting was performed in SMARTseq2 96-well plates containing lysis buffer. The plates were spun and frozen after sorting and sent to the National Center for Genomic Analysis (CNAG-CRG, Barcelona, Spain) for sequencing and data analysis. Full-length single-cell RNA sequencing libraries and libraries from human T-cell subsets sorted from PBMCs from healthy donors were prepared using a modified Smart-seq protocol [21]. Reverse transcription was performed using SuperScript II (Invitrogen) in the presence of oligo-dT30VN, template-switching oligonucleotides and betaine. cDNA was amplified using the KAPA HiFi HotStart ReadyMix (Kapa Biosystems) and ISPCR primer with 25 cycles of amplification. Following purification with Agencourt AMPure XP beads (Beckmann Coulter), product size distribution and quantity were assessed on a bioanalyzer using a High Sensitivity DNA Kit (Agilent Technologies). Two hundred picograms of the amplified cDNA was amplified with indexed Nextera® PCR primers. The products were purified twice with Agencourt AMPure XP beads and quantified again using a Bioanalyzer High Sensitivity DNA Kit. Sequencing of Nextera® libraries from 384 cells was carried out using one sequencing lane on the Illumina HiSeq2500 v4 or HiSeq4000 platform to obtain 500 K reads/cell.

### 10x scRNA-seq

Cells were partitioned into Gel Bead-In-Emulsions with a target cell recovery of 10,000 total cells. The cell number and viability were verified using a TC20™ Automated Cell Counter (Bio-Rad Laboratories, Hercules, CA, USA). cDNA sequencing libraries were prepared using the NextGEM Single-Cell 3′ mRNA Kit (V3.1; 10X Genomics) following the manufacturer’s instructions. Briefly, after GEM-RT clean up, cDNA was amplified for 13 cycles, and cDNA quality control (QC) and quantification were performed on an Agilent Bioanalyzer High Sensitivity chip (Agilent Technologies). cDNA libraries were indexed by PCR using the PN-220103 Chromiumi7 sample index plate. The size distribution and concentration of the 3′ cDNA libraries were verified on an Agilent Bioanalyzer High Sensitivity chip (Agilent Technologies). Finally, sequencing of cDNA libraries was carried out on the NovaSeq 6000 platform (Illumina) to obtain approximately 25,000–50,000 paired-end 75 bp reads per cell.

### 10x scMultiome analysis

Viable cells (5 × 10^5^) were collected in DMEM (Sigma‒Aldrich) supplemented with 10% FBS (HyClone) at 4 °C and processed for single-cell barcoding and library generation following the manufacturer’s instructions (CG000338; 10X Genomics). Briefly, isolated nuclei were partitioned into Gel Bead-In-Emulsions to produce barcoded cDNA from polyadenylated mRNA as described above, as well as barcoded DNA fragments, and processed for library amplification and sequencing on the NovaSeq 6000 platform (Illumina) as described above.

### Bioinformatic analyses

Bioinformatic analyses of the data were performed using Partek® Flow® software (Partek Inc., St. Louis, MO, USA). For RNAseq, the quality of the fastq files was determined using FastQC v0.11.8 software (http://www.bioinformatics.babraham.ac.uk/projects/fastqc/). Contaminant rRNA was filtered using Bowtie 2 (2.2.5). Reads were aligned with the STAR mapper (version 2.5.3a) to the GENCODE release 16 of the *Mus musculus* genome (mm10 assembly). The raw counts of reads per gene were obtained with the Partek “*Quantify to annotation model (Partek E/M)*” tool. DESeq2 was used to assess differential expression between experimental groups.

For scRNAseq, a quality check of the reads was performed with FastQC v0.11.8 software (Babraham Bioinformatics, Babraham, Cambridge, UK). STAR v2.5.4b was used to align the reads to the mouse reference genome GRCm38 with GENCODE M21 annotations. Gene expression was estimated with RSEM v1.3.0. TCR sequences were reconstructed from Smart-seq data to infer clonality with TraCeR v.0.5.1. We used TraCeR to map the reads to mouse GENCODE release M9 (GRCm38). The parameters –loci A B were used to allow reconstruction of TCRα and TCRβ rearrangements in each cell. Other parameters chosen were -kmerLength 31 and -max_junc_len 50.

For 10x Genomics scRNAseq data, Cell Ranger (version 3.1.0; 10x Genomics) was used to process and demultiplex the raw sequencing data. Raw base call files were first converted to fastq format, and subsequently, the sequences were mapped to the *Mus musculus* genome (version mm10) and demultiplexed to generate single-cell feature counts (using STAR alignment). Downstream analyses, including dimensionality reduction (tSNE), cluster analysis (K-means) and differential expression analysis, were performed using the package Seurat v3 in R and Loupe Browser.

For trajectory inference with Monocle 3, data were projected into a low-dimensional space using UMAP v.0.3.2. Cells were then clustered using Louvain/Leiden community detection to generate a principal graph using an embedding procedure based on the SimplePPT algorithm, which was used as a guide for pseudotime computation.

Single-cell RNA and ATAC multiome demultiplexing, alignment and filtering of the raw sequencing data as well as subsequent barcode counting (UMIs) and peak calling were performed using Cell Ranger ARC (v2.0) software pipelines (10X Genomics). For alignment steps, these pipelines use STAR (10.1093/bioinformatics/bts635) and BWA (10.1093/bioinformatics/btp324). The mouse genome assembly GRCm38 (mm10) was used as the reference. Downstream analysis of gene/fragment-cell matrices was performed using R version 4.1.0 and the Seurat v4.0.3 [22] and Signac v1.3.0 packages [23]. Briefly, low-quality cells were filtered out based on mitochondrial DNA content and UMI count. Both RNA and ATAC sequencing data were normalized, scaled, and dimensionally reduced. Multimodal analysis was performed using a WNN (Weighted Nearest Neighbor) algorithm. tSNE (t-distributed stochastic neighbor embedding) was used for visualization and cell clustering. Differentially expressed genes and enriched chromatin sites were determined using the function ‘FindMarkers’ using the Wilcoxon rank–sum test, applying a log2-fold change threshold of 0.25 and Bonferroni correction of P values.

### Pathway enrichment analyses

Differentially expressed genes (|FC | > 2 and FDR < 0.05) were obtained from 10X Genomics-based scRNAseq data corresponding to TFH- and TR1-like subclusters within sorted Tet^+^ cells from NOD mice treated with BDC2.5 mi/IA^g7^-NPs. Enrichment analysis was performed using the Gene Set Enrichment analysis of Gene Ontology (gseGO) function in the ‘clusterProfiler‘ R package (with adjusted *P* value <0.05).

### ARACNe and VIPER

We built a CD4^+^ T-cell-specific interactome using the Algorithm for Reconstruction of Accurate Cellular Networks (ARACNe) [24] and available gene expression data for CD4^+^ T cells. Gene expression data were downloaded from different GEO repositories containing murine CD4^+^ T-cell samples using the ARCHS4 mining tool (https://amp.pharm.mssm.edu/archs4/). These samples were manually curated (e.g., non-CD4^+^ T cells were eliminated, CD4^+^ T cells under different stimulation conditions were selected, etc.) and gene expression was normalized before use. ARACNe was run with 200 bootstrap iterations to consolidate a final network. This interactome was then used to determine differential transcription factor activity in the tetramer^+^ vs. Tconv comparison using VIPER (Virtual Inference of Protein-activity by Enriched Regulon analysis) [25]. To validate this CD4^+^ T-cell-specific regulon, we ranked TF activity from high to low (relative rank from 0 to 1) and located the validation TFs in this ranked list. Active TFs should be located at the top of the ranked list, while less active TFs should be found at the bottom. Statistical analysis of the enrichment of active and inactive TFs was performed using the GSEA preranked tool [26].

### Statistical analyses

Unless specified otherwise, the sample size values mentioned in the figure legends correspond to the total number of samples examined. Data were compared in GraphPad Prism 6 using the Mann‒Whitney *U* test, chi-square test or one-way or two-way ANOVA. *P* values of <0.05 were considered statistically significant. Only statistically significant *P* values are shown in the figures.

## Results

### pMHCII-NP-induced TR1-like cells express a TFH-like transcriptional profile

**Fig. 1:**
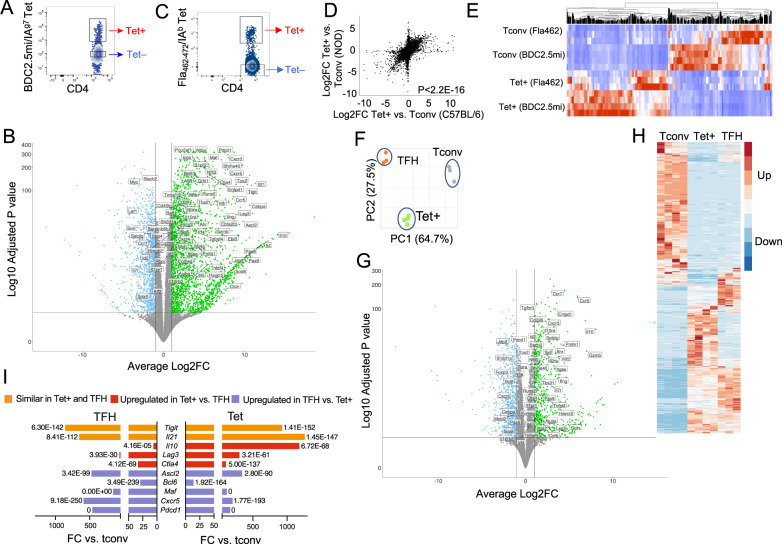
Transcriptional profiling of pMHCII-NP-induced TR1-like vs. Tconv and TFH CD4^+^ T cells. **A** Representative flow cytometric tetramer staining profiles of splenocytes from BDC2.5 mi/IA^g7^-NP-treated NOD mice. **B** Volcano plot showing gene expression differences between BDC2.5 mi/IA^g7^ Tet^+^ and Tet^−^ cells from NOD mice treated with BDC2.5 mi/IA^g7^-NPs. The X-axis shows the log2-fold change values, and the Y-axis shows the −log10 adjusted *P* values. The thresholds for significance were |log2FC | >1 and adjusted *P* value < 0.05. Green, genes significantly upregulated; blue, genes significantly downregulated. Genes among the 106 selected genes from Supplementary Table 1 are labeled. **C** Representative flow cytometric tetramer staining profile of Fla_462-472_/IA^b^-NP-induced TR1-like cells in C57BL/6 mice exposed to 2–3% dextran sodium sulfate (DSS) in the drinking water (to induce colitis). **D** Correlations between gene expression levels (log2FC values) in BDC2.5 mi/IA^g7^-NP- and Fla_462-472_/IA^b^-NP-induced TR1-like cells (from NOD and C57BL/6 mice, respectively) compared to their Tet^–^ counterparts. The data correspond to 3–4 Tet^−^ and Tet^+^ samples per specificity from 2 experiments. **E** Heatmap comparing Tet^+^ and Tet^−^ cells from BDC2.5 mi/IA^g7^-NP-treated NOD and Fla_462-472_/IA^b^-NP-treated B6 mice, showing similar RNAseq profiles for both subsets of cells in both genetic backgrounds. **F** Principal component analysis (PCA) plot for 3 TFH (orange), 3 Tconv (gray) and 4 BDC2.5 mi/IA^g7^-NP-induced Tet^+^(green) samples from 2 experiments. **G** Volcano plot showing gene expression differences between BDC2.5 mi/IA^g7^ Tet^+^ cells from NOD mice treated with BDC2.5 mi/IA^g7^-NPs and KLH-DNP-induced TFH cells in immunized NOD mice. The X-axis shows the log2FC values, and the Y-axis shows the −log10 adjusted *P* values. The thresholds for significance were |log2FC | >1 and adjusted *P* value < 0.05. Green, genes significantly upregulated; blue, genes significantly downregulated. Genes among the 106 selected genes from Supplementary Table 1 are labeled. **H** Heatmap comparing the expression of the 956 genes that were differentially expressed between BDC2.5 mi/IA^g7^ Tet^+^ and Tconv cells among BDC2.5 mi/IA^g7^ Tet^+^ cells, KLH-induced TFH cells and Tconv cells. The thresholds for significance were |FC | ≥ 4 and FDR ≤ 0.01. **I** Expression fold changes and adjusted *P* values for representative TFH/TR1-related genes in Tet^+^ T cells and TFH cells vs. Tconv cells (x-axis: fold change; labels: adjusted *P* values). *Tigit* and *Il21* were expressed at similar levels in Tet^+^ and TFH cells; *Il10*, *Lag3* and *Ctla4* were upregulated in the Tet^+^ T-cell subset vs. its TFH counterpart; and *Ascl2*, *Bcl6*, *Maf*, *Cxcr5*, and *Pdcd1* were upregulated in TFH vs. Tet^+^ cells. The *P* value in (**D**) was calculated via Pearson correlation analysis. The *P* values in I correspond to the FDR values calculated using the DESeq2 tool

To gain an unbiased insight into the nature of the TR1-like CD4^+^ T-cell pools that arise in response to pMHCII-NP therapy, we profiled the transcriptome of murine type 1 diabetes (T1D)-relevant TR1-like CD4^+^ cells induced with BDC2.5 mi/IA^g7^-NP in vivo via RNAseq (twice a week for 5 weeks, starting at 10 weeks of age, when there is full-blown islet inflammation and most T1D-relevant autoantigenic specificities, including BDC2.5mi-specific CD4^+^ T cells, have undergone activation, a *sine qua non* requirement for pMHCII-NP-induced TR1 cell formation [11]). Splenocytes of treated NOD mice were flow-sorted into the CD4^+^B220^−^tetramer^+^ (Tet^+^) and CD4^+^B220^−^tetramer^–^ (referred to as Tet^−^ or Tconv) populations for phenotypic and transcriptional assays (Fig. 1A).

As expected based on our previous studies [11], these Tet^+^ cells had a memory-like phenotype (CD44^hi^ and CD62L^−^), were CD25^−^ and IL-7R^−^, and coexpressed the coinhibitory receptors PD-1 and LAG-3, the costimulatory molecule ICOS, the chemokine receptor CXCR5, and the TFs c-MAF and T-BET but not GATA-3, FOXP3 or RORγT (Fig. 1A and Supplementary Fig. 1). The RNAseq results confirmed that the Tet^+^ cells were clearly different than their Tet^–^ (Tconv) counterparts, with 956 differentially expressed genes (481 upregulated, including known TR1-like-associated genes, such as *Maf, Il10*, *Il21*, *Pdcd1*, *Ccr5* and *Lag3* [11], and 475 downregulated, including known non-TR1-associated genes such as *Il7r* and *Foxp3* [11] (Datasheet 1).

Unexpectedly, the RNAseq data revealed that the transcriptome of the BDC2.5mi/IA^g7^-NP-induced Tet^+^ pool had remarkable gene expression similarities with TFH cells, as compared to the Tet^–^ (Tconv) cell pool. To facilitate comparisons of the Tet^+^ subset to TFH cells and other Treg cell subsets for the reader, some of the data reported further below focus on 106 genes that were selected using the following criteria: 1) their expression has been described in TR1-like cells (for example, *Lag3, Cd49b, Il10, Tigit, Tim3, Ctla4*); 2) they have been shown to be expressed in other Treg subsets (for example, *Foxp3, Fasl, Ebi3, Areg*); 3) they are associated with TFH or T follicular regulatory (TFR) cell phenotypes/functions (i.e., *Cxcr5, Pdcd1, Ascl2, Bcl6, Tcf7*); or 4) they encode TFs upregulated in pMHCII-NP-induced TR1-like cells compared to Tconv cells. Supplementary Table 1 shows comparisons of the expression of these 106 genes, grouped in terms of function, in Tet^+^ vs. Tet^−^ (Tconv), TFH vs. Tconv, and Tet^+^ vs. TFH cells, along with the corresponding references (not included in the main text because of space limitations). Figure 1B provides a volcano plot highlighting gene expression differences between Tet^+^ and Tconv cells with respect to this 106-gene list. Supplementary Figs. 2, 3 show comparisons of the normalized expression counts for these 106 genes between Tet^+^ and Tconv cells and the changes in their expression in Tet^+^ cells after in vitro activation with anti-CD3/anti-CD28 mAbs, respectively. We note that this Supplementary Table and Figures are provided only to simplify data presentation for the reader, not as a substitute for the complete RNAseq datasets used to draw conclusions. Overall, these observations suggest that pMHCII-NP-induced TR1-like cells express a TFH-like transcriptional profile.

To decipher the possible regulatory interactions that establish the TR1-like transcriptional profile, we identified active TFs in these cells via analysis of TF activity based on gene expression. We first built a CD4^+^ T-cell-specific regulon using the Algorithm for Reconstruction of Accurate Cellular Networks (ARACNe) and available gene expression data for CD4^+^ T cells (Datasheet 2). This regulon was then used to determine differential TF activity in Tet^+^ vs. Tconv cells using VIPER (Virtual Inference of Protein-activity by Enriched Regulon analysis). We validated this CD4^+^ T-cell-specific regulon by performing enrichment analysis with a set of 38 validation TFs from Supplementary Table 1 that have been described to be active or inactive in TR1 cells. TF activity was ranked from high to low (relative ranking from 0 to 1), and the validation TFs were located in this ranked list. The validation TFs expected to be active in TR1 and Tconv cells were clustered mainly at the top or bottom of the ranked list (most active, ranking close to 0) (Supplementary Fig. 4A, left), and the TFs less active in TR1 cells were found primarily at the bottom of the ranked list (Supplementary Fig. 4A, right). Statistical analysis using the GSEA pre-ranked tool yielded a NES value of 2.4 and an adjusted *P* value of 0.0045 for TR1-associated TFs in Tet^+^ cells (Supplementary Fig. 4B) and a NES of −1.49 and an adjusted *P* value of 0.049 for TR1-irrelevant TFs. ARACNe/VIPER thus predicted enriched activity (NES ≥ 2) for 161 TFs, including AHR, BATF, BHLHE40, EGR-2, EOMES, ID2, c-MAF, NFIL3, BLIMP1, RUNX2, T-BET and VDR, and decreased activity (NES ≤ −2) for 198 TFs, including TCF-1, LEF1, BACH2 and FOXP3, in Tet^+^ vs. Tconv cells (Datasheet 3). Thus, there is a strong association between the expression of TFs that are expected to be active in TR1 cells (some of which are also known to be active in TFH cells) vs. Tconv cells and the gene expression profiles of these cells.

### pMHCII-NP-induced TR1-like cells with different antigenic specificities and from different genetic backgrounds express similar transcriptional profiles

We next investigated whether the transcriptional profile of pMHCII-NP-induced TR1-like cells might be influenced by genetic background (NOD vs. C57BL/6), MHC class II allele type (I-A^g7^ vs. I-A^b^) or the nature of the underlying inflammatory process responsible for endogenous T-cell priming (T1D vs. colitis). We examined the transcriptome of pMHCII-NP-induced TR1-like cells in C57BL/6 mice exposed to 2–3% dextran sodium sulfate (DSS) in the drinking water (to induce colitis) and subsequently treated with Flagellin (Fla)_462-472_/IA^b^-NPs, displaying a colitis relevant pMHCII specificity. Tet^+^ and Tet^–^ CD4^+^ T cells (Fig. 1C) were sorted by flow cytometry and processed for RNAseq and downstream analyses. The overall differences in gene expression between the Fla_462-472_/I-A^b^-NP-induced Tet^+^ cell pool and its Tconv counterpart were remarkably similar to those between the BDC2.5 mi/I-A^g7^-NP-induced Tet^+^ pool and the corresponding Tconv cells (Fig. 1D and Datasheet 4). In addition, 768 of the 956 genes that were differentially expressed between BDC2.5 mi/IA^g7^-NP-induced Tet^+^ cells and their Tconv counterparts in NOD mice were expressed at similar levels in the Fla_462-472_/IA^b^- and BDC2.5 mi/IA^g7^-specific T-cell pools (Fig. 1E). Furthermore, as shown in Supplementary Table 2, 97/106 Treg/TFH-related genes in these B6-derived pMHCII-NP-induced Tet^+^ cells had a concordant expression change direction (upregulation vs. downregulation) and/or expression level relative to BDC2.5 mi/IA^g7^-NP-induced Tet^+^ cells. Thus, pMHCII-NPs elicit the formation of cognate TR1-like cell pools with similar transcriptional profiles in different genetic backgrounds.

### pMHCII-NP-induced TR1-like cells are transcriptionally similar to antigen-induced TFH cells

As noted above, a noticeable portion of the genes that are upregulated in pMHCII-NP-induced Tet^+^ cells have been described in TFH and/or TFR cells, for example, the TF genes *Ascl2*, *Batf*, *Bcl6*, *Cebpa*, *Irf4*, *Maf*, *Stat1*, *Stat3*, *Tcf7*, *Tox2*, and *Vdr*; the chemokine receptor gene *Cxcr5*; the costimulator gene *Icos*; the coinhibitory receptor genes *Ctla4*, *Havcr2*, *Lag3*, *Pdcd1*, *and Tigit*; and the cytokine genes *Il10* and *Il21* (Supplementary Table 1 and the corresponding references), raising the possibility that these cells arise from TFH precursors. These ‘TR1-like’ Tet^+^ cells, however, were not TFR cells because they lacked the expression of TFs characteristic of murine TFH or TFR cells, such as *Lef1* and *Foxp3*.

To investigate this potential relationship, we compared the RNAseq profiles of pMHCII-NP-induced Tet^+^ cells (TR1-like) with those of NOD TFH cells (CD4^+^CD44^hi^CXCR5^hi^PD-1^hi^) induced by active immunization with keyhole limpet hemocyanin (KLH) and those of Th0 cells isolated from KLH-immunized mice (CD4^+^CD44^–^CXCR5^–^PD-1^–^ cells, to control for the heterogeneous subset content of the Tet^−^ cells used in our initial comparisons; also referred to as Tconv in the figures for simplicity) (Supplementary Fig. 5A) (Datasheet 5). Overall, the pMHCII-NP-induced Tet^+^ cells were significantly different from the KLH-induced TFH cells (Fig. 1F); direct comparison showed the existence of 737 differentially expressed genes, of which 374 were upregulated and 363 were downregulated, between these cell types (Fig. 1G). Notwithstanding these differences, Tet^+^ and TFH cells shared many upregulated and downregulated genes relative to Tconv (Th0) cells. When we focused on the 956 genes that were differentially expressed in pMHCII-NP-induced Tet^+^ vs. Tconv cells, 658 (69%) behaved identically in TFH and Tet^+^ cells (vs. their respective Tconv subsets) (Fig. 1H). Fifty-six percent of these 658 genes (*n* = 368) were expressed at similar levels in Tet^+^ and TFH cells, whereas 22% (*n* = 146) and 21% (*n* = 144) exhibited significantly higher or lower levels of expression, respectively, in Tet^+^ vs. TFH cells. For example, *Tigit* and *Il21* were expressed at similar levels in Tet^+^ and TFH cells; *Il10*, *Lag3* and *Ctla4* were upregulated in the Tet^+^ T-cell subset vs. its TFH counterpart; and *Ascl2*, *Bcl6*, *Maf*, *Cxcr5*, and *Pdcd1* were upregulated in TFH vs. Tet^+^ cells (Fig. 1I**;** Datasheet 5). Eighty-six percent of the remaining 298 genes from the 956 gene set (*n* = 255) were genes that were not differentially expressed in TFH vs. Tconv cells (Datasheet 6).

This relationship was investigated further by comparing the RNAseq profiles of pMHCII-NP-induced Tet^+^ cells with TFH-like PD-1^hi^CXCR5^hi^ cells within the tetramer-negative gate in nonimmunized mice, as well as by comparing the changes in gene expression in response to TCR/CD28 ligation ex vivo (Supplementary Fig. 5B, C) (Datasheet 7). Under basal conditions, the Tet^+^ and endogenous TFH cell pools differed by only 251 differentially expressed genes. Ex vivo activation of Tet^+^ and endogenous TFH cells changed the expression levels of 2329 and 1507 genes, respectively. Direct comparison of the activated subsets revealed that there were 340 differentially expressed genes, indicating that the two populations, although very similar, are distinct. Comparisons focusing on the 106 genes selected from Supplementary Table 1 further substantiated these expression similarities: 7 shared genes that were upregulated (from the 10 and 12 genes that were upregulated upon stimulation in Tet^+^ and TFH cells, respectively); 57 shared genes with no change in expression level (from the 73 and 68 genes in Tet^+^ and TFH cells, respectively); and 8 shared genes that were downregulated (from the 11 genes in Tet^+^ cells and 11 genes in TFH cells that were downregulated upon stimulation) (Supplementary Figs. 3, 6).

Collectively, these results indicated that pMHCII-NP-expanded TR1-like cells display a TFH-like transcriptional footprint.

### Coexistence of TR1 cells and TFH cells within the pMHCII-NP-induced tetramer^+^ pool

**Fig. 2:**
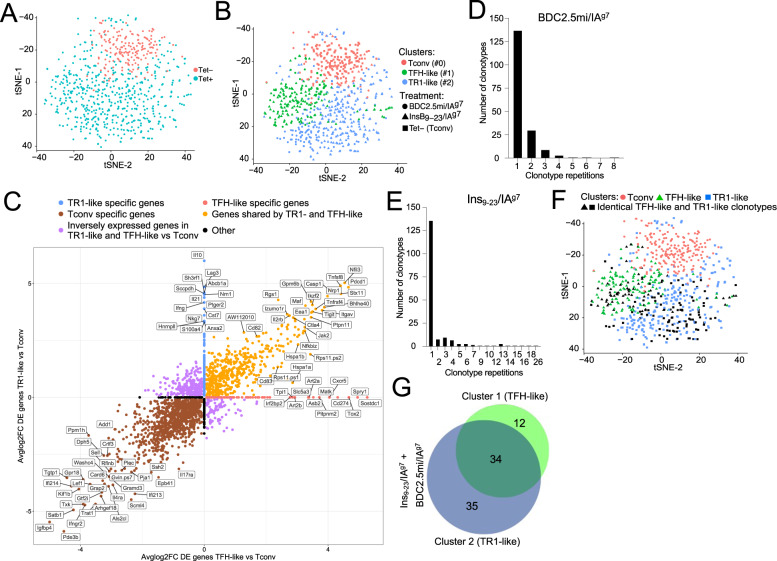
pMHCII-NP-induced TR1-like cells are transcriptionally homogeneous but oligoclonal and coexist with a Tet^+^ TFH-like subpopulation that contains identical clonotypes. **A** t-SNE plot of Smartseq2-based scRNAseq data for sorted Tet^+^ and Tet^−^ cells from NOD mice treated with BDC2.5 mi or InsB_9-23_/IA^g7^-NPs (from *n* = 5 mice for Tet^+^ cells and 15 mice for Tet^−^cells; aliquots of the sorted cells were also used for other experiments). The data are from 4 experiments. **B** Seurat clustering analysis of the Tet^+^ pools from A showed the presence of two clusters for each pMHCII type. **C** Two-dimensional plot of the average log2FC values for the differentially expressed genes (adjusted *P* value < 0.05) between pMHCII-NP-induced Tet^+^ TR1-like, Tet^+^ TFH-like and Tet^−^ cell types pooled from samples from BDC2.5/IA^g7^-NP-treated NOD mice (*n* = 5 mice for Tet^+^ cells and 15 mice for Tet^−^ cells) and samples from Fla_462-472_/IA^b^-NP-treated C57BL/6 mice (*n* = 6 mice for Tet^+^ cells and 5 mice for Tet^−^ cells). The data were obtained by SmartSeq2 scRNAseq in 2 experiments. The X-axis shows Tet^+^ TFH-like vs. Tconv cells, and the Y-axis shows Tet^+^ TR1-like vs. Tconv cells. The dot color represents the cell subset specificity of differential gene expression. Only the genes with the greatest differential expression are labeled. **D**, **E** Distribution of unique TCR sequences in cells in the Tet^+^ pools arising in response to treatment with two different pMHCII-NP types (BDC2.5mi- or InsB_9-23_/IA^g7^-NPs) in NOD mice. The histogram shows the distribution of the different TCRαβ clonotypes identified vs. the number of cells (clones) expressing each TCRαβ pair. The data are from 1 (**D**) and 3 (**E**) experiments. **F** tSNE plot from (**B**) showing the cluster locations for cells with TCRαβ pairs expressed by more than one cell (in black). The data correspond to BDC2.5mi- or InsB_9-23_/IA^g7^-NP-treated mice from 4 experiments. **G** Venn diagram from F showing the distribution of repeated TCRαβ pairs in clusters #1 (TFH-like) vs. #2 (TR1-like). Most (34/46) of the clonotypes found in the TFH-like cluster (#1) were also found in the TR1-like cluster (#2) (34/69)

To ascertain whether the TR1-like Tet^+^ subset induced by pMHCII-NP therapy was a heterogeneous mixture of cells comprising pools of cells at different stages of differentiation, as well as to define the clonality of these cells, we performed single-cell RNA sequencing (scRNAseq), initially via Smartseq2, of sorted Tet^+^ and Tet^−^ cells from NOD mice treated with BDC2.5 mi or InsB_9-23_ epitope-based pMHCII-NPs. As expected, the Tet^+^ and Tet^−^ cells were clustered away from each other (Fig. 2A; Datasheet 8). The scRNAseq profiles of the Tet^+^ T cells from both treatment groups (compared to the Tet^−^ cells) showed significant upregulation of key TR1 signature genes such as the cytokine genes *Il10*, *Il21*, and *Ifng*; the coinhibitory receptor genes *Ctla4*, *Lag3*, *Tigit*, and *Pdcd1*; the costimulatory gene *Icos*; the chemokine genes *Cxcr3* and *Cxcr5*; and the TF genes *Bcl6*, *Maf*, *Ascl2*, *Nfil3* and *Tox2*. These cells also showed downregulation of several non-TR1-associated genes, such as the cytokine receptor gene *Il7r*, the chemokine receptor gene *Ccr7*, and the TF genes *Lef1* and *Klf2* (Supplementary Table 3).

Interestingly, clustering analysis of the normalized data revealed the presence of two clusters within the Tet^+^ pool. Most Tet^+^ cells (63.6%) were placed in cluster #2 (292/459), but a significant portion (36.4%) was placed in cluster #1 (167/459 cells) (Fig. 2B). When compared to the Tet^–^ Tconv cell cluster #0, both Tet^+^ cell clusters (#1 and #2) exhibited significantly downregulated expression of the non-TR1 genes *Ccr7*, *Il7r* and *Lef1*, among others (Datasheet 8). The predominant cluster of Tet^+^ CD4^+^ T cells (#2, herein referred to as TR1-like cells) displayed significant upregulation of many key TR1 markers compared to cluster #0 (Tconv cells) and, to a lesser extent, cluster #1 (referred to as TFH-like cells), including the cytokine genes *Il10*, *Il21*, and *Ifng*; the coinhibitory receptor genes *Ctla4*, *Tigit*, and *Pdcd1*; and the TF genes *Maf*, *Nfil3*, *Prdm1* and *Id2*, all previously found to be upregulated in bulk BDC2.5 mi/IA^g7^-specific TR1-like cells. When compared to cluster #2 (TR1-like), the minor cluster of Tet^+^ T cells (cluster #1, TFH-like) exhibited upregulation of TFH-associated genes, such as *Cxcr5*, *Cxcr4*, *Il4*, *Nfia* and *Tox2* (Datasheet 8). Similar results were obtained for the Tet^+^ cells isolated from Fla_462-472_/IA^b^- and Fla_501-515_/IA^b^-NP-treated C57BL/6 mice (Supplementary Fig. 7A). The plot in Fig. 2C (Datasheet 9) summarizes the most significant differences in gene expression between the pMHCII-NP-induced Tet^+^ TR1-like, Tet^+^ TFH-like and Tet^–^ cell subsets identified in Fig. 2B.

Taken together, these data indicate that the Tet^+^ pool contains both TFH- and TR1-like subpools, suggesting a lineage relationship.

### The TR1 and TFH clusters share identical clonotypes

To ascertain whether the Tet^+^ cells arising in response to treatment were monoclonal or oligo/polyclonal, we analyzed the TCRα and TCRβ sequences expressed in individual cells. The BDC2.5 mi/IA^g7^ tetramer^+^ pool was poly/oligoclonal (Datasheet 10); out of 255 clonotypes sequenced, 137 were unique (Fig. 2D). Likewise, the Ins_9-23_/IA^g7^-specific T-cell pool contained 136 unique clonotypes among the 393 sequenced clonotypes (Datasheet 11) (Fig. 2E). Studies in C57BL/6 mice treated with the two types of Flagellin-based pMHCII-NPs (Fla_462-472_/IA^b^ and Fla_501-515_/IA^b^) revealed a similar degree of poly/oligoclonality (76/322 and 222/378 unique clonotypes for Fla_462-472_/IA^b^ and Fla_501-515_/IA^b^, respectively) (Supplementary Fig. 7B, C) (Datasheets 12, 13).

We next compared the distribution of the repeated clonotypic TCRαβ pairs (i.e., expressed by more than one cell) within the two clusters in the Tet^+^ pool. Remarkably, of the BDC2.5mi- and InsB_9-23_-specific clonotypes with more than one copy that belonged to the TFH- (*n* = 46) or TR1-like (*n* = 69) cluster, 34 were found in both (Fig. 2F, G) (Datasheet 14). Likewise, of the Fla_462-472_/IA^b^- and Fla_501-515_/IA^b^-specific clonotypes with more than one copy that belonged to the TFH-(*n* = 67) or TR1-like (*n* = 70) cluster, 39 were found in both (Supplementary Fig. 7D, E) (Datasheet 15). Thus, the TFH- and TR1-like clusters within these four different Tet^+^ CD4^+^ T-cell pools consistently contained identical clonotypes. The enrichment of unique clonotypes (i.e., found only once, at either the TFH or TR1 cell stage) does not imply that these TFH and TR1 clonotypes do not have TR1 or TFH counterparts, respectively; rather, these “rare” clonotypes are too diluted in the small number of cells that were sequenced (<10,000) to reveal their simultaneous presence at two alternative stages of differentiation.

**Fig. 3:**
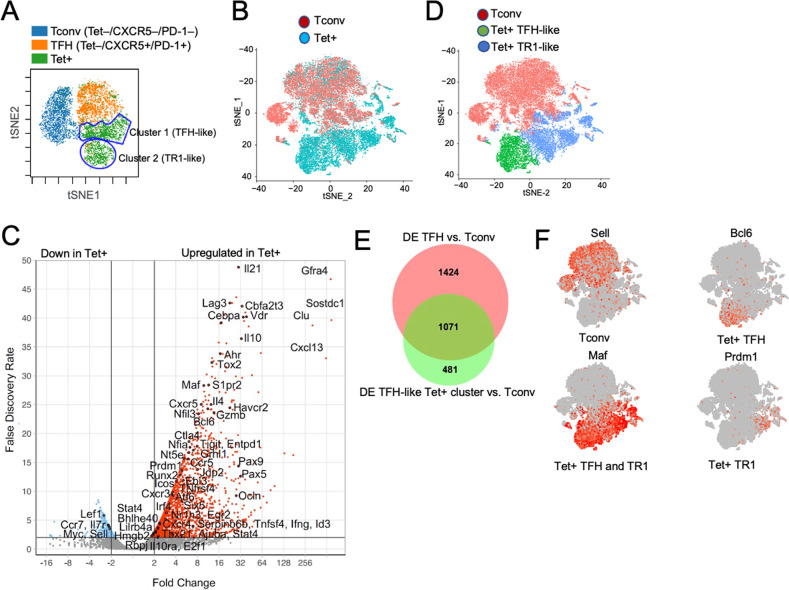
Identification of the tetramer^+^ TFH-like and TR1-like subclusters via mass cytometry and 10x Genomics scRNAseq. **A** tSNE plot for Tet^−^CXCR5^−^PD-1^−^ (Tconv), Tet^−^PD-1^hi^CXCR5^hi^ (TFH) and BDC2.5 mi/IA^g7^ Tet^+^ splenic CD4^+^ T cells stained with the 32-marker CyTOF panel listed in Supplementary Table 4. The data correspond to *n* = 4 samples each from 2 experiments. **B** t-SNE plot of 10x Genomics-based scRNAseq data for sorted Tet^+^ and Tet^–^ cells from NOD mice treated with BDC2.5 mi or InsB_9-23_/IA^g7^-NPs (from *n* = 5 mice for Tet^+^ cells and 15 mice for Tet^−^ cells; aliquots of the sorted cells were also used for other experiments). The data are from 4 experiments. **C** Volcano plots showing the Tet^+^ vs. Tconv comparison (from **C**), with representative TR1-associated and non-TR1-associated genes identified. Red, upregulated genes; blue, downregulated genes. **D** K-means clustering of the cells from (**B**). **E** Venn diagram comparing differentially expressed genes between bulk TFH and Tconv cells from KLH-immunized mice and between Tet^+^ TFH-like and Tet^–^ Tconv cells from pMHCII-NP-treated mice. **F** Representative feature plots for genes enriched in the Tconv, Tet^+^ TFH and Tet^+^ TR1 subclusters or shared between the latter two. The *P* values in (**B**) were calculated by the Mann‒Whitney U test

To ascertain whether the transcriptional TR1-TFH similarity is also reflected at the translational level and to determine whether the Tet^+^ TFH- and TR1-like clusters can be identified at the phenotypic level, we compared the mass cytometry profiles of pMHCII-NP-induced Tet^+^ cells and KLH-DNP-induced TFH (Tet^–^PD-1^hi^CXCR5^hi^) and Tconv cells (Tet^−^PD-1^−^CXCR5^−^). We stained splenic CD4^+^ T cells with 32 TR1/TFH/Treg-related mAbs, including a metal-labeled anti-PE mAb (to mark pMHCII tetramer-PE^+^ cells) and an anti-CD4 mAb (Supplementary Table 4). As shown in Fig. 3A, the Tet^+^ cells from these pMHCII-NP-treated mice could be separated into two major clusters corresponding to the *Il10*-expressing TR1 cluster (cluster #2) and the *Il10*-nonexpressing TFH-like cluster (cluster #1) identified via scRNAseq. TFH cluster #1 lies between the KLH-DNP-induced Tet^–^PD-1^hi^CXCR5^hi^ cell pool and pMHCII-NP-induced Tet^+^ TR1-like cluster #2, again suggesting a lineage relationship between Tet^+^ TFH and TR1 cells, as suggested by scRNAseq analysis.

To further explore the transcriptional relationship between these two Tet^+^ clusters, we performed scRNAseq of larger numbers of FACS-sorted BDC2.5 mi/IA^g7^ and InsB_13-21_/IA^g7^ Tet^+^ and Tconv cells from additional pMHCII-NP-treated animals using the 10x Genomics platform. The pooled Tet^+^ subsets were clearly clustered away from Tconv cells (Fig. 3B, C). Direct comparison of these two different Tet^+^ pMHCII specificities for expression of the 106 TR1/TFH/Treg genes selected from Supplementary Table 1 indicated a high degree of gene expression concordance (Supplementary Table 3) (Datasheet 16). As was the case for the Smartseq2 scRNAseq data, K-means clustering of the 10x Genomics data revealed the presence of two major clusters within the Tet^+^ pool, one expressing high levels of TFH cell markers, such as *Bcl6*, *Ascl2*, *Tox2*, *Cebpa*, *Pdcd1*, *Cxcr5*, and *Il4* (Tet^+^ TFH-like, cluster #1), and a larger pool of cells that (1) expressed markers that were shared between TFH cells and TR1 cells or that were TR1 cell specific, such as *Il10*, *Icos*, *Havcr2*, *Lag3*, *Il21*, *Irf4, Maf, and Prdm1*; and (2) exhibited significant downregulation of TFH-specific cell markers, such as *Cxcr5, Ascl2*, and *Bcl6* (Tet^+^ TR1-like, cluster #2) (Fig. 3D, Supplementary Table 3) (Datasheet 17). Supplementary Fig. 8A, B (Datasheets 18 and 19) show comparisons of the expression levels of the differentially expressed TF, cytokines/chemokine and cytokine/chemokine receptor genes between the Tet^+^ TFH-like and Tet^+^ TR1-like clusters. Supplementary Fig. 8C (Datasheet 20) displays the differentially enriched gene ontology (GO) pathways for the differentially expressed genes between these two Tet^+^ sub-clusters. Together, these data indicate that the Tet^+^ TFH-like and Tet^+^ TR1-like clusters are transcriptionally distinct cell pools with differential upregulation/downregulation of numerous genes that participate in a broad range of biological pathways. Supplementary Fig. 8D displays the most differentially expressed (*P* < 0.02) markers between these two Tet^+^ clusters as determined by our mass cytometry panel.

We then ascertained which of the differences in gene expression that we previously observed between bulk KLH-induced TFH cells and Tconv cells were also shared between the Tet^+^ TFH-like cluster (#1) and the Tconv cluster (#0) in the scRNAseq dataset (Datasheets 21 and 22). As shown in Fig. 3E, F (Datasheet 22) and summarized in Supplementary Table 3 and Supplementary Fig. 9A, D for the 106-gene list, the TFH subset and the Tet^+^ TFH clusters were similar, albeit not identical, suggesting that the Tet^+^ TFH cluster might be a transitional TFH-TR1 pool induced by pMHCII-NP engagement. For example, pMHCII-NP-triggered Tet^+^ TFH-like cells exhibited upregulation of *Havcr2* and *Ahr* and downregulation of *Irf4* compared to KLH-induced TFH cells. When compared to the Tet^+^ TFH-like subpool, the Tet^+^ TR1-like subpool exhibited further upregulation of *Havcr2* and *Ahr* and restored expression of *Irf4*. This Tet^+^ TFH-to-TR1 transition was further accompanied by reductions in or loss of TFH marker expression and gains in TR1 marker expression, consistent with a loss of TFH-ness (*S1pr2, Cxcr4, Cxcr5, Pdcd1, Il4, Ascl2, Bcl6, Cba2t3, Cebpa, Id3, Nfia, Pou2af1, Tox2*) and a gain of TR1-ness (*Ccr5, Havcr2, Il10, Ahr, Myc, Prdm1*).

Together, these data suggest that pMHCII-NP therapy triggers the formation and expansion of oligoclonal subsets of cognate TR1 cells, possibly from cognate TFH cell precursors.

### pMHCII-NP-induced T-cell expansion requires both BCL6 and IRF4, and TR1 formation is regulated by IL-2

**Fig. 4:**
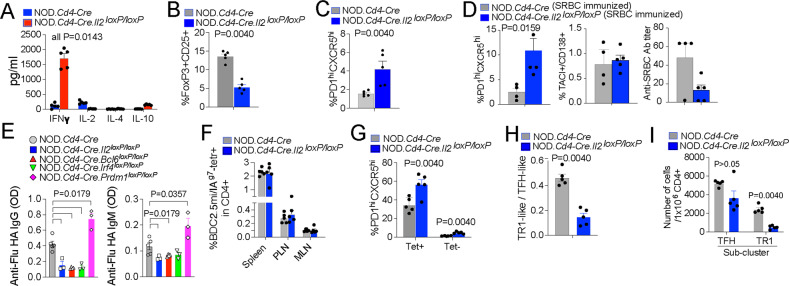
TFH and TR1 cell formation capacity in mice harboring IL-2-deficient T cells. **A** Cytokine secretion profiles of splenic CD4^+^ T cells from NOD.*Cd4-Cre* and NOD.*Cd4-Cre*.*Il2*^*loxP/loxP*^ mice upon stimulation with anti-CD3/anti-CD28 mAb-coated beads. The data correspond to 4–5 mice/strain from 2 experiments. **B** Reduction in the frequency of peripheral CD25^+^FOXP3^+^ CD4^+^ T cells in NOD.*Cd4-Cre*.*Il2*^*loxP/loxP*^ vs. NOD.*Cd4-Cre* mice. The data correspond to n = 5 mice/strain from 3 experiments. **C** NOD.*Cd4-Cre.Il2*^*loxP/loxP*^ mice had increased percentages of endogenous PD-1^hi^CXCR5^hi^ cells in the spleen compared to NOD.*Cd4-Cre* mice. The data correspond to *n* = 5 mice/strain from 3 experiments. **D** Percentages of splenic PD-1^hi^CXCR5^hi^ (TFH) and TACI^+^CD138^+^ (plasma) cells and anti-SRBC antibody titers in NOD.*Cd4-Cre*.*Il2*^*loxP/loxP*^ vs. NOD.*Cd4-Cre* mice upon SRBC immunization. The data correspond to *n* = 5 and 4 mice, respectively, from 2 experiments. **E** Anti-influenza virus IgG and IgM antibody titers (OD value) in 8–11-week-old male mice infected i.p. with 10^7^ EID_50_ of the HKX31 (H3N2) strain twice at a 14-day interval. Fourteen days after the last infection, sera were collected and analyzed for the presence of H3N2-hemagglutinin (HA)-specific IgG and IgM via ELISA. The data correspond to *n* = 5, 3, 4, 3 and 3 mice from left to right. The data correspond to one experiment. **F** Average percentages of BDC2.5 mi/IA^g7^ Tet^+^ CD4^+^ T cells in various lymphoid organs of BDC2.5 mi/IA^g7^-NP-treated NOD.*Cd4-Cre*.*Il2*^*loxP/loxP*^ vs. NOD.*Cd4-Cre* mice. The data correspond to 5 mice/strain from 3 experiments. **G** Average percentages of PD-1^hi^CXCR5^hi^ (TFH) cells within the Tet^+^ and Tet^–^ subsets of the mice in (**F**). **H** Cluster 2 (TR1):cluster 1 (TFH) ratios in the Tet^+^ subsets of BDC2.5 mi/IA^g7^-NP-treated NOD.*Cd4-Cre* and NOD.*Cd4-Cre*.*Il2*^*loxP/loxP*^ mice, as measured via CyTOF. **I** Absolute numbers of cluster 1 (TFH) and cluster 2 (TR1) cells in the Tet^+^ subsets of BDC2.5 mi/IA^g7^-NP-treated NOD.*Cd4-Cre* and NOD.*Cd4-Cre*.*Il2*^*loxP/loxP*^ mice, as measured via CyTOF. The data correspond to 5 mice/strain from 3 experiments. The data in all panels correspond to the mean ± SEM values. *P* values were calculated by the Mann‒Whitney U test

TFH cell-derived IL-2 plays a critical role in non-TFH cell fate decisions, including those of FOXP3^+^ Treg, TH1 and TH2 cells, by promoting the expression of related TFs, cytokines and cytokine receptors [27]. On the other hand, IL-2 inhibits TFH cell development, in part by suppressing BCL6 and promoting BLIMP1 expression [14, 15]. We reasoned that if pMHCII-NP-induced Tet^+^ cells arise and expand from autoantigen-experienced TH1 and/or FOXP3^+^ Treg cells rather than from TFH cells, T-cell deletion of *Il2* would suppress their formation. We thus generated CD4-Cre-transgenic NOD mice carrying two copies of a conditional (loxP-flanked) *Il2* allele (Supplementary Fig. 10). As expected, the splenic CD4^+^ T cells of these mice did not produce IL-2 in response to TCR ligation ex vivo and instead expressed significantly higher levels of IFNγ than their IL-2-competent counterparts, consistent with a T-cell-specific IL-2 deficiency (Fig. 4A). As expected, when compared to NOD.*Cd4-Cre* mice, NOD.*Cd4-Cre.Il2*^*loxP/loxP*^ mice harbored significantly reduced percentages of splenic FOXP3^+^ Treg cells (Fig. 4B and Supplementary Fig. 11A) and increased percentages of endogenous splenic PD-1^hi^CXCR5^hi^ cells (Fig. 4C and Supplementary Fig. 11B). Furthermore, when immunized with sheep red blood cells (SRBCs), or infected with Influenza virus, NOD.*Cd4-Cre.Il2*^*loxP/loxP*^ mice generated increased numbers of TFH cells, albeit not plasma cells, and had decreased anti-SRBC and anti-influenza HA antibody titers, respectively, than NOD.*Cd4-Cre* mice (Fig. 4D, E). The reduction in the anti-SRBC titers despite the increased percentage of TFH-like cells may be mediated by a slight but statistically significant reduction in the level of ICOS on the TFH cells arising in NOD.*Cd4-Cre.Il2*^*loxP/loxP*^ mice (Supplementary Fig. 11C), which has been associated with their B-cell helping capacity [28, 29].

BDC2.5 mi/IA^g7^-NP treatment efficiently triggered the formation and systemic expansion of Tet^+^ CD4^+^ T cells in both NOD.*Cd4-Cre* and NOD.*Cd4-Cre.Il2*^*loxP/loxP*^ mice (Fig. 4F and Supplementary Fig. 11D). Both the Tet^+^ and Tet^–^ subsets from NOD.*Cd4-Cre*.*Il2*^*loxP/loxP*^ mice contained increased percentages of PD-1^hi^CXCR5^hi^ cells, consistent with the increased endogenous TFH cell content (Fig. 4G). In agreement with this, the Tet^–^ subset expressed significantly higher levels of the TFH markers PD-1, ICOS, LAG-3, c-MAF and BCL6 (Supplementary Fig. 11E, F). Among the markers studied, the cognate Tet^+^ cells derived from NOD.*Cd4-Cre*.*Il2*^*loxP/loxP*^ mice also expressed significantly higher levels of PD-1 and BCL6 than those derived from NOD.*Cd4-Cre* mice, as determined by flow cytometry (Supplementary Fig. 11E, G). Together, these data suggest that IL-2 deficiency increases the TFH cell content in the Tet^−^ subset and, to a lesser extent, in the Tet^+^ pool. In fact, mass cytometry analysis of the Tet^+^ cell pools arising in these mice confirmed that those arising in NOD.*Cd4-Cre*.*Il2*^*loxP/loxP*^ mice exhibited lower TR1-like/TR1:TFH-like cell ratios (and significantly smaller pools of TR1-like/TR1 cells but not of TFH-like cells) than those arising in NOD.*Cd4-Cre* mice at both the relative (Fig. 4H) and absolute levels (Fig. 4I), suggesting that IL-2 contributes to the TFH-TR1 conversion.

**Fig. 5:**
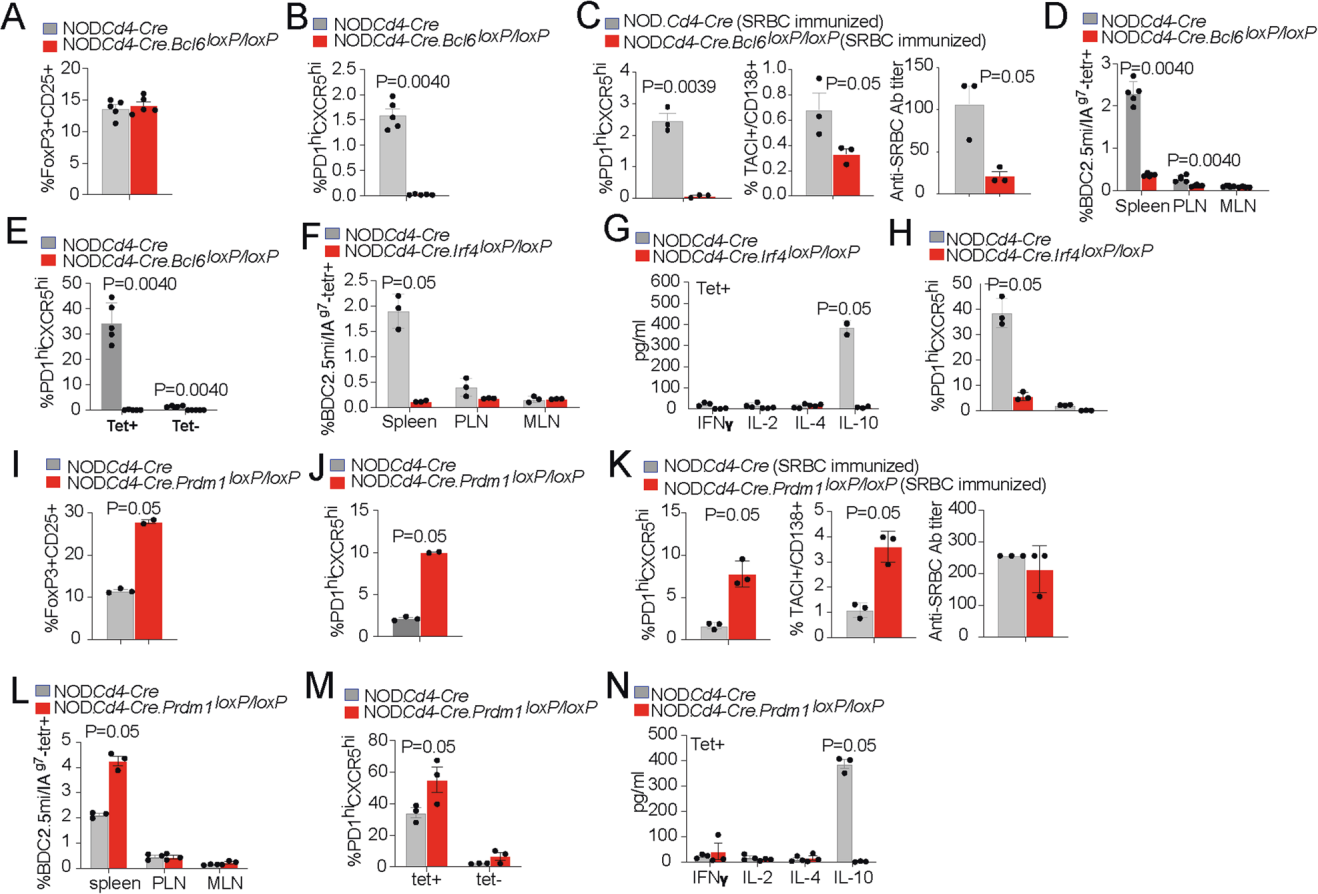
TFH and TR1 cell formation capacity in mice harboring BCL6-, IRF4- or BLIMP1-defficient T cells. **A** NOD.*Cd4-Cre*.*Bcl6*^*loxP/loxP*^ mice had similar percentages of FOXP3^+^CD25^+^ CD4^+^ T cells in the spleen compared to NOD.*Cd4-Cre* mice. The data correspond to 5 mice/strain from 3 experiments. **B** NOD.*Cd4-Cre*.*Bcl6*^*loxP/loxP*^ mice had significantly reduced percentages of endogenous PD-1^hi^CXCR5^hi^ (TFH) cells in the spleen compared to NOD.*Cd4-Cre* mice. The data correspond to 5 mice/strain from 3 experiments. **C** Percentages of splenic PD-1^hi^CXCR5^hi^ (TFH) and TACI^+^CD138^+^ (plasma) cells and anti-SRBC antibody titers in NOD.*Cd4-Cre*.*Bcl6*^*loxP/loxP*^ vs. NOD.*Cd4-Cre* mice upon SRBC immunization. The data correspond to 6 and 5 mice/strain, respectively, from 2 experiments. **D** Percentages of BDC2.5 mi/IA^g7^ Tet^+^ CD4^+^ T cells in various lymphoid organs from BDC2.5 mi/IA^g7^-NP-treated NOD.*Cd4-Cre*.*Bcl6*^*loxP/loxP*^ vs. NOD.*Cd4-Cre* mice. The data correspond to 5 mice/strain from 3 experiments. **E** Average percentages of PD-1^hi^CXCR5^hi^ (TFH) cells within the Tet^+^ and Tet^–^ subsets of the mice in (**D**). **F** Percentages of BDC2.5 mi/IA^g7^ Tet^+^ CD4^+^ T cells in various lymphoid organs from BDC2.5 mi/IA^g7^-NP-treated NOD.*Cd4-Cre*. *Irf4*^*loxP/loxP*^ vs. NOD.*Cd4-Cre* mice. The data correspond to 3 mice/strain from 2 experiments. **G** Cytokine secretion profiles of splenic Tet^+^ CD4^+^ T cells from the mice in (**F**) upon stimulation with anti-CD3/anti-CD28 mAb-coated beads. The data correspond to 3 mice/strain. **H** Average percentages of PD-1^hi^CXCR5^hi^ (TFH) cells within the Tet^+^ and Tet^–^ subsets of the mice in (**F**). **I** Increase in the frequency of peripheral CD25^+^FOXP3^+^ CD4^+^ T cells in NOD.*Cd4-Cre*.*Prdm1*^*loxP/loxP*^ vs. NOD.*Cd4-Cre* mice. The data correspond to *n* = 3 and 2 mice/strain, respectively, from 2 experiments. **J** Increased percentages of endogenous PD-1^hi^CXCR5^hi^ cells in the spleen of NOD.*Cd4-Cre.Prdm1*^*loxP/loxP*^ mice compared to NOD.*Cd4-Cre* mice. The data correspond to *n* = 3 and 2 mice/strain, respectively, from 2 experiments. **K** Percentages of splenic PD-1^hi^CXCR5^hi^ (TFH) and TACI^+^CD138^+^ (plasma) cells and anti-SRBC antibody titers in NOD.*Cd4-Cre*.*Prdm1*^*loxP/loxP*^ vs. NOD.*Cd4-Cre* mice upon SRBC immunization. The data correspond to *n* = 4 and 3 mice/strain, respectively, from 1 experiment. **L** Percentages of BDC2.5 mi/IA^g7^ Tet^+^ CD4^+^ T cells in various lymphoid organs from BDC2.5 mi/IA^g7^-NP-treated NOD.*Cd4-Cre*.*Prdm1*^*loxP/loxP*^ vs. NOD.*Cd4-Cre* mice. The data correspond to 3 mice/strain from 2 experiments. **M** Average percentages of PD-1^hi^CXCR5^hi^ (TFH) cells within the Tet^+^ and Tet^–^ subsets of the mice in (**L**). **N** Cytokine secretion profiles of splenic Tet^+^ CD4^+^ T cells from NOD.*Cd4-Cre* and NOD.*Cd4-Cre*.*Prdm1*^*loxP/loxP*^ mice upon stimulation with anti-CD3/anti-CD28 mAb-coated beads. The data correspond to 3 mice/strain from 2 experiments. The data in all panels correspond to the mean ± SEM values. *P* values were calculated by the Mann‒Whitney U test

We then investigated the role of BCL6 in pMHCII-NP-induced TR1 cell formation. As noted above, BCL6 is the master regulator of TFH cell differentiation [14–16] and was expressed in the Tet^+^ TFH- but not the TR1-like cell cluster (Fig. 3F). As expected, NOD.*Cd4-Cre.Bcl6*^*loxP/loxP*^ mice harbored similar percentages of CD25^+^FOXP3^+^ Treg cells (Fig. 5A and Supplementary Fig. 11H) but significantly lower percentages of endogenous TFH-like cells (Fig. 5B and Supplementary Fig. 11I) than NOD.*Cd4-Cre* mice. In addition, these mice could not efficiently generate TFH cells, plasma cells, anti-SRBC-specific antibodies or anti-influenza HA-specific antibodies upon immunization with SRBC cells or infection with Influenza virus (Figs. 4E, 5C). Thus, NOD.*Cd4-Cre*.*Bcl6*^*loxP/loxP*^ mice, unlike NOD.*Cd4-Cre*.*Il2*^*oxP/loxP*^ mice are TFH cell deficient.

Upon BDC2.5 mi/IA^g7^-NP treatment, NOD.*Cd4-Cre*.*Bcl6*^*loxP/loxP*^ mice harbored tenfold fewer Tet^+^ T cells than NOD.*Cd4-Cre* mice, accompanied by a near-complete absence of TFH-like cells within the Tet^+^ and Tet^–^ gates (Fig. 5D, E and Supplementary Fig. 11J). Analyses of TFH/TR1 subpool formation as a function of dose number (via CyTOF) indicated that the greatly reduced pool of Tet^+^ cells arising in NOD.*Cd4-Cre*.*Bcl6*^*loxP/loxP*^ mice was not an artifact of altered kinetics of cognate TR1 cell formation in the absence of BCL6 (Supplementary Fig. 11K). Specifically, studies of NOD.*Cd4-Cre* mice treated with 0, 1, 3, 5, 7 and 10 doses of pMHCII-NPs showed that treatment triggered progressive increases in both the absolute number of pMHCII-NP-induced Tet^+^ TFH- and TR1-like cells and in the Tet^+^ TR1:TFH-like cell ratio as a function of dose number. This effect was not observed in NOD.*Cd4-Cre*.*Bcl6*^*loxP/loxP*^ mice (Supplementary Fig. 11K, L). Since the few Tet^+^ cells that arose in NOD.*Cd4-Cre*.*Bcl6*^*loxP/loxP*^ mice expressed BCL6 (Supplementary Fig. 11M), we suspect that they arose from cell precursors in which Cre-mediated deletion of *Bcl6*^*loxP*^ was incomplete.

A similar outcome was observed in NOD.*Cd4-Cre*.*Irf4*^*loxP/loxP*^ mice, which lack expression of interferon regulatory factor 4 (IRF4), a molecule implicated in both TFH and TR1 differentiation and function [30, 31]; treatment of these mice with BDC2.5 mi/IA^g7^-NP failed to induce Tet^+^ CD4^+^ T-cell expansion (Fig. 5F, G and Supplementary Fig. 11N), accompanied by a significant reduction in the TFH-like cell content in the Tet^−^ pool (Fig. 5H) and impaired anti-influenza HA antibody production (Fig. 4E). Thus, pMHCII-NP-induced expansion of Tet^+^ cells requires the presence of TFH cells.

### BLIMP1-dependent TFH-to-TR1 cell reprogramming

Several clues suggested that BLIMP1, expressed in the Tet^+^ TR1 subpool but not in its Tet^+^ TFH counterpart, might be responsible for orchestrating the TFH-to-TR1 cellular conversion event described above. In B cells, BCL6 represses BLIMP1 expression, and upregulation of BLIMP1 silences the BCL6-induced B-cell transcriptional program, including the expression of *Pax5, Bach2*, *Bcl6* and *Id3* [18] (all of which were also downregulated in the TR1 cluster compared with the TFH cluster described here). In TFH cells, as in mature B cells, BCL6 represses BLIMP1 expression [17]. BCL6 expression in TFH cells is induced (and BLIMP1 expression is antagonized) by LEF1 (which was not expressed in the pMHCII-NP-induced TFH or TR1 subpool; Supplementary Table 3) and TCF1 (encoded by *Tcf7* and expressed in both Tet^+^ subpools but significantly downregulated in the TR1 subpool; Supplementary Table 3) [32]. We thus reasoned that repetitive encounters of cells in the transitional TFH subpool with pMHCII-NPs might trigger downregulation of TCF1 and thus promote downregulation of BCL6 and upregulation of BLIMP1, which are known to antagonize TFH formation [15, 33].

To investigate a potential role for BLIMP1 in the TFH-to-TR1 conversion, we produced and studied NOD.*Cd4-Cre*.*Prdm1*^*loxP/loxP*^ mice. When compared to NOD.*Cd4-Cre* mice, NOD.*Cd4-Cre Prdm1*^*loxP/loxP*^ mice displayed an ~2-fold increase in the splenic FOXP3^+^ Treg cell content (Fig. 5I and Supplementary Fig. 11O) and an ~5-fold increase in the percentage of endogenous splenic PD-1^hi^CXCR5^hi^ cells (Fig. 5J and Supplementary Fig. 11P). Perhaps due to the large increase in the FOXP3^+^ Treg cell content and despite the key role of BLIMP1 in *Il10* expression, NOD.*Cd4-Cre Prdm1*^*loxP/loxP*^ mice developed neither type 1 diabetes (none of the 50 females and 21 males developed T1D for at least 28 wk of follow-up) nor colitis (no clinical signs of ulcerative colitis in any of the above mice and no pathological features of colitis in the three mice that were examined histopathologically; data not shown). In fact, the pancreata of these mice (at 10 wk of age) appear to contain significantly more and larger islets than those of NOD.*Cd4-Cre* mice, and these islets harbored slightly lower percentages of Ins_13-21_/IA^g7^-specific CD4^+^ T cells than those isolated from NOD.*Cd4-Cre* mice (Supplementary Fig. 11Q). In agreement with the increased TFH cell content, these mice generated increased numbers of TFH cells and plasma cells and an increased anti-influenza HA antibody titer upon influenza infection (despite the similar anti-SRBC antibody titers after immunization with SRBCs) (Figs. 4E, 5K). In addition, BDC2.5 mi/IA^g7^-NP treatment generated approximately twice as many cognate CD4^+^ T cells in NOD.*Cd4-Cre.Prdm1*^*loxP/loxP*^ mice than in NOD.*Cd4-Cre* control mice (Fig. 5L and Supplementary Fig. 11R), and both the Tet^+^ and Tet^−^ subsets from NOD.*Cd4-Cre.Prdm1*^*loxP/loxP*^ mice contained increased percentages of PD-1^hi^CXCR5^hi^ cells (Fig. 5M), consistent with the very large increase in the endogenous TFH cell content mentioned above (Fig. 5J). Phenotypically, the total Tet^+^ (and Tet^−^) cells from these NOD.*Cd4-Cre.Prdm1*^*loxP/loxP*^ mice were distinct from those arising in NOD*.Cd4-Cre* mice, as they expressed lower levels of LAG-3 and increased levels of T-BET, PD-1 and CXCR5 (Supplementary Fig. 11S) and could not produce IL-10 in response to anti-CD3/anti-CD28 mAb stimulation ex vivo, indicating a key role for BLIMP1 in pMHCII-NP-induced TR1 cell formation (Fig. 5N).

**Fig. 6:**
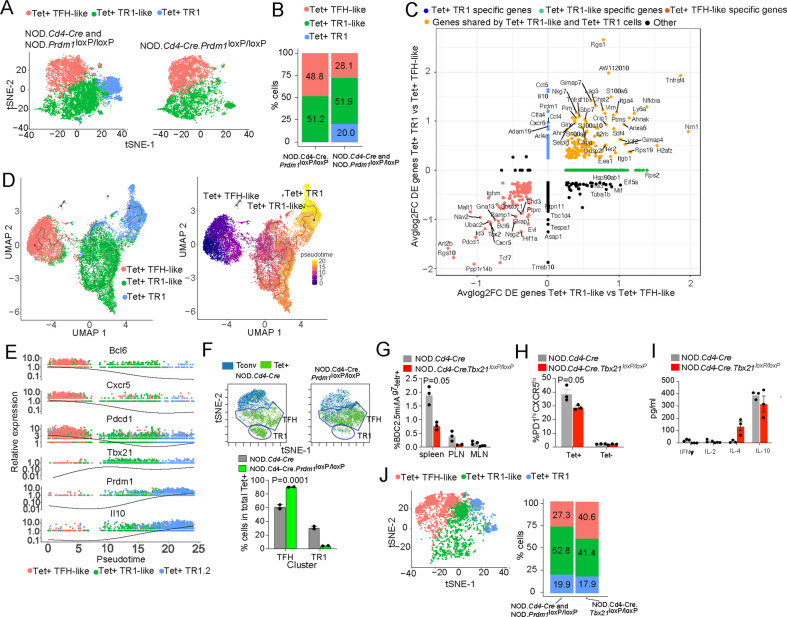
BLIMP1-dependent TFH-to-TR1 cell reprogramming. **A** tSNE plots of 10x Genomics scRNAseq data for sorted BDC2.5 mi/IA^g7^ tetramer^+^ (Tet^+^) cells from BDC2.5 mi/IA^g7^-NP-treated NOD.*Cd4-Cre* (*n* = 3) and NOD.*Prdm1*^*loxP/loxP*^ (*n* = 3) mice vs. NOD.*Cd4-Cre.Prdm1*^*loxP/loxP*^ (*n* = 2) mice from 1 experiment. Cell subsets were identified via k-means clustering and prediction using the 10x Genomics scRNAseq data from Fig. 3C as a reference. **B** Fraction of total cells corresponding to each Tet^+^ subcluster. **C** Two-dimensional plot of the average log2FC values for the differentially expressed genes in the terminally differentiated TR1 vs. TFH (y-axis) vs. TR1-like vs. TFH (x-axis) comparisons in *Prdm1*-competent mice. **D** Trajectory analysis results on UMAP plots generated with 10x Genomics scRNAseq data from BDC2.5 mi/IA^g7^ tetramer (Tet^+^) cells isolated from BDC2.5 mi/IA^g7^-NP-treated NOD.*Cd4-Cre* and NOD.*Prdm1*^*loxP/loxP*^ mice. Left, cell cluster identity; right, pseudotime analysis. **E** Relative expression levels of representative TFH- and TR1-specific genes vs. pseudotime. **F** Top, tSNE plot for Tet^–^CXCR5^–^PD-1^−^ (Tconv) and BDC2.5 mi/IA^g7^ Tet^+^ splenic CD4^+^ T cells (Tet^+^) from BDC2.5 mi/IA^g7^-NP-treated NOD.*Cd4-Cre* and NOD.*Prdm1*^*loxP/loxP*^ mice stained with the 32-marker CyTOF panel listed in Supplementary Table 4. Bottom, average % of cells ±S.E.M. values in clusters #1 and #2. The data correspond to 2 mice/strain from 1 experiment. **G** Percentages of BDC2.5 mi/IA^g7^ Tet^+^ CD4^+^ T cells in various lymphoid organs from BDC2.5 mi/IA^g7^-NP-treated NOD.*Cd4-Cre*.*Tbx21*^*loxP/loxP*^ vs. NOD.*Cd4-Cre* mice. The data correspond to 3 mice/strain from 3 experiments. **H** Average percentages of PD-1^hi^CXCR5^hi^ (TFH) cells within the Tet^+^ and Tet^–^ subsets of the mice in (**G**). **I** Cytokine secretion profiles of splenic Tet^+^ CD4^+^ T cells from the mice in (**G**) upon stimulation with anti-CD3/anti-CD28 mAb-coated beads. **J** Left: tSNE plots of 10x Genomics scRNAseq data for sorted BDC2.5 mi/IA^g7^ tetramer (Tet^+^) cells from BDC2.5 mi/IA^g7^-NP-treated NOD.*Cd4-Cre.Tbx21*^*loxP/loxP*^ mice (*n* = 3) from 1 experiment. Right: Fraction of total cells corresponding to each Tet^+^ subcluster in BDC2.5 mi/IA^g7^-NP-treated NOD.*Cd4-Cre.Tbx21*^*loxP/loxP*^ vs. control (NOD.*Cd4-Cre* and NOD.*Prdm1*^*loxP/loxP*^) mice. The data in (**G**–**I**) correspond to the mean ± SEM values. The *P* values in (**F**) and (**G**–**I**) were calculated via two-way ANOVA and the Mann‒Whitney U test, respectively

To further explore the effects of *Prdm1* deletion on pMHCII-NP-induced TR1 cell formation, we compared the scRNAseq profiles of BDC2.5 mi/IA^g7^ Tet^+^ cells isolated from pMHCII-NP-treated NOD.*Cd4-Cre.Prdm1*^*loxP/loxP*^ mice, with the scRNAseq profiles of Tet^+^ cells isolated from NOD.*Prdm1*^*loxP/loxP*^ mice and NOD.*Cd4-Cre* mice. Remarkably, the Tet^+^ pool from NOD.*Cd4-Cre.Prdm1*^*loxP/loxP*^ mice contained both Tet^+^ TFH- and TR1-like cells but had a twofold increase in Tet^+^ TFH cells and was completely devoid of a TR1-like cell subcluster (herein referred to as terminally differentiated ‘TR1’ cells), which accounted for ~20% of the total Tet^+^ cells in *Pdrm1*-competent mice (Fig. 6A, B) (Datasheet 23). We refer to the transitional and terminally differentiated TR1-like cells as ‘TR1-like’ and ‘TR1’, respectively, because they share the expression of many of the transcripts that distinguish TR1-like cells from TFH cells. The two-dimensional plot in Fig. 6C shows the genes with the greatest differences in expression among the three Tet^+^ cell clusters in *Prdm1*-competent mice, including TR1 subcluster-specific upregulation of the key TR1 cell markers *Ccl5, Il10, Prdm1* and *Ctla4*. Supplementary Table 5 and Supplementary Fig. 12 show comparisons of the expression levels of the 106 TFH/TR1-related genes among these three clusters. Importantly, the results of cell trajectory analysis using Monocle 3 were consistent with a TFH-like –>TR1-like –>TR1 differentiation trajectory (Fig. 6D), involving loss of TFH-related genes, such as *Bcl6*, *Cxcr5* and *Pdcd1*, in TR1-like and TR1 cells; upregulation of *Tbx21* at the TR1-like cell stage; and upregulation of *Prdm1* and *Il10* at the terminally differentiated TR1 stage (Fig. 6E). Specifically, TR1 cells exhibited significant upregulation or downregulation of 1,515/18,867 genes, including 25 of the 106 genes from Supplementary Table 1 (Fig. 6C) (Datasheet 24).

The above observations were further confirmed via mass cytometry. As shown for NOD mice in Fig. 3A, the Tet^+^ cells from pMHCII-NP-treated NOD.*Cd4-Cre* mice could be separated into TFH- and TR1-like clusters. When compared to pMHCII-NP-treated NOD.*Cd4-Cre* mice, pMHCII-NP-treated NOD.*Cd4-Cre*.*Prdm1*^*loxP/loxP*^ mice harbored significantly increased percentages of TFH-like cells at the expense of a significant reduction in the percentage of TR1-like cells (Fig. 6F).

Collectively, these results indicated that the pMHCII-NP-induced TFH-to-TR1 cell transition evolves through a transitional *Il10*^*–*^*Cxcr5*^–*low*^*Ccr5*^*−*^*Prdm1*^−^*Bcl6*^*low*^ TR1-like cell stage. De novo expression of *Pdrm1* in TR1-like cells upon the loss of BCL6 and TCF1 expression results in terminal differentiation of these cells into immunoregulatory *Il10*^*+*^*Cxcr5*^*−*^*Ccr5*^*+*^*Prdm1*^*+*^*Bcl6*^*–*^ TR1 cells, such that abrogation of *Prdm1* expression stalls the Tet^+^ TFH to TR1 cell transition. Most importantly, the complete absence of terminally differentiated TR1 cells in the tetramer^+^ CD4^+^ T-cell pools arising in pMHCII-NP-treated NOD.*Cd4-Cre.Prdm1*^*loxP/loxP*^ mice demonstrates that TR1 cells arise from TFH/TR1-like cell precursors, and that TFH/TR1-like cells do not arise from TR1 cell precursors. This interpretation is consistent with the results of experiments in NOD.*Cd4-Cre*.*Bcl6*^*loxP/loxP*^ and NOD.*Cd4-Cre*.*Irf4*^*loxP/loxP*^ mice, which are incompatible with a TR1-to-TFH direction of differentiation (i.e., TR1 cells acquiring a TFH-like phenotype). These data (and the data described further below) are also incompatible with an independent origin of TFH and TR1 cells from a common noncommitted precursor. Supplementary Fig. 13 (Datasheet 25) shows a schematic diagram that summarizes the key transcriptional and phenotypic changes that occur along the Tet+ TFH-TR1 cell transdifferentiation pathway.

### pMHCII-NP-induced TR1 cell formation is T-BET-independent

The above observations, implicating the TFH lineage as a source of TR1 cells, seemed to be inconsistent with the prevailing view that TR1 cells arise from chronically activated TH1 cells (reviewed in [9]). To further exclude a role for TH1 cells as precursors of pMHCII-NP-induced TR1 cells, we tested the pharmacodynamic activity of BDC2.5mi-IA^g7^-NPs in NOD.*Cd4-Cre.Tbx21*^*loxP/loxP*^ mice, which lack the expression of T-BET, required for TH1 cell development [34]. Although T-cell-specific deletion of T-BET reduced the magnitude of BDC2.5 mi/IA^g7^-NP-induced Tet^+^ cell expansion (Fig. 6G and Supplementary Fig. 11N), coinciding with a significant decrease in the TFH-like cell content in the Tet^+^ pool (Fig. 6H), it did not significantly impair TR1 cell formation, based on both IL-10 production (Fig. 6I) and scRNAseq profiling of Tet^+^ cells (Fig. 6J). However, the absence of T-BET skewed the TFH:TR1-like cell ratio in favor of TFH cells (1.93 vs. 1.01 in wild-type vs. NOD.*Cd4-Cre.Tbx21*^*loxP/loxP*^ mice), suggesting a role for T-BET in TFH-to-TR1-like cell differentiation. The relative timing of *Tbx21* expression along the TFH-like –>TR1-like –>TR1 axis supports this possibility; expression of *Tbx21* peaked at the TR1-like cell stage, and its level was significantly higher than that in TFH cells (Fig. 6E). As expected, the Tet^+^ cells arising in these mice did not express IFNγ and exhibited significant upregulation of IL-4 compared to the Tet^+^ cells arising in control mice (Fig. 6I), suggesting a role for TR1 cell-intrinsic T-BET in transcriptional regulation in TR1 cells. These observations are compatible with our previous observations in NOD.*Ifng*^−/−^ mice, where pMHCII-NP therapy triggered the expansion of Tet^+^ cells with an altered cytokine profile (coexpression of both IL-10 and IL-4), consistent with IFNγ-mediated suppression of IL-4 expression [11]. Thus, T-BET contributes to pMHCII-NP-induced TFH cell expansion and TR1-like cell formation but is dispensable for pMHCII-NP-induced formation of TR1 cells.

### Anti-CD3 mAb-induced TR1-like cell formation is also BCL6 and BLIMP1 dependent

**Fig. 7:**
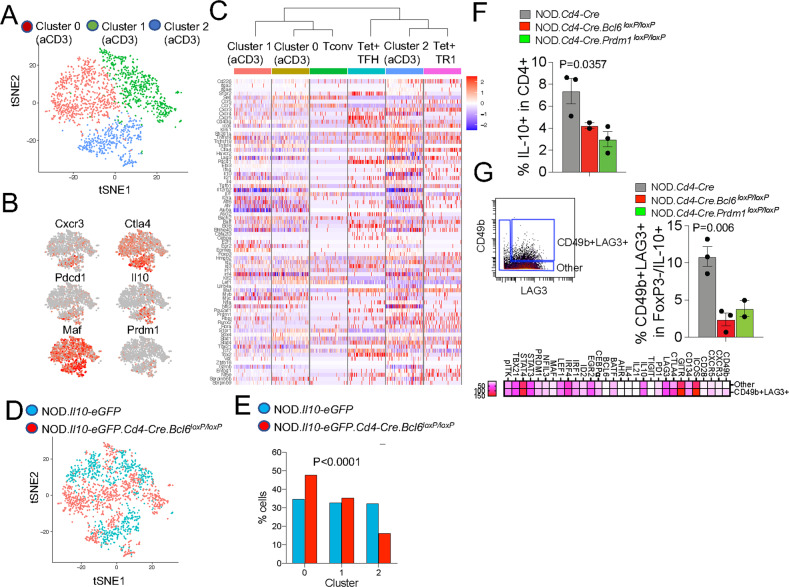
BCL6 and BLIMP1 dependency of anti-CD3-induced TR1-like cells. **A** K-means clustering of splenic IL-10^+^ (eGFP^+^) cells isolated from anti-CD3 mAb-treated NOD.*Il10-eGFP* (*Bcl6*^+/+^) mice, based on 10x Genomics scRNAseq data. The data correspond to 2 mice from 1 experiment. **B** Feature plots for representative TR1 cell markers in the tSNE plots shown in (**A**). **C** Heatmap comparing clusters #0, #1 and #2 from (**A**) to the BDC2.5 mi/IA^g7^-NP-induced Tet^+^ TFH and Tet^+^ TR1-like subclusters from Fig. 3. The dendrogram was generated using the BuildClusterTree function of Seurat. **D**, tSNE plots of 10x Genomics scRNAseq for splenic IL-10^+^ (eGFP^+^) cells isolated from anti-CD3 mAb-treated NOD.*Il10-eGFP* (*Bcl6*^+/+^) and NOD.*Il10-eGFP*.*Cd4-Cre.Bcl6*^loxP/loxP^ mice. The data correspond to 2 mice/strain. **E** Percentages of cells in clusters #0, #1 and #2 in the mice from (**D**). **F** Average percentages of IL-10^+^ CD4^+^ T cells in anti-CD3 mAb-treated NOD.*Cd4-Cre*.*Bcl6*^*loxP/loxP*^ and NOD.*Cd4-Cre.Prdm1*^*loxP/loxP*^ vs. NOD.*Cd4-Cre* mice, as determined using the mass cytometry marker panel from Supplementary Table 4 (with the anti-CD49b-PE antibody instead of pMHCII tetramer-PE). The data correspond to 3 (NOD.*Cd4-Cre*.*Bcl6*^*loxP/loxP*^), 2 (NOD.*Cd4-Cre.Prdm1*^*loxP/loxP*^) and 3 (NOD.*Cd4-Cre*) mice/strain from 3 experiments. **G** Average percentages of CD49b^+^LAG-3^+^ cells in the splenic IL-10^+^FOXP3^−^ CD4^+^ T-cell pools of anti-CD3 mAb-treated NOD.*Cd4-Cre*.*Bcl6*^*loxP/loxP*^ and NOD.*Cd4-Cre.Prdm1*^*loxP/loxP*^ vs. NOD.*Cd4-Cre* mice as determined by mass cytometry. Top left, gating strategy; bottom, heatmap comparing the expression levels of the various markers; top right, average % values ± S.E.M. values. The data correspond to 3 (NOD.*Cd4-Cre.Bcl6*^*loxP*/*loxP*^), 2 (NOD.*Cd4-Cre.Prdm1*^*loxP/loxP*^) and 3 (NOD.*Cd4-Cre*) mice/strain from 3 experiments. The data in (**F**) and (**G**) correspond to the mean ± SEM values. The *P* value in (**E**) was calculated using the absolute number of cells in each cluster via contingency table analysis (chi-square test). The *P* values in (**F**) and (**G**) were calculated via one-way ANOVA

Short-term intraperitoneal anti-CD3 mAb treatment triggers the expansion of a transcriptionally and phenotypically heterogeneous pool of IL-10^+^ FOXP3^–^ CD4^+^ T cells in the spleen and the intraepithelial and lamina propria compartments in the gut [2]. To ascertain a potential role of BCL6 in the formation of this T-cell pool, we performed scRNAseq of splenic CD25^–^ CD4^+^ T cells isolated from anti-CD3 mAb-treated NOD.*Il10-eGFP* (*Bcl6*^+/+^) and NOD.*Il10-eGFP*.*Cd4-Cre*.*Bcl6*^*loxP/loxP*^ mice. As expected [6], the scRNAseq profiles of the splenic eGFP^+^ cells (reporting IL-10 expression) from both types of mice were heterogeneous, containing three major clusters (Fig. 7A). However, clusters #0 and #1 from anti-CD3 mAb-treated NOD.*Il10-eGFP* (*Bcl6*^+/+^) mice were most closely related to Tconv cells, and cluster #2 cells were most closely related to the terminally differentiated TR1 cells induced by pMHCII-NPs, followed by TFH cells (Fig. 7B, C and Supplementary Fig. 14) (Datasheet 26). This does not imply that anti-CD3 mAb-induced clusters #0 and #1 represent Tconv cells. Rather, they most likely correspond to a heterogeneous pool of cells enriched for the transitional TR1-like subset described in pMHCII-NP-treated mice (*Maf*+, as is also the case for cluster #0 and #1 cells; Fig. 7B). This is consistent with the low overall degrees of *Bcl6* expression in all these eGFP^+^ anti-CD3 mAb-induced clusters, which are similar to those corresponding to the pMHCII-NP-induced Tet^+^ TR1-like/TR1 pool and substantially different from those seen in the pMHCII-NP-induced Tet^+^ TFH-like pool (Fig. 7C).

Notably, within the IL-10^+^ cell population from NOD.*Il10-eGFP*.*Cd4-Cre*.*Bcl6*^*loxP/loxP*^ mice, the size of TR1-related cluster #2 was significantly reduced at the expense of an increase in the size of Tconv-related cluster #0 (containing residual CD25^+^ (FOXP3^+^) Tregs and other IL-10-expressing cells) (Fig. 7D, E), consistent with a TFH origin for TR1 cells, whether induced by pMHCII-NPs or other means. To further substantiate these observations and ascertain a role for BLIMP1 in anti-CD3 mAb-induced TR1 cell formation, we compared the splenic CD4^+^ T cells from anti-CD3 mAb-treated NOD.*Cd4-Cre*, NOD.*Cd4-Cre*.*Bcl6*^*loxP/loxP*^ and NOD.*Cd4-Cre*.*Prdm1*^*loxP/loxP*^ mice via mass cytometry, as described above. Anti-CD3 mAb treatment triggered the formation of significantly fewer IL-10^+^ CD4^+^ T cells in both NOD.*Cd4-Cre*.*Bcl6*^*loxP/loxP*^ and NOD.*Cd4-Cre*.*Prdm1*^*loxP/loxP*^ mice (Fig. 7F) and significantly fewer TR1-like (CD49b^+^/LAG-3^+^; coinhibitory molecule-rich) cells within the FOXP3^–^IL-10^+^ CD4^+^ T-cell pool (Fig. 7G). Thus, anti-CD3 mAb-induced TR1 cell formation, similar to that induced by pMHCII-NPs, is both BCL6- and BLIMP1 dependent.

### The pMHCII-NP-induced tetramer^+^ TFH-like subpool and antigen-induced TFH cells express overlapping scMultiome profiles

**Fig. 8:**
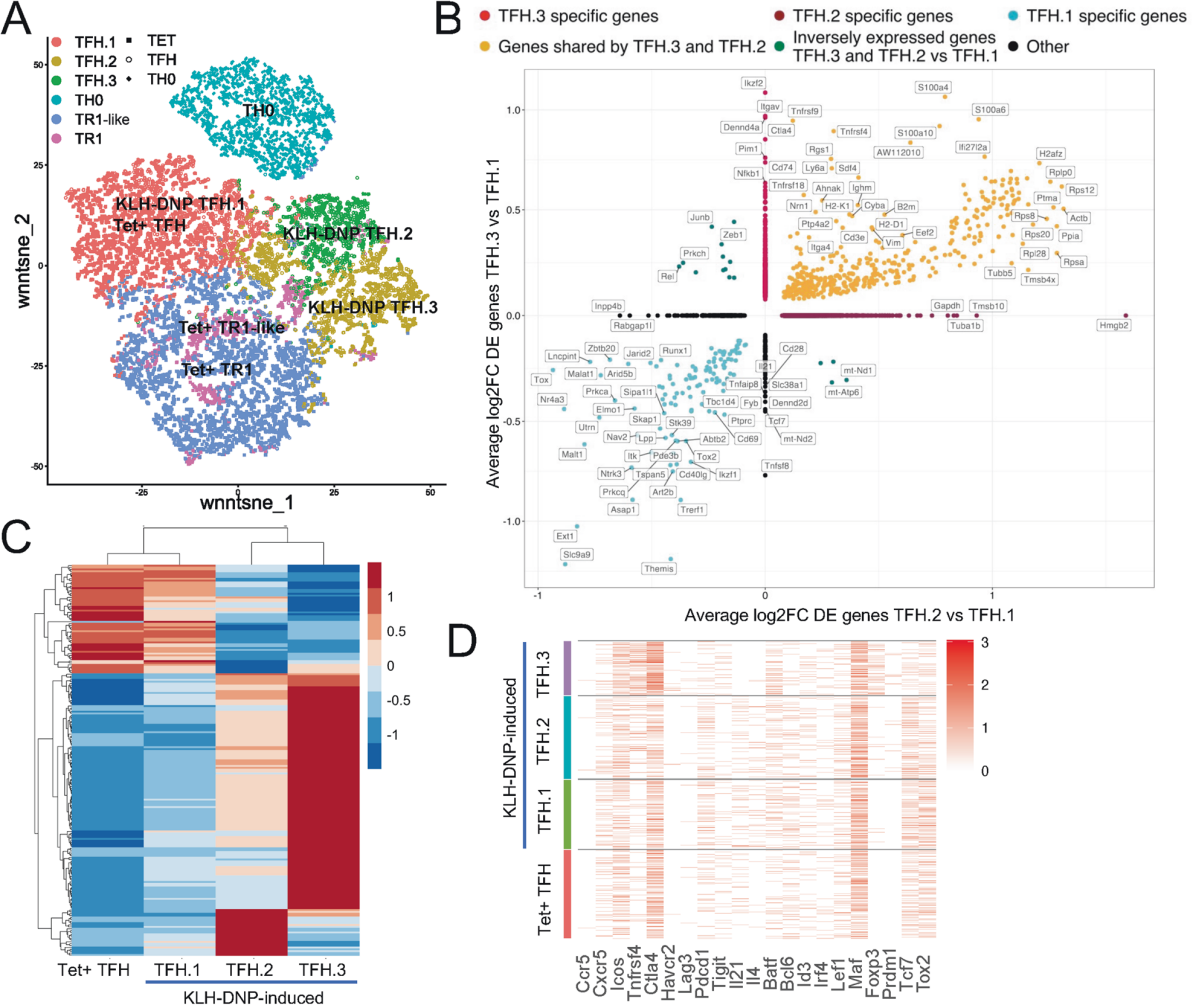
Single-cell multiomic analysis of pMHCII-NP-induced TFH-TR1 cells vs. KLH-induced TFH cells. **A** tSNE plot of weighted nearest neighbor-integrated scRNAseq and scATACseq data from BDC2.5 mi/IA^g7^ Tet^+^, KLH-induced TFH and TH0 cells. The colors represent the different K-means and their predicted identity. BDC2.5 mi/IA^g7^ Tet^+^ subclustered into TFH, TR1-like and TR1 cells, while KLH-induced cells subclustered into TFH.1, TFH.2 and TFH.3 cells. BDC2.5 mi/IA^g7^ Tet^+^ TFH and KLH-induced TFH.1 cells clustered together. The data are from 4 (BDC2.5 mi/IA^g7^ Tet^+^) and 8 mice (KLH-induced TFH and TH0) from 2 experiments. **B** Two-dimensional plot of the average log2FC values for the differentially expressed genes (adjusted *P* value <0.05) among KLH-DNP-induced TFH cell subtypes (TFH.1, TFH.2 and TFH.3) from (**A**). The data were obtained from 10X Genomics scRNAseq for CD4^+^CD44^hi^PD-1^hi^CXCR5^hi^ T cells sorted from KLH-DNP-immunized NOD mice (*n* = 5). The X-axis shows the TFH.2 vs. TFH.1 comparison; the Y-axis, the TFH.3 vs. TFH.1 comparison. The dot color represents the subset specificity of differential gene expression. **C** Heatmap and dendrogram showing the average relative gene expression levels in BDC2.5 mi/IA^g7^ Tet^+^ TFH cells and the three clusters within the KLH-induced TFH cell pool (TFH.1, TFH.2 and TFH.3). **D** Single-cell resolution heatmap showing the relative expression levels of TFH-related genes in BDC2.5 mi/IA^g7^ Tet^+^ TFH cells and the three clusters within the KLH-induced TFH pool (TFH.1, TFH.2 and TFH.3)

To further investigate whether the TFH-like subpools arising in pMHCII-NP-treated mice indeed correspond to bona fide TFH cells, we compared the scMultiome (simultaneous scATACseq/scRNAseq) profiles of tetramer^+^ cells arising in response to BDC2.5 mi/IA^g7^-NP therapy to those of the TFH-like cells (CD4^+^CD44^hi^PD-1^hi^CXCR5^hi^) arising in response to conventional immunization with the KLH-DNP conjugate. The KLH-DNP-induced TFH pool contained three subclusters, herein referred to as TFH.1, TFH.2 and TFH.3 cells (Fig. 8A). Figure 8B (Datasheets 27, 28) shows a comparison of the gene expression profiles of these three different TFH subclusters via a two-dimensional plot of the average log2FC values for the differentially expressed genes (adjusted *p* value < 0.05) to highlight genes that are subcluster-specific or shared. Remarkably, the scMultiome profile of the pMHCII-NP-induced TFH subpool overlapped with that of the subpool of KLH-DNP-induced TFH cells (herein referred to as TFH.1) that most closely resembled effector *Bcl6*^*hi*^*Tox2*^*hi*^*Il21*^*+*^*Pdcd1*^*+*^ TFH cells (Fig. 8A–D). These two subsets only had 5 differentially expressed genes (Log2FC > 0.5 or <−0.5). The antigen-induced TFH.2 cells were also similar to their TFH.1 counterparts but expressed lower levels of *Bcl6, Tox2, Il21* and *Pdcd1* and higher levels of *Maf, Tcf7, Cxcr5* and *Cd69* (Fig. 8B–D; Datasheets 27, 28). The antigen-induced TFH.3 cells displayed features of TFR cells (*Foxp3*^*+*^*Bcl6*^*+*^*Bhlhe40*^*+*^*Icos*^*+*^*Il10*^*+*^) (Fig. 8B–D; Datasheets 27, 28). The scMultiome profiles of the pMHCII-NP-induced TR1-like and TR1 cell pools overlapped with each other because although the cells in these pools express distinct scRNAseq profiles, they share nearly identical scATACseq profiles.

### pMHCII-NPs can induce cognate TR1 cell formation in TFH cell-transfused NOD.*Scid* hosts in a BLIMP1-dependent manner

Collectively, the above observations, including the observed shift in the TR1:TFH subcluster ratio as a function of dose number (Supplementary Fig. 11K), support the idea that expansion of the pMHCII-NP-cognate TFH cell pool precedes the differentiation of these cells into their TR1-like counterparts.

**Fig. 9:**
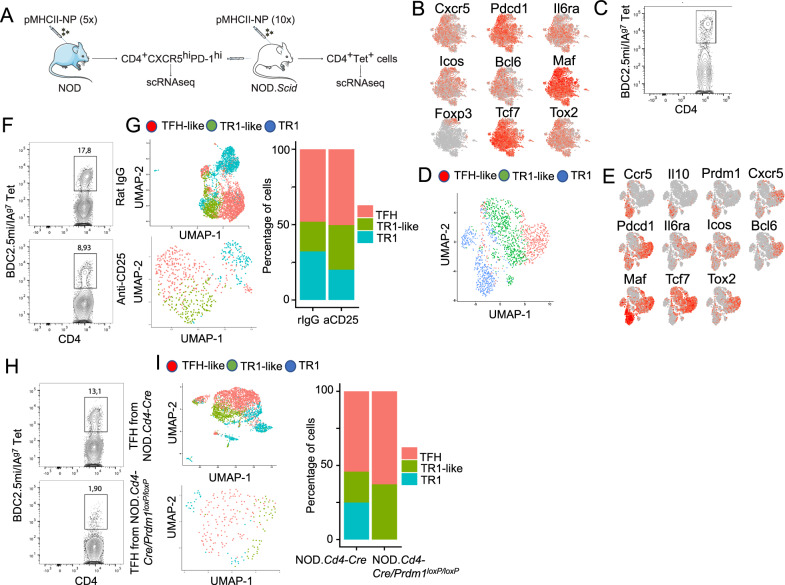
pMHCII-NP-induced formation of TR1-like and terminally differentiated TR1 cells from PD-1^hi^CXCR5^hi^ precursors. **A** Cartoon showing the experimental approach used to track the development of TR1 cells from PD-1^hi^CXCR5^hi^ precursors. Briefly, we transferred FACS-sorted PD-1^hi^CXCR5^hi^ CD4^+^ T cells from the spleens of female NOD mice treated with 5 doses of BDC2.5 mi/I-A^g7^-NPs (1.5 × 10^5^) into female NOD.*Scid* hosts and treated the hosts with 10 additional doses of pMHCII-NPs. **B** UMAP-based feature plots for representative TFH-associated gene transcripts (and *Foxp3*) corresponding to the PD-1^hi^CXCR5^hi^ CD4^+^ T cells used for transfer. **C** FACS profile of BDC2.5 mi/I-A^g7^ tetramer^+^ CD4^+^ cells arising in PD-1^hi^CXCR5^hi^ cell-transfused NOD.*Scid* hosts upon BDC2.5 mi/I-A^g7^-NP treatment (10 doses over 5 weeks). The data correspond to cells pooled from 2 hosts from 1 experiment. **D** UMAP plots showing the Tet^+^ TFH, TR1-like and TR1 subsets within the BDC2.5 mi/I-A^g7^ tetramer^+^ CD4^+^ cell pool from NOD.*Scid* hosts (**C**), after sorting and scRNAseq analysis. **E** Feature plots for representative TFH and TR1-associated gene transcripts in the UMAP dimensionality reduction analysis from (**D**). **F** FACS profiles for BDC2.5 mi/I-A^g7^ tetramer^+^ CD4^+^ cells arising in PD-1^hi^CXCR5^hi^ cell-transfused NOD.*Scid* hosts upon treatment with BDC2.5 mi/I-A^g7^-NP (10 doses over 5 weeks) and the anti-CD25 mAb or rat IgG (10 doses of 500 μg i.p). The data correspond to cells pooled from 2 hosts for each treatment group from 1 experiment. The value shown corresponds to the percentage of tetramer^+^ cells within the CD4^+^ gate. **G** scRNAseq profiles for the tetramer^+^ cells from (**F**). Left, UMAP plots; right, relative percentages of TFH, TR1-like and TR1 cells within the tetramer^+^ cell pool. **H** FACS profiles for BDC2.5 mi/I-A^g7^ tetramer^+^ CD4^+^ cells arising in NOD.*Scid* hosts transfused with PD-1^hi^CXCR5^hi^ cells from NOD.*Cd4-Cre* or NOD.*Cd4-Cre.Prdm1*^*loxP/loxP*^ mice upon treatment with BDC2.5 mi/I-A^g7^-NPs (10 doses over 5 weeks). The data correspond to cells pooled from 2 hosts for each treatment group from 1 experiment. The value shown corresponds to the percentage of tetramer^+^ cells within the CD4^+^ gate. **I** scRNAseq profiles for the tetramer^+^ cells from (**H**). Left, UMAP plots; right, relative percentages of TFH, TR1-like and TR1 cells within the tetramer^+^ cell pool

To further substantiate this interpretation, we transferred FACS-sorted PD-1^hi^CXCR5^hi^ CD4^+^ T cells from the spleens of NOD mice treated with 5 doses of BDC2.5 mi/I-A^g7^-NPs (1.5 × 10^5^; bulk TFH cells containing pMHCII-NP-induced TFH-like cells but lacking the CXCR5^−^ TR1 subpools) into NOD.*Scid* hosts and treated the hosts with 10 additional doses of pMHCII-NPs (Fig. 9A). scRNAseq analyses indicated that the PD-1^hi^CXCR5^hi^ CD4^+^ T-cell pool used for transfer (Fig. 9B and Supplementary Fig. 15A) was largely composed of TFH-like cells homogeneously expressing the CXCR5- and PD-1 coding transcripts as well as other TFH transcripts, such as *Bcl6, Il6ra, Icos, Maf, Tcf7* and *Tox2*, and a very small *Foxp3*^*+*^, TFR-like T-cell subset (Fig. 9B). In contrast, the BDC2.5 mi/I-A^g7^ tetramer^+^ CD4^+^ cells arising in the hosts (Fig. 9C) contained a small TFH-like pool (27%) and a larger TR1 pool containing both TR1-like cells (39%) and TR1 cells (34%; Fig. 9D, E) (Datasheet 29) (cell cluster assignment was performed using the scRNAseq data obtained for the pMHCII-NP-induced Tet^+^ TFH, TR1-like and TR1 cells described above). Thus, pMHCII-NP-induced TFH-like cells can be subsequently reprogrammed into TR1-like and TR1 progeny in a separate host.

In subsequent experiments, and in agreement with the data obtained in NOD.*Cd4-Cre*.*Il2*^*loxP/loxP*^ mice (Fig. 4G, H), TFH cell-transfused NOD.*Scid* hosts that received treatment with both pMHCII-NPs and a blocking anti-CD25 mAb contained smaller percentages (~half) of BDC2.5 mi/I-A^g7^ tetramer^+^ CD4^+^ cells and TR1 subcluster cells than their pMHCII-NP/rat IgG-treated control counterpart hosts (Fig. 9F, G and Supplementary Fig. 16A).

We next carried out similar adoptive TFH cell transfer experiments using TFH cells from NOD.*Cd4-Cre*.*Prdm1*^*loxP/loxP*^ or NOD.*Cd4-Cre* donors to confirm that terminal differentiation of TR1 cells requires BLIMP1. The BDC2.5 mi/I-A^g7^-NP-induced tetramer^+^ CD4^+^ cell pool arising in NOD.*Scid* mice transfused with TFH cells from NOD.*Cd4-Cre*.*Prdm1*^*loxP/loxP*^ mice was significantly smaller (~7-fold) than that arising in NOD.*Scid* mice transfused with TFH cells from NOD.*Cd4-Cre* mice (Fig. 9H and Supplementary Fig. 16B), and this cell pool exclusively contained TFH-like and TR1-like cells but no terminally differentiated TR1 cells (Fig. 9I and Supplementary Fig. 16C). Of note, the TFH population obtained from donor NOD.*Cd4-Cre*.*Prdm1*^*loxP/loxP*^ mice contained an enlarged pool of TFR-like *Foxp3*^+^ cells compared to that isolated from wild-type NOD mice (Supplementary Fig. 16C). Thus, as suggested by the studies in pMHCII-NP-treated NOD.*Cd4-Cre*.*Prdm1*^*loxP/loxP*^ mice, the TFH-TR1 cell transdifferentiation process, unlike the formation and homeostasis of FOXP3^+^ Treg and TFR cells, is BLIMP1 dependent.

### pMHCII-NP-induced TFH and TR1 CD4^+^ T cells have distinct functional properties

**Fig. 10:**
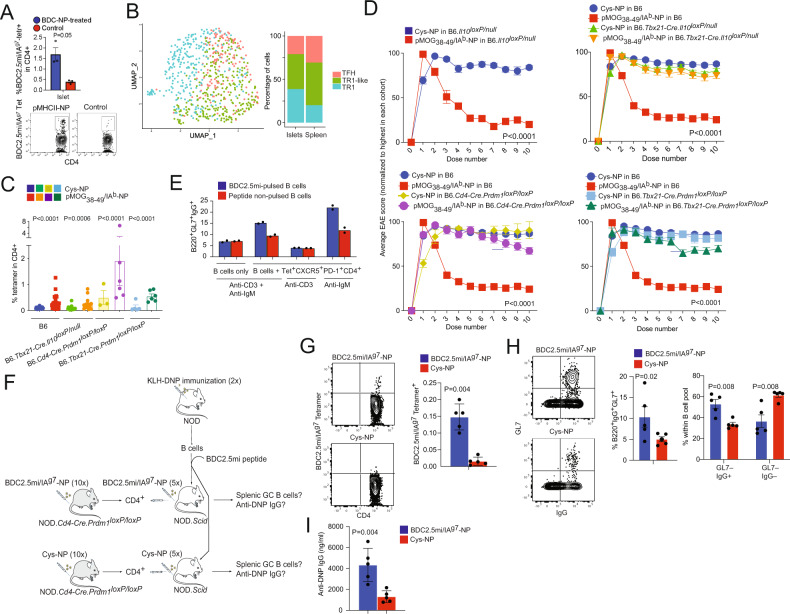
Functional properties of TFH and TR1 cells arising in response to pMHCII-NP therapy. **A** Top, Average percentages of tetramer^+^ CD4^+^ T cells within the islet-associated CD4^+^ T-cell pool of BDC2.5 mi/IA^g7^-NP- vs. control NP-treated NOD mice. Bottom, representative tetramer staining profiles. The data correspond to *n* = 3 samples per treatment type from 5 to 10 mice each from one experiment. **B** Left: UMAP scRNAseq plots for the islet-associated tetramer^+^ CD4^+^ T cells from (**A**). Right: Relative distribution of TFH, TR1-like and TR1 subpools within the islet- and spleen-associated tetramer^+^ cell pools of BDC2.5 mi/IA^g7^-NP-treated NOD mice. **C** Percentages of splenic pMOG_38-49_/I-A^b^ Tet^+^ cells in B6, B6.*Tbx21-Cre*.*Il10*^*loxP/loxP*^, *B6.Cd4-Cre*.*Prdm1*^*loxP/loxP*^ and B6.*Tbx21-Cre*.*Prdm1*^*loxP/loxP*^ mice (both males and females) upon treatment with pMOG_38-49_/I-A^b^-NPs (*n* = 33 (B6), 17 (B6.*Tbx21-Cre*.*Il10*^*loxP/loxP*^), 6 (*B6.Cd4-Cre*.*Prdm1*^*loxP/loxP*^) and 6 (B6.*Tbx21-Cre*.*Prdm1*^*loxP/loxP*^)) or Cys-NPs (control; *n* = 23, 17, 3 and 5, respectively) (10 doses over 5 weeks, starting when the EAE score was >1.5/5). The data are from 6, 4, 1 and 1 experiments, respectively. **D** Top left: normalized EAE scores in B6 vs. Cre-negative B6.*Il10*^*loxP/loxP*^ mice upon pMOG_38-49_/I-A^b^-NP or Cys-NP treatment (*n* = 12 (B6) and 13 (Cre-negative B6.*Il10*^*loxP/loxP*^), respectively, from 2 experiments). Top right: normalized EAE scores in B6 vs. B6.*Tbx21-Cre*.*Il10*^*loxP/loxP*^ mice upon treatment with pMOG_38-49_/I-A^b^-NPs (*n* = 40 and 22, respectively) or Cys-NPs (control; n = 28 and 19, respectively; from 6 and 4 experiments, respectively). Bottom left: normalized EAE scores in B6 vs. *B6.Cd4-Cre*.*Prdm1*^*loxP/loxP*^ mice upon treatment with pMOG_38-49_/I-A^b^-NPs (n = 40 and 6, respectively) or Cys-NPs (control; *n* = 28 and 4; from 6 and 1 experiments, respectively). Bottom right: normalized EAE scores in B6 vs. B6.*Tbx21-Cre*.*Prdm1*^*loxP/loxP*^ mice upon treatment with pMOG_38-49_/I-A^b^-NPs (*n* = 40 and 6, respectively) or Cys-NPs (control; *n* = 28 and 6; from 6 and 1 experiments, respectively). **E** Percentages of GL7^+^IgG^+^ cells in cultures of purified Tet^+^ PD-1^hi^CXCR5^hi^ CD4^+^ T cells from BDC2.5 mi/I-A^g7^-NP-treated NOD mice (10 doses over 5 weeks) (3 × 10^4^ cells/well) with BDC2.5 mi peptide-pulsed or nonpulsed B cells (5 × 10^4^ cells/condition/well) isolated from the draining lymph nodes of KLH-DNP-immunized NOD mice in the presence of an anti-CD3 (2 μg/mL) and/or anti-IgM (5 μg/mL) antibody for 6 days. The data correspond to two samples per condition from one experiment. **F** Cartoon showing the experimental approach used to measure the ability of pMHCII-NP-expanded TFH-like cells to promote GC B-cell formation and antibody production. Briefly, we first treated 10 NOD.*Cd4-Cre*.*Prdm1*^*loxP/loxP*^ mice with 10 doses of Cys-NPs or BDC2.5 mi/I-A^g7^-NPs (*n* = 5 mice each). The total splenic CD4^+^ T cells from each donor were then transferred into NOD.*Scid* hosts (1.6 × 10^7^/host), and the hosts were treated with 5 additional doses of Cys-NPs or BDC2.5 mi/I-A^g7^-NPs. At this point, all the hosts were transfused with BDC2.5 mi peptide-pulsed splenic B cells isolated from NOD mice immunized with KLH-DNP (10^7^ cells/host). Seven days after B-cell transfer, the hosts were killed, their spleens were analyzed for the presence of Tet^+^ CD4^+^ T cells, GC B cells (GL7^+^sIgG^+^) and non-GC B cells (GL7^−^sIgG^+^ or GL7^−^sIgG^−^) within the B220^+^ cell pool^,^ and their serum was analyzed for the presence of anti-DNP antibodies. **G** Left: Representative BDC2.5/I-A^g7^ tetramer and CD4 staining profiles from the pMHCII-NP- and Cys-NP-treated NOD.*Scid* hosts from (**F**). Right: Average percentages of BDC2.5/I-A^g7^ Tet^+^ CD4^+^ T cells in the hosts from (**F**). The data correspond to *n* = 5 female mice per group from one experiment. **H** Left: Representative GL7 and IgG staining profiles for splenic B cells from the NOD.*Scid* hosts from (**F**). Right: percentages of GL7^+^sIgG^+^, GL7^−^sIgG^+^ and GL7^−^sIgG^−^ cells among the splenic B cells of the NOD.*Scid* hosts from (**F**). The data correspond to *n* = 5 female mice per group from one experiment. **I** Serum anti-DNP antibody levels in the NOD.*Scid* hosts from (**D**). The data correspond to *n* = 5 female mice per group from one experiment. The data in (**C**–**E**) and (**G**–**I**) correspond to the mean ± SEM values. The *P* values in (**C**, **G**–**I**) were calculated via the Mann‒Whitney U test. The *P* values in (**D**) were calculated via two-way ANOVA

We previously showed that in vitro, the tetramer^+^ CD4^+^ T cells of BDC2.5 mi/I-A^g7^-NP-treated mice suppressed the proliferation of noncognate (pMHC class I-specific) CD8^+^ T cells in response to peptide-pulsed dendritic cells (DCs) in an IL-10- and TGFβ-dependent manner [11]. Likewise, the bulk splenic CD4^+^ T cells and the tetramer^+^ CD4^+^ T cells from BDC2.5 mi/I-A^g7^-NP-treated donors (but not the splenic CD4^+^ T cells from untreated donors or the tetramer^–^ cells from pMHCII-NP-treated mice) efficiently suppressed diabetes development in T-cell-reconstituted NOD.*Scid* hosts [11]. Furthermore, this effect was potentiated by further treatment of the NOD.*Scid* hosts with BDC2.5 mi/I-A^g7^-NPs or by cotransfer of pancreatic lymph node (PLN) but not mesenteric lymph node (MLN) B cells from pMHCII-NP-treated mice (containing or lacking TR1-induced Breg cells, respectively). Collectively, these data demonstrate that the tetramer^+^ CD4^+^ T-cell pools arising in vivo in response to BDC2.5 mi/I-A^g7^-NPs have dominant immunoregulatory properties both ex vivo and in vivo and that the latter are enhanced by the cotransfer of TR1 cell-induced, PLN-associated Breg cells, indicating synergistic effects [11]. Interestingly, and as expected based on the distinct chemokine receptor profiles of the Tet^+^ TFH-like and TR1-like/TR1 subclusters (Supplementary Fig. 8A, B), the islet-associated CD4^+^ T cells from BDC2.5 mi/IA^g7^-NP-treated mice harbored significantly increased percentages of BDC2.5 mi/IA^g7^ tetramer^+^ T cells compared with those from mice treated with control NPs (Fig. 10A). Furthermore, scRNAseq studies of the islet-associated tetramer^+^ cells from BDC2.5 mi/IA^g7^-NP-treated mice demonstrated that these cells were enriched for the TR1 subcluster found in the splenic tetramer^+^ T-cell pool at the expense of its TR1-like and TFH cell counterparts, consistent with increased tropism for sites of inflammation (Fig. 10B and Supplementary Fig. 17).

Given the presence of TFH-like cells within the pMHCII-NP-induced tetramer^+^ T-cell pool, we sought to investigate whether they possessed immunoregulatory and/or TFH cell-like functional properties. We previously showed that pMOG_38-49_/I-A^b^-coated NPs can reverse established pMOG_35-55_-induced experimental autoimmune encephalomyelitis (EAE) by triggering the formation and expansion of cognate TR1-like cells and the activation of downstream immunoregulatory networks similar to those seen in other disease models [11]. Since expression of *Il10* occurs specifically at the TR1 cell stage and IL-10 is necessary for therapeutic activity [11], we investigated whether pMOG_38-49_/I-A^b^-NPs can reverse limb and tail paralysis in pMOG_35-55_-immunized B6.*Tbx21-Cre*.*Il10*^*loxP/mut*^ mice (to delete *Il10* at the T-BET-expressing TR1-like and TR1 cell stages; Fig. 6E) compared to B6.*Il10*^*loxP/mut*^ control and wild-type B6 mice. Whereas pMOG_38-49_/IA^b^-NP therapy reversed limb paralysis in Cre– B6.*Il10*^*loxP*/^^*mut*^ mice in association with a significant expansion of cognate Tet^+^ cells, it had virtually no therapeutic activity in B6.*Tbx21-Cre.Il10*^*loxP*/^^*mut*^ mice, despite having adequate pharmacodynamic activity in these animals (Fig. 10C, D; Supplementary Fig. 15B). Similar outcomes were observed when these experiments were performed in mice lacking *Prdm1* expression in all T cells or specifically in TR1-like/TR1 cells (*B6.Cd4-Cre*.*Prdm1*^*loxP/loxP*^ and B6.*Tbx21-Cre*.*Prdm1*^*loxP/loxP*^ mice, respectively; Fig. 10C, D). Thus, pMHCII-NPs lack therapeutic activity in mice in which the pMHCII-NP-induced tetramer^+^ TR1 cells cannot produce IL-10 or in which the pMHCII-NP-induced tetramer^+^ CD4^+^ T cells lack the BLIMP1 dependent, terminally differentiated IL-10-producing TR1 subpool (Fig. 6A).

These observations also raised the question of whether the TFH cells within the pMHCII-NP-induced Tet^+^ cell pools have TFH-like functional properties. This possibility was first investigated in vitro. This was done by coculturing purified Tet^+^ PD-1^hi^CXCR5^hi^ CD4^+^ T cells (to isolate the TFH-like subpool) from BDC2.5 mi/I-A^g7^-NP-treated (10 doses over 5 weeks) NOD mice (3×10^4^ cells/well) with BDC2.5 mi peptide-pulsed or unpulsed B cells (5 × 10^4^ cells/condition/well) isolated from the draining lymph nodes of KLH-DNP-immunized NOD mice in the presence of an anti-CD3 or anti-IgM antibody for 6 days [35]. The cultures containing peptide-pulsed B cells contained significantly higher percentages of GL7^+^sIgG^+^ B cells than those containing nonpulsed B cells (Fig. 10E and Supplementary Fig. 15C), suggesting that a cognate interaction between the therapy-induced Tet^+^ TFH-like cells and B cells can promote the formation of GC B cells.

We further investigated this possibility in vivo (Fig. 10F). We first treated 10 NOD.*Cd4-Cre*.*Prdm1*^*loxP/loxP*^ mice (unable to generate terminally differentiated Tet^+^ TR1 cells; Fig. 6A) with 10 doses of control NPs or BDC2.5 mi/I-A^g7^-NPs (*n* = 5 each); the latter treatment was performed to generate and expand BDC2.5 mi/I-A^g7^-specific CD4^+^ T-cell pools devoid of IL-10-producing Tet^+^ TR1 cells. The total splenic CD4^+^ T cells from each donor were then transferred into NOD.*Scid* hosts (1.6 × 10^7^ cells/host), and the hosts were treated with 5 additional doses of control NPs or BDC2.5 mi/I-A^g7^-NPs (the treatment received by each host was the same as that received by the donor mouse). At this point, all the hosts were transfused with BDC2.5 mi peptide-pulsed splenic B cells (10^7^ cells/host) isolated from NOD mice immunized with KLH-DNP [11]. This was done to (1) promote cognate recognition of the transfused B cells by BDC2.5 mi/I-A^g7^-NP-induced TFH-like cells and (2) skew the transferred B-cell pool toward anti-DNP antibody reactivity. Seven days after B-cell transfer, the hosts were killed; their spleens were analyzed for the presence of Tet^+^ CD4^+^ T cells (Supplementary Fig. 15D), GC B cells (GL7^+^sIgG^+^) and non-GC B cells (GL7^–^sIgG^+^ or GL7^–^sIgG^–^) within the B220^+^ cell pool (Supplementary Fig. 15E); and their serum was analyzed for the presence of anti-DNP antibodies. In mice receiving CD4^+^ T cells from control NP-treated donors, there were no BDC2.5/I-A^g7^ Tet^+^ CD4^+^ T cells, as expected (Fig. 10G), and their splenic B-cell population comprised ~5% GL7^+^sIgG^+^, ~30% GL7^−^sIgG^+^ and ~65% GL7^−^sIgG^−^ cells (Fig. 10H). In contrast, the spleens of the mice that received CD4^+^ T cells from pMHCII-NP-treated donors contained ~0.2% BDC2.5 mi/I-A^g7^ Tet^+^ cells (Fig. 10G) and approximately twice as many GL7^+^sIgG^+^ (~10%) and GL7^–^sIgG^+^ (~60%) cells and approximately half the GL7^−^sIgG^−^ B cells seen in mice receiving CD4^+^ T cells from control NP-treated donors (Fig. 10H). Importantly, this was associated with significantly increased anti-DNP antibody seropositivity (Fig. 10I). Collectively, these data demonstrate that the Tet^+^ CD4^+^ T-cell pools arising in response to BDC2.5 mi/I-A^g7^-NP treatment but not those isolated from control NP-treated mice contain T cells capable of eliciting GC B-cell formation, B-cell maturation and antibody secretion, consistent with the TFH-like transcriptome and phenotype of the Tet^+^ cells arising in the donor mice.

## Discussion

pMHCII-NPs trigger the sustained assembly of large TCR microclusters on cognate effector/memory T cells and a prolonged form of costimulation-independent TCR signaling that culminates in the reprogramming of an unknown subset of antigen-experienced CD4^+^ T cells into TR1-like cells [10, 11]. This is accompanied by systemic expansion of these cells, their recruitment to target organs, and suppression of local inflammation and reversal of several different experimental and spontaneous autoimmune disorders [10–13, 36]. These effects are exquisitely specific, sparing the ability of the immune system to mount immune responses to nominal antigen vaccines or to clear viral and bacterial infections or metastatic allogeneic tumors [11, 13]. Here, we demonstrate that these pMHCII-NP-induced TR1 cells, as well as their anti-CD3-induced counterparts, arise from cognate TFH cells and do so in a BLIMP1-dependent manner. Although these data do not exclude the ability of other T-cell types (e.g., TH17 cells) to transdifferentiate into TR1-like cells in response to other cues [37], they expose the TFH-TR1 axis as a new target for therapeutic intervention in immunity and autoimmunity.

Roncarolo et al. [3] phenotyped the human peripheral blood FOXP3-negative IL-10-producing CD4^+^ Treg cell subset and its murine counterpart to propose coexpression of the integrin CD49b and the coinhibitory molecule LAG-3 as specific markers for the identification and isolation of both human and murine TR1 cells [2]. By focusing on anti-CD3 mAb-induced FOXP3^–^ IL-10-producing CD4^+^ T cells, Gagliani et al. [2] subsequently showed that this population of cells is transcriptionally and phenotypically heterogeneous and defined the true regulatory subset as being coinhibitory receptor (CIR)-rich [6]. These authors further suggested that, in addition to CD49b and LAG-3, PD-1 and CCR5 can also be used to identify these IL-10-producing cells within the CD4^+^ population [6]. On the other hand, studies of IL-10-producing TR1-like cells generated from human PBMCs in vitro upon stimulation through several strategies, including antigen-presenting cells (e.g., dendritic cells –DCs–), cytokines (IL-10, IL-21, IL-27), costimulatory molecules (CD46, CD55), vitamin D3 and immunosuppressive drugs, have suggested that none of these coinhibitory receptors are consistently expressed in the different TR1-like cell subsets that were obtained [7]. In contrast, we showed here that pMHCII-NPs trigger the formation of transcriptionally homogeneous pools of profoundly immunoregulatory and poly/oligoclonal TR1-like cells that coexpress many of the markers previously ascribed to different subsets of TR1-like cells, as opposed to a mixture of cells at different stages of development and with variable regulatory potential.

This list includes the genes coding for the coinhibitory receptors LAG-3, CTLA-4, PD-1 and TIGIT; the costimulators ICOS and OX40; the cytokines IL-10, IL-21 and IFNγ; and the chemokine receptor CXCR3. IL-10 is directly responsible for some of the immunoregulatory properties of TR1 cells. IL-21 plays key roles in sustaining IL-10 expression in TR1 cells, in the homeostatic regulation of this T-cell subset and in TR1-induced Breg cell formation [11]. This list of TR1-upregulated loci in pMHCII-NP-induced TR1 cells also includes those encoding several key TFs (*Tbx21, Maf, Prdm1, Egr2, Irf4, Nfil3*, and *Ahr*) and the gene encoding the transcriptional coactivator OCAB (*Pou2fa1*), all previously implicated in IL-10 expression [38, 39]. Other TF genes upregulated in pMHCII-NP-induced cells include *Bhlhe40*, *Runx2* and *Vdr*, which are implicated in the development, maintenance or function of IL-10-expressing Treg cells.

The requirement for IFNγ [11], the expression of the TH1 TF T-BET, the c-MAF, IL-10 and IL-21 expression competency of effector and memory CD4^+^ T cells [38], and the ability of pMHCII-NPs to convert ‘TR1-poised’ T cells primed by active immunization into TR1-like cells suggested that these TR1 precursors might be effector/memory TH1 cells. Although other studies have suggested this to be the case, a detailed review of the data does not always support this contention. For example, in a chronic *Plasmodium* infection model, Lönnberg et al. claimed a TH1 origin for TR1 cells based solely on the presence of IL-10-expressing cells within the infection-induced TH1 pool and on the assumption that TR1 cells are simply IL-10/IFNγ-coexpressing cells [40]. In fact, the IL-10^+^ and IL-10^−^ TH1 cells of these mice had only two differentially expressed genes (*Trib2* and *BC017643*), and the authors did not provide any other evidence for the TR1-like nature of their IL-10^+^ cells. A later study by Soon et al. also in a chronic *Plasmodium* infection model, reported a similar finding; 34% of TH1 lineage cells coexpressed *Ifng* and *Il10* [41]. Thus, based on the evidence provided, it is likely that the IL-10^+^ cells that arose in these mice were IL-10-expressing TH1 cells rather than bona fide TR1 cells. While it remains possible that TH1 cells can also give rise to TR1-like cells, IL-10 expression alone cannot be used to define the TR1ness of cells.

Surprisingly, we found here that the cognate Tet^+^ cells expanding in response to pMHCII-NPs express a transcriptional program that shares considerable features with TFH cells, including downregulation of the TFH-suppressing TF *Foxp1, S1pr1* and *Bach2* and downregulation of the T-cell zone retention receptor genes *Ccr7* and *Selplg*, as well as coexpression and/or upregulation of the TFH-associated TF-coding genes *Ascl2*, *Batf*, *Bcl6*, *Cebpa*, *Irf4*, *Maf*, *Stat1*, *Stat3*, *Tcf7*, *Tox2*, and *Vdr*; the B-cell zone homing chemokine receptor genes *Cxcr5* and *Cxcr3*; the germinal center retention adhesion molecule-coding gene *S1pr2*; the costimulatory genes *Icos* and *Sh2d1a*; the coinhibitory receptor genes *Ctla4*, *Lag3*, *Pdcd1* and *Tigit*; and the cytokine gene *Il21*. This similarity suggested that these cells might arise from TFH precursors.

However, there also appeared to be some significant differences between these two T-cell types. The pMHCII-NP-induced Tet^+^ CD4^+^ T-cell subset exhibited downregulation of *Lef1* and upregulation of both *Bcl6* and *Prdm1* compared to Tconv cells. In contrast, TFH cells exhibited upregulation of *Lef1*, which promotes *Bcl6* expression (a master regulator of TFH cell formation) but suppresses *Prdm1* expression (a repressor of TFH formation) [17, 32]. Furthermore, whereas TOX2 promotes BCL6 expression and suppresses T-BET expression in TFH cells [42], the Tet^+^ CD4^+^ T cells exhibited coupregulation of the genes coding for all three of the abovementioned TFs (TOX2, BCL6 and T-BET).

These differences in TF gene expression raised the possibility that the pMHCII-NP-expanded Tet^+^ pool might contain transitional Tet^+^ TFH-like cells. Indeed, scRNAseq and mass cytometry analyses demonstrated the presence of a significant Tet^+^ TFH-like cluster that was separated from its TR1-like counterpart, further supporting a TFH cell origin for pMHCII-NP-induced TR1 cells. Thus, when compared to each other, these two major clusters of tetramer^+^ cells remained markedly similar, but the TR1 subpool had significantly downregulated expression of key TFH-specific genes, including *S1pr2*, *Cxcr4*, *Cxcr5*, *Pdcd1*, *Il4*, *Ascl2*, *Bcl6*, *Cba2t3*, *Cebpa*, *Id3*, *Nfia*, *Pou2af1*, and *Tox2*, but upregulation of TR1-associated genes, such as *Ccr5*, *Havcr2*, *Il10*, *Ahr*, *Myc* and *Prdm1*. Clearly, these two clusters were developmentally related because they harbored identical clonotypes.

Another potential line of evidence suggesting that murine TR1-like cells might arise from TFH precursors lies in the roles of IL-27 in the formation of TFH vs. TR1-like cells and the ability of both subsets to productively interact and reprogram B cells, albeit in different ways. Although IL-27 is not necessary for the differentiation of TFH cells, it can trigger the differentiation of T cells into BCL6^+^CXCR5^+^PD-1^hi^Foxp1^lo^ TFH cells and promote IL-21 expression as well as TFH cell survival and homeostasis [43]. Interestingly, IL-27 has also been shown to play a role in the induction of TR1-like cells in vitro [38]. IL-27, however, is dispensable for the pMHCII-NP-mediated induction of TR1-like cell differentiation from antigen-experienced T cells in vivo [11]. These observations suggest that IL-27 and pMHCII-NPs lie upstream and downstream of TFH cell formation, respectively; IL-27 can elicit the formation of both TFH and TR1 cells from naïve precursors, whereas pMHCII-NPs can only promote the conversion of TFH cells into TR1-like cells. Another line of evidence that provided a compelling link between TFH cells and pMHCII-NP-induced TR1 cells is the ability of both T-cell types to productively engage with B cells: TFH cells as drivers of the GC reaction leading to the generation of high-affinity memory B cells and TR1 cells as drivers of IL-10-producing Breg cell formation [11, 13]. IL-21, upregulated in both TFH and TR1 cells, plays a critical role in both cases, but the outcome is completely different, suggesting that, as is the case for TFs, the effects of this cytokine on B-cell phenotype and function are context dependent.

The suspected TFH origin of pMHCII-NP-induced TR1 cells was further supported by two additional lines of evidence. First, treatment of NOD.*Scid* mice engrafted with total CXCR5^hi^PD-1^hi^ CD4^+^ T cells (containing pMHCII-NP-expanded TFH-like cells but devoid of terminally differentiated Tet^+^ TR1 cells) with pMHCII-NPs led to the formation of cognate TR1-like and terminally differentiated TR1 cell pools in the hosts. Second, the two-dimensional scMultiome profile of Tet^+^ TFH-like cells arising in response to BDC2.5 mi/IA^g7^-NP therapy was essentially identical to that corresponding to an effector TFH-like subpool of TFH-like cells (CD4^+^CD44^hi^CXCR5^hi^PD-1^hi^) induced by immunization with the KLH-DNP conjugate (*Bcl6*^*hi*^*Tox2*^*hi*^*Il21*^*+*^*Pdcd1*^*+*^; referred to as TFH.1 cells). The two other KLH-DNP-induced TFH subpools (referred to as TFH.3 and TFH.2 cells), which were not found within the pMHCII-NP-induced Tet^+^ T-cell pools, resembled TFR and circulating memory TFH cells, respectively. Although TFH cells are predominantly localized in GCs, where they interact with B cells and promote antibody production, the circulating lymphocyte population contains memory BCL6^low^ CD4^+^ T cells with TFH-like phenotypic and functional properties [44]. It has recently been shown that GC TFH cells exit the lymph node through the thoracic duct into the bloodstream [45] with downregulation of BCL6 [44]. Although the data suggest that cognate pMHCII-NPs trigger TR1 cell formation from GC TFH cells, we cannot exclude the possibility that they can also have this effect on the circulating TFH cell pool. It is worth noting that the size of the latter pool is significantly increased in a number of autoimmune diseases, including T1D [46].

TFH cell generation requires the expression of the transcriptional repressor BCL6, whose expression is induced by ICOS-mediated signals delivered by DCs at the T-cell zone [14–16]. By examining the pharmacodynamic activity of pMHCII-NPs and the anti-CD3 mAb in NOD mice expressing a conditional loxP-flanked *Bcl6* locus and a transgenic CD4 promoter-driven Cre recombinase to delete *Bcl6* in all T cells and render the mice TFH cell-deficient, we demonstrated that TR1 cell formation in response to these agents, at least in mice, requires the presence of TFH cells. In agreement with these data, T-cell-specific deletion of *Irf4*, which is necessary for TFH cell differentiation and GC formation, also abrogated pMHCII-NP-induced TFH/TR1 cell expansion and formation. *Il2*, which is involved in naïve T-cell proliferation and T-helper cell specification, also appears to play a role in the TFH-to-TR1 cell transdifferentiation process; IL-2 signaling inhibits the formation of endogenous TFH cells (prior to pMHCII-NP therapy) but promotes the expansion of cognate TFH cells in response to pMHCII-NP therapy and their subsequent transdifferentiation into TR1 cells. In contrast, deletion of *Tbx21*, required for TH1 cell differentiation [34], did not compromise these processes. These findings are compatible with our previous observations in NOD.*Ifng*^–/–^ mice, where pMHCII-NP therapy triggered the expansion of Tet^+^ cells with an altered cytokine profile (coexpression of both IL-10 and IL-4), consistent with IFN-γ-mediated suppression of IL-4 expression [11]. In agreement with this, the pMHCII-NP-induced Tet^+^ cells of NOD.*Cd4-Cre. Tbx21*^*loxP/loxP*^ mice did not express IFNγ and exhibited upregulation of IL-4 compared to the Tet^+^ cells of control mice.

Most importantly, studies in NOD mice expressing a conditional loxP-flanked *Prdm1* locus and a transgenic CD4 promoter-driven Cre recombinase for global deletion of *Prdm1* in T cells revealed that the TFH-to-TR1 cell conversion evolves toward a full-fledged TR1 subset (TR1) through a transitional (TR1-like) subset. This process involves progressive downregulation of TFH-related genes, such as *Bcl6* and *Cxcr5*, and upregulation of key TR1-related genes, such as *Prdm1*, *Il10* and *Ccr5*. Notably, abrogation of *Prdm1* expression completely blunted the TR1-like-to-TR1 conversion, indicating that this process requires the expression of BLIMP1, a zinc finger motif-containing transcriptional repressor that antagonizes BCL6 expression and function in both B- and T cells, including TFH cells [15, 17, 18, 33]. Thus, whereas deletion of *Bcl6* or *Irf4* in T cells precluded TFH formation and thus pMHCII-NP-induced cognate CD4^+^ T-cell expansion and downstream TR1 cell generation, deletion of *Prdm1* enabled the expansion and systemic accumulation of cognate TFH cells upon pMHCII-NP treatment while abrogating the conversion of these cells into TR1 progeny. This was accompanied by ~3- and ~4-fold increases in the frequencies of peripheral FOXP3^+^CD25^+^ Treg cells and TFH cells, respectively. This implies that BLIMP1 has opposite effects on FOXP3^+^ Treg and TFH cell formation vs. TR1 cell formation (i.e., suppressing vs. promoting this process) and that BCL6 and TFH cells lie upstream of BLIMP1 and TR1 cells in the process of pMHCII-NP-induced TR1-like cell formation. Since the expression of BLIMP1 in T cells is restricted to activated T cells and is induced by TCR ligation [47], and since pMHCII-NP therapy triggers the formation and expansion of cognate, antigen-specific TR1-like cell pools via sustained TCR signaling [10], we suspect that BLIMP1 expression in TFH cells is induced by repetitive encounters of cognate TFH cells with these compounds. Downregulation of BCL6 and upregulation of BLIMP1 by the transitional TR1-like cells might be facilitated by the loss of LEF1 in their TFH-like cell precursors; LEF1 is a positive and negative regulator of BCL6 and BLIMP1 expression, respectively. Regardless of the mechanistic underpinnings of BLIMP1 upregulation, Tet^+^ TR1-like and terminally differentiated TR1 cells (BLIMP1-independent and BLIMP1-dependent, respectively) differed in the expression levels of a substantial number of genes whose expression has been previously associated with this TF, including *Il10, Ctla4, Lag3, Icos, Havcr2, Tnfrsf4* and *Tnfrsf18*, among others [48].

This ability of BLIMP1 to promote the differentiation of antigen-specific TFH cells into TR1 cells may indicate the presence of a negative feedback regulatory loop designed to terminate TFH cell-induced immune responses during chronic infection/persistent antigenic stimulation in vivo. This possibility is supported by several observations. First, BLIMP1 has been implicated in the expression of IL-10 by antigen-specific CD4^+^ T cells during chronic lymphocytic choriomeningitis virus [49] and *Plasmodium* infections [50]. Second, BLIMP1 was found to be responsible for the appearance of ‘exhausted-like’ CD4^+^ T-cell subsets expressing TR1 markers such as LAG-3, CTLA-4, PD-1 and TIM3 under certain T-cell stimulation conditions [51, 52] as well as during chronic toxoplasmosis [53]. Third, BLIMP1 was implicated in the expression of IL-10 by an ill-defined ‘T-BET^+^’ Treg cell subset in a mouse model of influenza infection [54]. All these observations are compatible with BLIMP1-induced formation of TR1 cells from TFH precursors.

The above observations raise two additional questions: 1) do Tet^+^ TFH-like cells possess TFH-like functional properties? and 2) which of the three Tet^+^ subclusters contribute to the anti-inflammatory properties of the pMHCII-NP-induced Tet^+^ cell pools? The former question was addressed via both in vitro and in vivo experimentation. In vitro, purified Tet^+^ PD-1^hi^CXCR5^hi^ CD4^+^ T cells promoted the differentiation of cognate peptide-pulsed but not unpulsed B cells into GC B cells. In vivo in NOD.*Scid* hosts, total CD4^+^ T cells from pMHCII-NP-treated NOD.*Cd4-Cre*.*Prdm1*^*loxP/loxP*^ donors (containing Tet^+^ TFH and TR1-like cells but lacking terminally differentiated Tet^+^ TR1 cells) triggered both the formation of GC B cells and the production of anti-DNP IgG by peptide-pulsed splenic B cells from KLH-DNP-immunized donors [11]. Thus, the pMHCII-NP-induced TFH-like cells are not only transcriptionally similar to the effector-like TFH cells induced by conventional immunization but also clearly possess TFH-like functional properties.

The second of the above two questions was investigated via genetic manipulation in vivo. We previously showed that the Tet^+^ CD4^+^ T cells of BDC2.5 mi/I-A^g7^-NP-treated mice suppressed the proliferation of noncognate CD8^+^ T cells in response to peptide-pulsed DCs in vitro in an IL-10- and TGFβ-dependent manner [11]. Likewise, splenic tetramer^+^ (but not tetramer^−^) CD4^+^ T cells from BDC2.5 mi/I-A^g7^-NP-treated donors suppressed diabetes development in T-cell-reconstituted NOD.*Scid* hosts [11]. These observations demonstrated that the Tet^+^ CD4^+^ T-cell pools arising in vivo in response to pMHCII-NPs have dominant immunoregulatory properties [11]. Here, to test the specific immunoregulatory properties of the Tet^+^ TFH, TR1-like and TR1 subpools within these Tet^+^ pools, we resorted to an experimental disease model in the B6 background, which is more amenable to genetic manipulation than the NOD background. Since pMOG_38-49_/I-A^b^-coated NPs can reverse established pMOG_35-55_-induced EAE by triggering the formation and expansion of cognate TR1-like cells and the activation of downstream immunoregulatory networks similar to those seen in other disease models [11], we sought to determine whether selective abrogation of TR1 cell formation or IL-10 expression in TR1 cells is sufficient to abrogate the immunoregulatory properties of this compound. Indeed, deletion of *Il10* or *Prdm1* in the TR1 cells of pMOG_38-49_/I-A^b^-NP-treated mice almost completely abrogated the ability of this compound to reverse paralysis. Thus, pMHCII-NPs lack therapeutic activity in mice in which the pMHCII-NP-induced tetramer^+^ cell population lacks terminally differentiated TR1 cells or contains TR1 cells that cannot produce IL-10, indicating that the therapeutic activity of these compounds is mediated by neither Tet^+^ TFH-like cells nor transitional Tet^+^ TR1-like cells (neither of which express *Il10*).

It is important to note that pMHCII-NP-induced TR1 cells are similar to—but are not—TFR cells, which are negative regulators of the germinal center (GC) reaction [19]. Similar to TR1 cells, TFR cells coexpress PD-1, CTLA-4, ICOS, IL-10 and BLIMP1. However, unlike TR1 cells, TFR cells express CXCR5 (but not CCR5), BCL6, FOXP3 and CD25 and arise from natural FOXP3^+^ Treg cell precursors. Furthermore, whereas both *Bcl6* and *Foxp3* deletion can independently impair the development of TFR cells, *FOXP3* is dispensable for pMHCII-NP-induced TR1 cell development [11]. In addition, whereas deletion of *Prdm1* abrogated pMHCII-NP-induced TR1 cell formation, it enhanced the formation of both conventional FOXP3^+^ Treg cells and FOXP3^+^ TFR cells (see Supplementry Fig. 16C). Thus, whereas BLIMP1 expression in TFH cells functions to reprogram them into TR1 cells, BLIMP1 expression in TFR cells may help regulate their homeostasis.

In summary, the data reported herein demonstrate that murine FOXP3^–^ IL-10-producing TR1 cells arise from TFH cells in a BLIMP1-dependent manner. In addition, our work provides a comprehensive set of functional, phenotypic and transcriptional markers capable of distinguishing TR1 cells from their TFH cell precursors. These markers will likely play a pivotal role in guiding the clinical translation of compounds capable of promoting TR1-like cell formation in vivo for the treatment of autoimmune conditions, including pMHCII-based nanomedicines. They should also prove useful to quantify the contribution of this cell type to normal immune responses, to autoimmune diseases and to tumor progression in the context of cancer.

## Supplementary information

Supplementary Tables and Figures

Database 1

Database 2

Database 3

Database 4

Database 5

Database 6

Database 7

Database 8

Database 9

Database 10

Database 11

Database 12

Database 13

Database 14

Database 15

Database 16

Database 17

Database 18

Database 19

Database 20

Database 21

Database 22

Database 23

Database 24

Database 25

Database 26

Database 27

Database 28

Database 29

## Data Availability

All unique stable/reagents generated in this study are available from the corresponding authors with a completed Material Transfer Agreement. The raw RNAseq and scRNAseq data files have been uploaded into the GEO database (accession numbers: GSE173601; GSE173681; GSE173956; GSE182636).
