# Supplementary material for: A T follicular helper cell origin for T regulatory type 1 cells
Source: Cell Mol Immunol. 2023 Mar 27;20(5):489–511. doi: 10.1038/s41423-023-00989-z (PMC10202951; doi:10.1038/s41423-023-00989-z)
Supplement: Supplementary file 1 — Supplementary Tables and Figures [file 41423_2023_989_MOESM1_ESM.pdf]

Supplementary Table 1. Differential expression (DE) of 106 TR1/TFH/Treg-relevant genes in the various T-cell pools studied here (bulk RNAseq)

Gene type	Genes (protein)	Described in TR1 (ref)	Described in other Treg types (ref)	Described in TFH (ref)	DE in Tet+ (vs. Tet-)	Expressed in Tet+ (norm. counts)	DE in TFH+ (vs. Tconv)	DE in Tet+ (vs. Tconv)	DE in Tet+ (vs. TFH)	DE in Tet+ (vs. Tet-/CXCR5+/PD-1+) BASAL
Cell adhesion molecules	Cd226	+ (1)	ND	ND	+	65.23	- -	++	+++	+
	Itga2 (CD49b)	+ (1)	ND	ND	- -	1.38	- - -	NS	++	- - -
	Itgae (CD103)	+ (2)	+ (3)	ND	-	6.21	NS	+++	+++	NS
	Ocln	ND	ND	ND	+++	3.13	NS	+++	+++	+++
	S1pr2	ND	ND	+ (4)	+++	100.76	+++	+++	-	+
	Sell (CD62L)	- (5)	+ (6)	- (7)	- - -	101.52	- - -	- - -	+++	NS
	Selplg (Psgl1)	ND	ND	- (8)	- -	184.73	- - -	- -	+++	NS
Chemokyne receptors	Ccr5	+ (9)	+ (10)	+ (11)	+++	47.50	+++	+++	+++	++
	Ccr7	ND	+ (12)	- (13)	- -	174.10	- - -	- -	+++	NS
	Cxcr3	+ (14)	+ (12)	In some subsets (15)	++	153.84	+++	+++	+++	NS
	Cxcr4	ND	ND	+ (16)	NS	188.11	+++	++	NS	NS
	Cxcr5	ND	ND	+ (17)	+++	380.73	+++	+++	- -	NS
Co-estimulatory molecules	Cd28	+ (18)	ND	+ (19)	++	627.72	++	++	+	NS
	Cd40lg	ND	ND	+ (20)	+	182.86	++	++	+	NS
	Icos	+ (21)	+ (22)	+ (23)	++	337.09	+++	+++	-	NS
	Klrk1 (NKG2D)	ND	+ (24)	ND	- - -	0.24	NS	NS	NS	NS
	Sh2d1a (SAP)	ND	ND	+ (25)	+	78.17	+++	++	- -	-
	Tnfrsf4 (Ox40)	+ (26)	+ (27)	+ (28)	++	150.36	+++	+++	++	+
	Tnfrsf18 (GITR)	+ (18)	+ (29)	ND	+	128.13	+	++	+	NS
	Tnfsf4 (Ox40L)	ND	ND	ND	++	1.93	+++	+++	+++	NS
Co-inhibitory molecules	Ctla4	+ (30)	+ (31)	+ (32)	+++	663.75	+++	+++	++	++
	Fasl	ND	+ (33)	ND	- -	4.58	- -	NS	++	NS
	Havcr2 (TIM-3)	+ (34)	+ (35)	+ (36)	+++	7.95	NS	+++	+++	+++
	Lag3	+ (1)	+ (37)	ND	+++	347.33	+++	+++	+++	+++
	Pdcd1 (PD-1)	+ (9)	+ (38)	+ (17)	+++	383.95	+++	+++	- -	NS
	Tigit	+ (39)	+ (40)	+ (41)	+++	190.61	+++	+++	NS	NS
Cytokines	Ebi3 (IL27b)	ND	+ (42)	ND	+++	7.41	+++	+++	+	NS
	Ifng	+ (43)	ND	ND	+++	71.48	+++	+++	+++	NS
	Il10	+ (44)	+ (45)	+ (46)	+++	94.78	+++	+++	+++	+++
	Il21	+ (47)	ND	+ (48)	+++	526.65	+++	+++	NS	++
	Il4	- (44)	ND	+ (49)	+++	13.22	+++	+++	-	NS
	Il5	+ (44)	ND	ND	NS	0.01	NS	NS	NS	NS
	Mcub (Areg)	+	+ (50)	ND	NS	0.15	NS	NS	NS	NS
	Tgfb1	+ (44)	+ (45)	ND	NS	114.49	NS	NS	NS	NS
Cytokine receptors	Il10ra	+ (51)	+ (52)	ND	++	128.32	NS	++	++	NS
	Il12rb2	ND	+ (53)	ND	-	1.44	+++	+++	- -	NS
	Il21r	+ (47)	+ (54)	+ (48)	-	179.90	-	-	-	NS
	Il27ra	+ (55)	+ (56)	+ (57)	+	339.18	-	NS	+	NS
	IL7r (CD127)	- (21)	- (58)	- (59)	- - -	72.10	- - -	- - -	+++	NS
	IL17rc	ND	ND	ND	NS	0.03	NS	NS	NS	NS
	Il2ra (CD25)	- (18)	+ (60)	- (61)	- -	29.27	- -	NS	+++	NS
	Tgfbr1	- (51)	+ (62)	ND	NS	42.88	+	NS	-	NS
	Tgfbr2	- (51)	+ (63)	ND	-	384.31	-	-	NS	NS
	Tgfbr3	- (51)	ND	ND	-	100.03	- -	-	++	NS
	Atf6	- (51)	ND	ND	++	32.87	+++	+++	NS	+
	Ahr	+ (64)	ND	ND	+++	35.42	NS	+++	+++	+++
	Ajuba	ND	ND	ND	+++	1.89	NS	+++	+++	+++
	Ascl2	ND	ND	+ (65)	+++	35.56	+++	+++	-	NS
	Bach2	- (66)	+ (67)	- (68)	- - -	24.69	- - -	- - -	++	NS
	Batf	+ (69)	ND	+ (70)	+	61.67	+++	++	-	NS
	Bcl6	ND	ND	+ (71)	+++	238.34	+++	+++	- -	NS
	Bhlhe40	+ (51)	ND	ND	++	202.27	+++	+++	++	+
	Bmyc	- (51)	ND	ND	-	11.15	-	NS	NS	NS
	Cbfa2t3	ND	ND	ND	+++	25.96	+++	+++	-	NS

	Cebpa	ND	ND	+ (72)	+++	91.15	+++	+++	- -	NS
	Dbp	- (51)	ND	ND	NS	16.29	NS	NS	NS	NS
	E2f1	+ (51)	+ (73)	ND	NS	3.92	NS	NS	NS	NS
	Egr2	+ (74)	ND	ND	++	61.63	+++	+++	-	NS
	Elk4	- (51)	ND	ND	NS	172.79	NS	NS	-	NS
	Eomes	+ (75)	+ (76)	ND	NS	12.71	+	+++	++	- -
	FoxP1	ND	ND	- (77)	-	174.93	- -	-	+	NS
	FoxP3	ND	+ (78)	ND	- - -	30.31	++	NS	- -	- -
	Grhl1	ND	ND	ND	+++	38.45	+++	+++	NS	NS
	Hmgb2	+ (51)	ND	ND	+	38.12	++	++	NS	NS
	Id2	+ (51)	+ (79)	- (80)	+	180.43	-	++	++	+
	Id3	- (51)	+ (79)	+ (80)	+	37.87	+++	++	-	NS
	Irf1	+ (69)	ND	ND	-	282.24	-	NS	+	NS
	Irf4	+ (81)	+ (3)	+ (82)	+++	186.65	+++	+++	++	++
	Jdp2	ND	ND	ND	+++	11.36	+++	+++	- -	NS
	Klf2	ND	ND	- (83)	- -	116.99	- - -	- -	+++	NS
Transcription factors	Lef1	ND	ND	+ (84)	- - -	69.23	- - -	- - -	NS	- -
	Lilrb4a	ND	+ (85)	ND	#N/A	3.77	+++	+++	NS	NS
	Maf	+ (47)	ND	+ (86)	+++	642.44	+++	+++	-	NS
	Myb	- (51)	+ (87)	ND	+	28.09	+++	++	NS	NS
	Mybl2	+ (51)	ND	ND	NS	2.92	++	NS	- -	NS
	Myc	- (51)	- (88)	ND	- - -	19.34	- - -	- - -	NS	NS
	Nfia	ND	ND	ND	+++	22.14	+++	+++	- -	NS
	Nfil3	+ (34)	ND	ND	+++	62.68	+++	+++	++	+
	Nr1h3 (LXRα)	+ (51)	+ (89)	ND	NS	1.16	++	NS	-	NS
	Pax5	ND	ND	ND	+++	8.94	+++	+++	- -	NS
	Pax9	ND	ND	ND	+++	3.38	+++	+++	++	+++
	Pou2af1 (OcaB)	ND	ND	ND	+++	145.84	+++	+++	-	NS
	Prdm1 (Blimp-1)	+ (90)	ND	+ (91)	+++	100.20	NS	+++	+++	++
	Rbpj	+ (51)	+ (92)	ND	+	73.40	+	++	+	+
	Runx2	+ (51)	ND	ND	++	43.71	+++	+++	++	NS
	Rora	+ (51)	ND	- (93)	-	26.65	+++	+++	- -	- -
	S1pr1	ND	ND	- (83)	- -	141.34	- - -	- -	++	- -
	Six5	ND	ND	ND	+++	14.88	+++	+++	NS	NS
	Sox4	- (51)	- (94)	ND	- - -	1.07	NS	- -	NS	- - -
	Sox8	ND	ND	ND	+++	1.29	+++	+++	- -	NS
	Stat1	+ (95)	ND	+ (96)	NS	617.27	NS	NS	NS	NS
	Stat3	+ (97)	ND	+ (23)	+	345.04	+	+	NS	NS
	Stat4	ND	- (98)	+ (99)	+	142.56	++	++	-	NS
	Tbx21 (T-bet)	+ (75)	ND	- (84)	++	24.69	+++	+++	++	++
	Tcf7	ND	ND	+ (84)	NS	1519.30	NS	NS	NS	NS
	Tox2	ND	ND	+ (80)	+++	206.07	+++	+++	- -	NS
	Vdr	ND	+ (100)	ND	+++	65.44	+++	+++	NS	+++
	Zbtb16 (PLZF)	+ (51)	ND	ND	- - -	0.18	NS	NS	NS	NS
Secretion proteins	Chgb	ND	ND	+ (101)	NS	0.15	NS	NS	NS	NS
	Gzmb (Granzyme B)	+ (102)	+ (103)	ND	++	11.54	NS	+++	+++	+++
Enzymes	Cblb	ND	+ (104)	ND	NS	169.82	-	+	+	NS
	Entpd1 (CD39)	+ (105)	+ (106)	ND	+++	47.39	+++	+++	+++	++
	Itk	+ (107)	+ (108)	ND	NS	481.13	+	-	-	NS
	Nt5e (CD73)	+ (105)	+ (106)	+ (109)	+++	268.63	+++	+++	NS	NS
	Serpinb6b	+ (51)	+ (110)	ND	+	19.23	++	++	NS	NS
	Serpinb9	+ (51)	ND	ND	+	23.74	NS	+	NS	NS

+++	*FC>4*	(orange)	*Same results vs both KLH-induced TFH and Tet-/CXCR5+/PD-1+ cells from pMHCII-NP-treated mice*
++	*4>FC>2*	(pink)	*Different results vs both KLH-induced TFH and Tet-/CXCR5+/PD-1+ cells from pMHCII-NP-treated mice*
+	*2>FC>1*	*Text in red*	*Normalized counts below expression cut-off*
NS	*1>FC>-1*		
-	*-1>FC>-2*		
- -	*-2>FC>-4*		
- - -	*FC<-4*		

REFERENCES FOR SUPPLEMENTARY TABLE 1

1. Gagliani, N. *et al.* Coexpression of CD49b and LAG-3 identifies human and mouse T regulatory type 1 cells. *Nat. Med.* **19**, 739-746 (2013).

2. Duan, W., So, T., Mehta, A.K., Choi, H. & Croft, M. Inducible CD4+LAP+Foxp3- regulatory T cells suppress allergic inflammation. *J. Immunol.* **187**, 6499-6507 (2011).

3. Cretney, E. *et al.* The transcription factors Blimp-1 and IRF4 jointly control the differentiation and function of effector regulatory T cells. *Nat. Immunol.* **12**, 304-311 (2011).

4. Moriyama, S. *et al.* Sphingosine-1-phosphate receptor 2 is critical for follicular helper T cell retention in germinal centers. *J. Exp. Med.* **211**, 1297-1305 (2014).

5. Bollyky, P.L. *et al.* ECM components guide IL-10 producing regulatory T-cell (TR1) induction from effector memory T-cell precursors. *Proc. Natl. Acad. Sci. U. S. A.* **108**, 7938-7943 (2011).

6. Biswas, M., Kumar, S.R.P., Terhorst, C. & Herzog, R.W. Gene Therapy With Regulatory T Cells: A Beneficial Alliance. *Front. Immunol.* **9**, 554 (2018).

7. Fazilleau, N., McHeyzer-Williams, L.J., Rosen, H. & McHeyzer-Williams, M.G. The function of follicular helper T cells is regulated by the strength of T cell antigen receptor binding. *Nat. Immunol.* **10**, 375-384 (2009).

8. Poholek, A.C. *et al.* In vivo regulation of Bcl6 and T follicular helper cell development. *J. Immunol.* **185**, 313-326 (2010).

9. Alfen, J.S. *et al.* Intestinal IFN-gamma-producing type 1 regulatory T cells coexpress CCR5 and programmed cell death protein 1 and downregulate IL-10 in the inflamed guts of patients with inflammatory bowel disease. *J. Allergy Clin. Immunol.* **142**, 1537-1547 e1538 (2018).

10. Bystry, R.S., Aluvihare, V., Welch, K.A., Kallikourdis, M. & Betz, A.G. B cells and professional APCs recruit regulatory T cells via CCL4. *Nat. Immunol.* **2**, 1126-1132 (2001).

11. Miller, S.M. *et al.* Follicular Regulatory T Cells Are Highly Permissive to R5-Tropic HIV-1. *J. Virol.* **91** (2017).

12. Smigiel, K.S. *et al.* CCR7 provides localized access to IL-2 and defines homeostatically distinct regulatory T cell subsets. *J. Exp. Med.* **211**, 121-136 (2014).

13. Haynes, N.M. *et al.* Role of CXCR5 and CCR7 in follicular Th cell positioning and appearance of a programmed cell death gene-1high germinal center-associated subpopulation. *J. Immunol.* **179**, 5099-5108 (2007).

14. Kunicki, M.A., Amaya Hernandez, L.C., Davis, K.L., Bacchetta, R. & Roncarolo, M.G. Identity and Diversity of Human Peripheral Th and T Regulatory Cells Defined by Single-Cell Mass Cytometry. *J. Immunol.* **200**, 336-346 (2018).

15. Chevalier, N. Quantifying helper cell function of human TFH cells in vitro. *Methods Mol. Biol.* **1291**, 209-226 (2015).

16. Allen, C.D. *et al.* Germinal center dark and light zone organization is mediated by CXCR4 and CXCR5. *Nat. Immunol.* **5**, 943-952 (2004).

17. Crotty, S. Follicular helper CD4 T cells (TFH). *Annu. Rev. Immunol.* **29**, 621-663 (2011).

18. Zeng, H., Zhang, R., Jin, B. & Chen, L. Type 1 regulatory T cells: a new mechanism of peripheral immune tolerance. *Cell. Mol. Immunol.* **12**, 566-571 (2015).

19. Linterman, M.A. *et al.* CD28 expression is required after T cell priming for helper T cell responses and protective immunity to infection. *Elife* **3** (2014).

20. Vinuesa, C.G., Tangye, S.G., Moser, B. & Mackay, C.R. Follicular B helper T cells in antibody responses and autoimmunity. *Nat. Rev. Immunol.* **5**, 853-865 (2005).

21. Haringer, B., Lozza, L., Steckel, B. & Geginat, J. Identification and characterization of IL-10/IFN-gamma-producing effector-like T cells with regulatory function in human blood. *J. Exp. Med.* **206**, 1009-1017 (2009).

22. Strauss, L. *et al.* Expression of ICOS on human melanoma-infiltrating CD4+CD25highFoxp3+ T regulatory cells: implications and impact on tumor-mediated immune suppression. *J. Immunol.* **180**, 2967-2980 (2008).

23. Nurieva, R.I. *et al.* Generation of T follicular helper cells is mediated by interleukin-21 but independent of T helper 1, 2, or 17 cell lineages. *Immunity* **29**, 138-149 (2008).

24. Fujio, K., Okamura, T. & Yamamoto, K. The Family of IL-10-secreting CD4+ T cells. *Adv. Immunol.* **105**, 99-130 (2010).

25. Crotty, S., Kersh, E.N., Cannons, J., Schwartzberg, P.L. & Ahmed, R. SAP is required for generating long-term humoral immunity. *Nature* **421**, 282-287 (2003).

26. Ito, T. *et al.* OX40 ligand shuts down IL-10-producing regulatory T cells. *Proc. Natl. Acad. Sci. U. S. A.* **103**, 13138-13143 (2006).

27. Fontenot, J.D. *et al.* Regulatory T cell lineage specification by the forkhead transcription factor foxp3. *Immunity* **22**, 329-341 (2005).

28. Jacquemin, C. *et al.* OX40 Ligand Contributes to Human Lupus Pathogenesis by Promoting T Follicular Helper Response. *Immunity* **42**, 1159-1170 (2015).

29. Shimizu, J., Yamazaki, S., Takahashi, T., Ishida, Y. & Sakaguchi, S. Stimulation of CD25(+)CD4(+) regulatory T cells through GITR breaks immunological self-tolerance. *Nat. Immunol.* **3**, 135-142 (2002).

30. Bacchetta, R. *et al.* Growth and expansion of human T regulatory type 1 cells are independent from TCR activation but require exogenous cytokines. *Eur. J. Immunol.* **32**, 2237-2245 (2002).

31. Walker, L.S. Treg and CTLA-4: two intertwining pathways to immune tolerance. *J. Autoimmun.* **45**, 49-57 (2013).

32. Sage, P.T., Paterson, A.M., Lovitch, S.B. & Sharpe, A.H. The coinhibitory receptor CTLA-4 controls B cell responses by modulating T follicular helper, T follicular regulatory, and T regulatory cells. *Immunity* **41**, 1026-1039 (2014).

33. Gorbachev, A.V. & Fairchild, R.L. CD4+CD25+ regulatory T cells utilize FasL as a mechanism to restrict DC priming functions in cutaneous immune responses. *Eur. J. Immunol.* **40**, 2006-2015 (2010).

34. Zhu, C. *et al.* An IL-27/NFIL3 signalling axis drives Tim-3 and IL-10 expression and T-cell dysfunction. *Nat. Comm.* **6**, 6072 (2015).

35. Gupta, S. *et al.* Allograft rejection is restrained by short-lived TIM-3+PD-1+Foxp3+ Tregs. *J. Clin. Invest.* **122**, 2395-2404 (2012).

36. Zhu, S., Lin, J., Qiao, G., Wang, X. & Xu, Y. Tim-3 identifies exhausted follicular helper T cells in breast cancer patients. *Immunobiology* **221**, 986-993 (2016).

37. Huang, C.T. *et al.* Role of LAG-3 in regulatory T cells. *Immunity* **21**, 503-513 (2004).

38. Chen, X. *et al.* PD-1 regulates extrathymic regulatory T-cell differentiation. *Eur. J. Immunol.* **44**, 2603-2616 (2014).

39. Burton, B.R. *et al.* Sequential transcriptional changes dictate safe and effective antigen-specific immunotherapy. *Nat. Comm.* **5**, 4741 (2014).

40. Yu, X. *et al.* The surface protein TIGIT suppresses T cell activation by promoting the generation of mature immunoregulatory dendritic cells. *Nat. Immunol.* **10**, 48-57 (2009).

41. Godefroy, E., Zhong, H., Pham, P., Friedman, D. & Yazdanbakhsh, K. TIGIT-positive circulating follicular helper T cells display robust B-cell help functions: potential role in sickle cell alloimmunization. *Haematologica* **100**, 1415-1425 (2015).

42. Collison, L.W. *et al.* The inhibitory cytokine IL-35 contributes to regulatory T-cell function. *Nature* **450**, 566-569 (2007).

43. Facciotti, F. *et al.* IL-10-producing forkhead box protein 3-negative regulatory T cells inhibit B-cell responses and are involved in systemic lupus erythematosus. *J. Allergy Clin. Immunol.* **137**, 318-321 e315 (2016).

44. Groux, H. *et al.* A CD4+ T-cell subset inhibits antigen-specific T-cell responses and prevents colitis. *Nature* **389**, 737-742 (1997).

45. Liu, H., Hu, B., Xu, D. & Liew, F.Y. CD4+CD25+ regulatory T cells cure murine colitis: the role of IL-10, TGF-beta, and CTLA4. *J. Immunol.* **171**, 5012-5017 (2003).

46. Zhu, Y., Zou, L. & Liu, Y.C. T follicular helper cells, T follicular regulatory cells and autoimmunity. *Int. Immunol.* **28**, 173-179 (2016).

47. Pot, C. *et al.* Cutting edge: IL-27 induces the transcription factor c-Maf, cytokine IL-21, and the costimulatory receptor ICOS that coordinately act together to promote differentiation of IL-10-producing Tr1 cells. *J. Immunol.* **183**, 797-801 (2009).

48. Chtanova, T. *et al.* T follicular helper cells express a distinctive transcriptional profile, reflecting their role as non-Th1/Th2 effector cells that provide help for B cells. *J. Immunol.* **173**, 68-78 (2004).

49. King, I.L. & Mohrs, M. IL-4-producing CD4+ T cells in reactive lymph nodes during helminth infection are T follicular helper cells. *J. Exp. Med.* **206**, 1001-1007 (2009).

50. Burzyn, D. *et al.* A special population of regulatory T cells potentiates muscle repair. *Cell* **155**, 1282-1295 (2013).

51. Brockmann, L. *et al.* Molecular and functional heterogeneity of IL-10-producing CD4(+) T cells. *Nat. Comm.* **9**, 5457 (2018).

52. Diefenhardt, P. *et al.* IL-10 Receptor Signaling Empowers Regulatory T Cells to Control Th17 Responses and Protect from GN. *J. Am. Soc. Nephrol.* **29**, 1825-1837 (2018).

53. Zhao, Z. *et al.* IL-12R beta 2 promotes the development of CD4+CD25+ regulatory T cells. *J. Immunol.* **181**, 3870-3876 (2008).

54. Comes, A. *et al.* CD25+ regulatory T cell depletion augments immunotherapy of micrometastases by an IL-21-secreting cellular vaccine. *J. Immunol.* **176**, 1750-1758 (2006).

55. Meka, R.R., Venkatesha, S.H., Dudics, S., Acharya, B. & Moudgil, K.D. IL-27-induced modulation of autoimmunity and its therapeutic potential. *Autoimmun Rev* **14**, 1131-1141 (2015).

56. Villarino, A.V. *et al.* Positive and negative regulation of the IL-27 receptor during lymphoid cell activation. *J. Immunol.* **174**, 7684-7691 (2005).

57. Batten, M. *et al.* IL-27 supports germinal center function by enhancing IL-21 production and the function of T follicular helper cells. *J. Exp. Med.* **207**, 2895-2906 (2010).

58. Liu, W. *et al.* CD127 expression inversely correlates with FoxP3 and suppressive function of human CD4+ T reg cells. *J. Exp. Med.* **203**, 1701-1711 (2006).

59. McDonald, P.W. *et al.* IL-7 signalling represses Bcl-6 and the TFH gene program. *Nat. Comm.* **7**, 10285 (2016).

60. Sakaguchi, S., Sakaguchi, N., Asano, M., Itoh, M. & Toda, M. Immunologic self-tolerance maintained by activated T cells expressing IL-2 receptor alpha-chains (CD25). Breakdown of a single mechanism of self-tolerance causes various autoimmune diseases. *J. Immunol.* **155**, 1151-1164 (1995).

61. Ballesteros-Tato, A. *et al.* Interleukin-2 inhibits germinal center formation by limiting T follicular helper cell differentiation. *Immunity* **36**, 847-856 (2012).

62. Liu, Y. *et al.* A critical function for TGF-beta signaling in the development of natural CD4+CD25+Foxp3+ regulatory T cells. *Nat. Immunol.* **9**, 632-640 (2008).

63. Ouyang, W., Beckett, O., Ma, Q. & Li, M.O. Transforming growth factor-beta signaling curbs thymic negative selection promoting regulatory T cell development. *Immunity* **32**, 642-653 (2010).

64. Apetoh, L. *et al.* The aryl hydrocarbon receptor interacts with c-Maf to promote the differentiation of type 1 regulatory T cells induced by IL-27. *Nat. Immunol.* **11**, 854-861 (2010).

65. Liu, X. *et al.* Transcription factor achaete-scute homologue 2 initiates follicular T-helper-cell development. *Nature* **507**, 513-518 (2014).

66. Edwards, C.L. *et al.* The Role of BACH2 in T Cells in Experimental Malaria Caused by Plasmodium chabaudi chabaudi AS. *Front. Immunol.* **9**, 2578 (2018).

67. Grant, F.M. *et al.* BACH2 drives quiescence and maintenance of resting Treg cells to promote homeostasis and cancer immunosuppression. *J. Exp. Med.* **217** (2020).

68. Lahmann, A. *et al.* Bach2 Controls T Follicular Helper Cells by Direct Repression of Bcl-6. *J. Immunol.* **202**, 2229-2239 (2019).

69. Karwacz, K. *et al.* Critical role of IRF1 and BATF in forming chromatin landscape during type 1 regulatory cell differentiation. *Nat. Immunol.* **18**, 412-421 (2017).

70. Ise, W. *et al.* The transcription factor BATF controls the global regulators of class-switch recombination in both B cells and T cells. *Nat. Immunol.* **12**, 536-543 (2011).

71. Nurieva, R.I. *et al.* Bcl6 mediates the development of T follicular helper cells. *Science* **325**, 1001-1005 (2009).

72. Tanaka, S. *et al.* CCAAT/enhancer-binding protein alpha negatively regulates IFN-gamma expression in T cells. *J. Immunol.* **193**, 6152-6160 (2014).

73. Bao, R. *et al.* Adenosine and the adenosine A2A receptor agonist, CGS21680, upregulate CD39 and CD73 expression through E2F-1 and CREB in regulatory T cells isolated from septic mice. *Int. J. Mol. Med.* **38**, 969-975 (2016).

74. Okamura, T. *et al.* CD4+CD25-LAG3+ regulatory T cells controlled by the transcription factor Egr-2. *Proc. Natl. Acad. Sci. U. S. A.* **106**, 13974-13979 (2009).

75. Zhang, P. *et al.* Eomesodermin promotes the development of type 1 regulatory T (TR1) cells. *Sci Immunol* **2** (2017).

76. Schaer, D.A. *et al.* GITR pathway activation abrogates tumor immune suppression through loss of regulatory T cell lineage stability. *Cancer Immunol Res* **1**, 320-331 (2013).

77. Wang, H. *et al.* The transcription factor Foxp1 is a critical negative regulator of the differentiation of follicular helper T cells. *Nat. Immunol.* **15**, 667-675 (2014).

78. Hori, S., Nomura, T. & Sakaguchi, S. Control of regulatory T cell development by the transcription factor Foxp3. *Science* **299**, 1057-1061 (2003).

79. Miyazaki, M. *et al.* Id2 and Id3 maintain the regulatory T cell pool to suppress inflammatory disease. *Nat. Immunol.* **15**, 767-776 (2014).

80. Choi, Y.S. *et al.* Bcl6 expressing follicular helper CD4 T cells are fate committed early and have the capacity to form memory. *J. Immunol.* **190**, 4014-4026 (2013).

81. Jin, J.O., Han, X. & Yu, Q. Interleukin-6 induces the generation of IL-10-producing Tr1 cells and suppresses autoimmune tissue inflammation. *J. Autoimmun.* **40**, 28-44 (2013).

82. Kwon, H. *et al.* Analysis of interleukin-21-induced Prdm1 gene regulation reveals functional cooperation of STAT3 and IRF4 transcription factors. *Immunity* **31**, 941-952 (2009).

83. Lee, J.Y. *et al.* The transcription factor KLF2 restrains CD4(+) T follicular helper cell differentiation. *Immunity* **42**, 252-264 (2015).

84. Choi, Y.S. *et al.* LEF-1 and TCF-1 orchestrate T(FH) differentiation by regulating differentiation circuits upstream of the transcriptional repressor Bcl6. *Nat. Immunol.* **16**, 980-990 (2015).

85. Ulges, A. *et al.* Protein kinase CK2 enables regulatory T cells to suppress excessive TH2 responses in vivo. *Nat. Immunol.* **16**, 267-275 (2015).

86. Bauquet, A.T. *et al.* The costimulatory molecule ICOS regulates the expression of c-Maf and IL-21 in the development of follicular T helper cells and TH-17 cells. *Nat. Immunol.* **10**, 167-175 (2009).

87. Dias, S. *et al.* Effector Regulatory T Cell Differentiation and Immune Homeostasis Depend on the Transcription Factor Myb. *Immunity* **46**, 78-91 (2017).

88. Angelin, A. *et al.* Foxp3 Reprograms T Cell Metabolism to Function in Low-Glucose, High-Lactate Environments. *Cell Metab.* **25**, 1282-1293 e1287 (2017).

89. Herold, M. *et al.* Liver X receptor activation promotes differentiation of regulatory T cells. *PLoS One* **12**, e0184985 (2017).

90. Heinemann, C. *et al.* IL-27 and IL-12 oppose pro-inflammatory IL-23 in CD4+ T cells by inducing Blimp1. *Nat. Comm.* **5**, 3770 (2014).

91. Johnston, R.J. *et al.* Bcl6 and Blimp-1 are reciprocal and antagonistic regulators of T follicular helper cell differentiation. *Science* **325**, 1006-1010 (2009).

92. Fu, T. *et al.* Accelerated acute allograft rejection accompanied by enhanced T-cell proliferation and attenuated Treg function in RBP-J deficient mice. *Mol. Immunol.* **48**, 751-759 (2011).

93. Baumjohann, D. *et al.* The microRNA cluster miR-17 approximately 92 promotes TFH cell differentiation and represses subset-inappropriate gene expression. *Nat. Immunol.* **14**, 840-848 (2013).

94. Komatsu, N. *et al.* Pathogenic conversion of Foxp3+ T cells into TH17 cells in autoimmune arthritis. *Nat. Med.* **20**, 62-68 (2014).

95. Wang, H. *et al.* IL-27 induces the differentiation of Tr1-like cells from human naive CD4+ T cells via the phosphorylation of STAT1 and STAT3. *Immunol. Lett.* **136**, 21-28 (2011).

96. Choi, Y.S., Eto, D., Yang, J.A., Lao, C. & Crotty, S. Cutting edge: STAT1 is required for IL-6-mediated Bcl6 induction for early follicular helper cell differentiation. *J. Immunol.* **190**, 3049-3053 (2013).

97. Iwasaki, Y. *et al.* Egr-2 transcription factor is required for Blimp-1-mediated IL-10 production in IL-27-stimulated CD4+ T cells. *Eur. J. Immunol.* **43**, 1063-1073 (2013).

98. Koch, M.A. *et al.* T-bet(+) Treg cells undergo abortive Th1 cell differentiation due to impaired expression of IL-12 receptor beta2. *Immunity* **37**, 501-510 (2012).

99. Schmitt, N. *et al.* IL-12 receptor beta1 deficiency alters in vivo T follicular helper cell response in humans. *Blood* **121**, 3375-3385 (2013).

100. Ghoreishi, M. *et al.* Expansion of antigen-specific regulatory T cells with the topical vitamin d analog calcipotriol. *J. Immunol.* **182**, 6071-6078 (2009).

101. Papa, I. *et al.* TFH-derived dopamine accelerates productive synapses in germinal centres. *Nature* **547**, 318-323 (2017).

102. Grossman, W.J. *et al.* Differential expression of granzymes A and B in human cytotoxic lymphocyte subsets and T regulatory cells. *Blood* **104**, 2840-2848 (2004).

103. Cao, X. *et al.* Granzyme B and perforin are important for regulatory T cell-mediated suppression of tumor clearance. *Immunity* **27**, 635-646 (2007).

104. Chen, Z. *et al.* Ubiquitination signals critical to regulatory T cell development and function. *Int. Immunopharmacol.* **16**, 348-352 (2013).

105. Mandapathil, M. *et al.* Adenosine and prostaglandin E2 cooperate in the suppression of immune responses mediated by adaptive regulatory T cells. *J. Biol. Chem.* **285**, 27571-27580 (2010).

106. Deaglio, S. *et al.* Adenosine generation catalyzed by CD39 and CD73 expressed on regulatory T cells mediates immune suppression. *J. Exp. Med.* **204**, 1257-1265 (2007).

107. Huang, W., Solouki, S., Koylass, N., Zheng, S.G. & August, A. ITK signalling via the Ras/IRF4 pathway regulates the development and function of Tr1 cells. *Nat. Comm.* **8**, 15871 (2017).

108. Huang, W., Jeong, A.R., Kannan, A.K., Huang, L. & August, A. IL-2-inducible T cell kinase tunes T regulatory cell development and is required for suppressive function. *J. Immunol.* **193**, 2267-2272 (2014).

109. Iyer, S.S. *et al.* Identification of novel markers for mouse CD4(+) T follicular helper cells. *Eur. J. Immunol.* **43**, 3219-3232 (2013).

110. Azzi, J. *et al.* Serine protease inhibitor 6 plays a critical role in protecting murine granzyme B-producing regulatory T cells. *J. Immunol.* **191**, 2319-2327 (2013).

Supplementary Table 2. Differential expression (DE) of 106 TR1/TFH/Treg-relevant genes in BDC2.5mi/IAg7 and Fla462/IAb Tet+ vs. Tconv cells (bulk RNAseq)

Gene type	Genes (protein)	DE in BDC2.5mi/IAg7 Tet+ (vs. Tet-)	Expressed in BDC2.5mi/IAg7 Tet+ (normalized counts)	DE in FLA462/IAb Tet+ (vs. Tet-)	Expressed in FLA462/IAb Tet+ (normalized counts)
Cell adhesion molecules	Cd226	+	65.23	+	45.99
	Itga2 (CD49b)	- -	1.38	NS	0.54
	Itgae (CD103)	-	6.21	- -	2.25
	Ocln	++	3.13	NS	1.79
	S1pr2	++	100.76	++	61.50
	Sell (CD62L)	- -	101.52	- -	81.91
	Selplg (Psgl1)	- -	184.73	- -	139.53
Chemokyne receptors	Ccr5	++	47.50	NS	16.18
	Ccr7	- -	174.10	- -	1096.60
	Cxcr3	++	153.84	NS	83.47
	Cxcr4	NS	188.11	++	264.57
	Cxcr5	++	380.73	++	225.33
Co-stimulatory molecules	Cd28	++	627.72	NS	1127.26
	Cd40lg	+	182.86	+	412.86
	Icos	++	337.09	NS	593.69
	Klrk1 (NKG2D)	- -	0.24	NS	0.22
	Sh2d1a (SAP)	+	78.17	++	131.28
	Tnfrsf4 (Ox40)	++	150.36	NS	329.34
	Tnfrsf18 (GITR)	+	128.13	NS	135.06
	Tnfsf4 (Ox40L)	++	1.93	++	6.89
Co-inhibitory molecules	Ctla4	++	663.75	++	418.96
	Fasl	- -	4.58	NS	37.41
	Havcr2 (TIM-3)	++	7.95	NS	1.05
	Lag3	++	347.33	++	56.39
	Pdcd1 (PD-1)	++	383.95	++	508.50
	Tigit	++	190.61	++	104.86
Cytokines	Ebi3 (IL27b)	++	7.41	++	13.92
	Ifng	++	71.48	++	68.87
	Il10	++	94.78	++	45.89
	Il21	++	526.65	++	238.83
	Il4	++	13.22	++	27.38
	Il5	NS	0.01	#N/A	#N/A
	Mcub (Areg)	NS	0.15	NS	15.43
	Tgfb1	NS	114.49	NS	172.54
Cytokine receptors	Il10ra	++	128.32	NS	31.50
	Il12rb2	-	1.44	- -	1.38
	Il21r	-	179.90	- -	297.94
	Il27ra	+	339.18	NS	342.57
	IL7r (CD127)	- -	72.10	- -	49.43
	IL17rc	NS	0.03	NS	0.22
	Il2ra (CD25)	- -	29.27	- -	6.04
	Tgfbr1	NS	42.88	NS	26.98
	Tgfbr2	-	384.31	NS	444.77
	Tgfbr3	-	100.03	-	45.09
	Atf6	++	32.87	NS	29.05
	Ahr	++	35.42	NS	31.11
	Ajuba	++	1.89	NS	0.70
	Ascl2	++	35.56	++	41.10
	Batf	+	61.67	+	117.71
	Bcl6	++	238.34	NS	191.12
	Bhlhe40	++	202.27	++	1297.01
	Bmyc	-	11.15	NS	30.32
	Cbfa2t3	++	25.96	++	14.53
	Cebpa	++	91.15	++	50.92

Transcription factors	Dbp	NS	16.29	NS	8.41
	E2f1	NS	3.92	NS	5.82
	Egr2	++	61.63	NS	96.53
	Elk4	NS	172.79	NS	92.34
	Eomes	NS	12.71	- -	5.19
	FoxP1	-	174.93	NS	282.05
	FoxP3	- -	30.31	- -	16.33
	Grhl1	++	38.45	++	12.01
	Hmgb2	+	38.12	NS	121.17
	Id2	+	180.43	NS	188.96
	Id3	+	37.87	++	35.49
	Irf1	-	282.24	- -	99.68
	Irf4	++	186.65	NS	22.46
	Jdp2	++	11.36	++	10.75
	Klf2	- -	116.99	- -	549.06
	Lef1	- -	69.23	- -	211.71
	Lilrb4a	#N/A	0.00	#N/A	#N/A
	Maf	++	642.44	++	346.78
	Myb	+	28.09	-	51.48
	Mybl2	NS	2.92	NS	1.57
	Myc	- -	19.34	NS	53.86
	Nfia	++	22.14	++	13.47
	Nfil3	++	62.68	++	320.66
	Nr1h3 (LXRα)	NS	1.16	NS	2.13
	Pax5	++	8.94	++	3.31
	Pax9	++	3.38	NS	0.45
	Prdm1 (Blimp-1)	++	100.20	NS	10.05
	Rbpj	+	73.40	NS	76.01
	Runx2	++	43.71	NS	15.38
	Rora	-	26.65	NS	161.75
	S1pr1	- -	141.34	- -	363.10
	Six5	++	14.88	++	1.14
	Sox4	- -	1.07	- -	9.85
	Sox8	++	1.29	NS	0.93
	Stat1	NS	617.27	NS	569.13
	Stat3	+	345.04	NS	479.20
	Stat4	+	142.56	+	175.61
	Tbx21 (T-bet)	++	24.69	NS	47.35
	Tcf7	NS	1519.30	+	2987.52
	Tox2	++	206.07	++	649.25
	Vdr	++	65.44	++	69.11
	Zbtb16 (PLZF)	- -	0.18	- -	0.41
Secretion proteins	Chgb	NS	0.15	#N/A	#N/A
	Gzmb (Granzyme B)	++	11.54	- -	0.50
Enzymes	Cblb	NS	169.82	NS	238.13
	Entpd1 (CD39)	++	47.39	NS	13.66
	Itk	NS	481.13	NS	633.92
	Nt5e (CD73)	++	268.63	++	235.00
	Serpinb6b	+	19.23	NS	14.20
	Serpinb9	+	23.74	-	20.43

+++	*FC>4*	[shaded box]	*Expression consistent between Tet+ cells*
++	*4>FC>2*	*Text in red*	*Normalized counts below expression cut-off*
+	*2>FC>1*	*NA*	*Not detected*
NS	*1>FC>-1*	*NS*	*Non-significant*
-	*-1>FC>-2*		
- -	*-2>FC>-4*		
- - -	*FC<-4*		

Supplementary Table 3. Differential expression of the 106 TR1/TFH/Treg gene selection in the various SMARTseq2 and 10x scRNAseq cell clusters

Gene type	Genes	SMARTseq2 of pooled Tet+ vs Tconv	10X scRNAseq of pooled Tet+ vs Tconv	10X scRNAseq BDC Tet+ vs INS Tet+	10x scRNAseq TR1 cluster vs Tconv cluster	10x scRNAseq TFH cluster vs Tconv cluster	10x scRNAseq TR1 cluster vs TFH cluster	Bulk RNAseq TFH vs Tconv
Cell adhesion molecules	Cd226	+++	NS	NS	++	NS	++	- -
	Itga2 (CD49b)	NS	NS	- -	++	NS	+++	- - -
	Itgae (CD103)	NS	NS	- -	+++	NS	+++	NS
	Ocln	NS	+++	NS	+++	+++	NS	NS
	S1pr2	NS	+++	NS	+++	+++	- - -	+++
	Sell (CD62L)	- - -	- -	NS	- - -	- - -	++	- - -
	Selplg (Psgl1)	- -	NS	NS	NS	- - -	++	- - -
Chemokyne receptors	Ccr5	NS	+++	NS	+++	+++	+++	+++
	Ccr7	- -	- -	NS	- -	- - -	++	- - -
	Cxcr3	+++	++	NS	+++	+++	++	+++
	Cxcr4	+++	++	NS	NS	+++	- - -	+++
	Cxcr5	+++	+++	++	+++	+++	- - -	+++
Co-estimulatory molecules	Cd28	+++	NS	NS	+	++	NS	++
	Cd40lg	+	NS	NS	NS	++	-	++
	Icos	+++	+++	NS	+++	+++	NS	+++
	Klrk1 (NKG2D)	NS	NS	NS	+++	NS	+++	NS
	Sh2d1a (SAP)	+	NS	NS	++	++	NS	+++
	Tnfrsf4 (Ox40)	+++	++	NS	+++	+++	++	+++
	Tnfrsf18 (GITR)	+++	NS	NS	++	NS	NS	+
	Tnfsf4 (Ox40L)	NS	++	NS	+++	NS	NS	+++
Co-inhibitory molecules	Ctla4	+++	+++	NS	+++	+++	++	+++
	Fasl	+++	NS	NS	++	- -	+++	- -
	Havcr2 (TIM-3)	NS	+++	- -	+++	+++	+++	NS
	Lag3	+++	+++	NS	+++	+++	NS	+++
	Pdcd1 (PD-1)	+++	+++	NS	+++	+++	- - -	+++
	Tigit	+++	+++	NS	+++	+++	NS	+++
Cytokines	Ebi3 (IL27b)	NS	+++	NS	+++	+++	++	+++
	Ifng	+++	++	NS	+++	+++	++	+++
	Il10	+++	+++	NS	+++	+++	+++	+++
	Il21	+++	+++	NS	+++	+++	NS	+++
	Il4	+++	+++	NS	+++	+++	- - -	+++
	Il5	NS	#N/A	#N/A	#N/A	#N/A	#N/A	NS
	Mcub (Areg)	NS	NS	NS	NS	NS	NS	NS
	Tgfb1	NS	NS	NS	NS	NS	NS	NS
Cytokine receptors	Il10ra	+++	+	NS	+++	++	NS	NS
	Il12rb2	NS	NS	NS	+++	NS	++	+++
	Il21r	+++	NS	NS	NS	NS	NS	-
	Il27ra	+	NS	NS	NS	NS	NS	-
	IL7r (CD127)	- - -	- -	NS	- -	- - -	++	- - -
	IL17rc	NS	NS	NS	NS	NS	NS	NS
	Il2ra (CD25)	- - -	NS	NS	++	NS	+++	- -
	Tgfbr1	NS	NS	NS	NS	NS	NS	+
	Tgfbr2	++	NS	NS	NS	NS	NS	-
	Tgfbr3	NS	NS	NS	NS	NS	NS	- -
	Atf6	NS	++	NS	+++	+++	NS	+++
	Ahr	NS	+++	NS	+++	+++	+++	NS
	Ajuba	NS	+++	NS	+++	NS	NS	NS
	Ascl2	+++	+++	NS	+++	+++	- - -	+++
	Bach2	- - -	- -	NS	- -	- - -	+	- - -
	Batf	+++	NS	NS	++	++	NS	+++
	Bcl6	+++	+++	NS	++	+++	- - -	+++
	Bhlhe40	+++	++	NS	+++	+++	NS	+++
	Bmyc	- - -	NS	NS	NS	NS	NS	-
	Cbfa2t3	NS	+++	NS	+++	+++	- - -	+++
	Cebpa	NS	+++	NS	+++	+++	- - -	+++
	Dbp	NS	NS	NS	NS	NS	NS	NS
	E2f1	NS	++	- -	+++	NS	++	NS
	Egr2	NS	++	NS	++	+++	- -	+++
	Elk4	++	NS	NS	NS	++	NS	NS
	Eomes	NS	NS	NS	++	+++	NS	+
	FoxP1	- -	NS	NS	NS	NS	NS	- -
	FoxP3	- - -	NS	- -	+++	- -	+++	++
	Grhl1	NS	+++	++	+++	+++	NS	+++
	Hmgb2	++	+	NS	++	NS	+	++
	Id2	+++	NS	NS	++	NS	+	-
	Id3	-	++	++	NS	+++	- - -	+++
	Irf1	+++	NS	NS	NS	NS	NS	-
	Irf4	NS	++	++	+++	++	++	+++
	Jdp2	NS	+++	NS	+++	+++	- - -	+++

Transcription factors	Klf2	- - -	NS	- -	-	- - -	+++	- - -
	Lef1	- - -	- -	-	- - -	- - -	NS	- - -
	Lilrb4a	NS	++	- - -	+++	NS	+++	#N/A
	Maf	+++	+++	NS	+++	+++	NS	+++
	Myb	NS	++	NS	++	+++	NS	+++
	Mybl2	NS	NS	NS	+++	NS	+++	++
	Myc	- - -	- -	NS	- -	- - -	+++	- - -
	Nfia	+++	+++	NS	+++	+++	- - -	+++
	Nfil3	+++	+++	NS	+++	+++	NS	+++
	Nr1h3 (LXRα)	NS	++	NS	NS	NS	NS	++
	Pax5	NS	+++	NS	++	+++	- - -	#N/A
	Pax9	NS	+++	NS	NS	+++	- - -	+++
	Pou2af1 (OcaB)	NS	+++	++	+++	+++	NS	+++
	Prdm1 (Blimp-1)	+++	+++	NS	+++	NS	+++	NS
	Rbpj	+++	+	NS	++	+	NS	+
	Runx2	NS	+++	NS	+++	+++	++	+++
	Rora	+	NS	NS	+++	++	NS	+++
	S1pr1	- - -	NS	NS	-	- - -	++	- - -
	Six5	NS	+++	++	+++	+++	NS	+++
	Sox4	NS	NS	NS	- - -	- - -	NS	NS
	Sox8	NS	+++	NS	+++	+++	- - -	+++
	Stat1	-	NS	NS	NS	NS	NS	NS
	Stat3	+++	NS	NS	NS	+	NS	+
	Stat4	++	+	NS	++	++	NS	++
	Tbx21 (T-bet)	NS	++	NS	+++	+++	++	+++
	Tcf7	+	NS	NS	NS	+	- -	NS
	Tox2	+++	+++	NS	+++	+++	- - -	+++
	Vdr	NS	+++	NS	+++	+++	NS	+++
	Zbtb16 (PLZF)	NS	NS	NS	+++	NS	+++	NS
Secretion proteins	Chgb	NS	NS	NS	NS	NS	NS	NS
	Gzmb (Granzyme B)	NS	+++	- - -	+++	NS	+++	NS
Enzymes	Cblb	NS	NS	NS	NS	NS	NS	-
	Entpd1 (CD39)	NS	+++	NS	+++	+++	++	+++
	Itk	NS	NS	NS	NS	NS	- -	+
	Nt5e (CD73)	NS	+++	++	+++	+++	NS	+++
	Serpinb6b	NS	++	NS	+++	++	NS	++
	Serpinb9	NS	NS	NS	+	+	NS	NS

+++	FC>4	(orange)	*Upregulated*
++	4>FC>2	(blue)	*Downregulated*
+	2>FC>1	(grey-green)	*scRNAseq concordant between BDC and Ins Tet+ cells*
NS	1>FC>-1	(yellow)	*scRNAseq non-concordant between BDC and Ins Tet+ cells*
-	-1>FC>-2	(light grey)	*Results concordant between TFH scRNAseq and TFH bulk RNAseq*
- -	-2>FC>-4	(lavender)	*Differences between TFH scRNAseq and TFH bulk RNAseq*
- - -	FC<-4	*NS*	*Non-significant*

Supplementary Table 4. Metal-labeled antibodies for Mass Cytometry

Label	Target	Clone	Company	Conc.(µg/ml)
114Cd	CD4	RM4-5	BD Biosciences	2.5
169Tm	PE	PE001	BioLegend	5
172Yb	CXCR3	CXCR3-173	ThermoFisher	5
165Ho	Biotin	1D4C5	Fluidigm	5
151Eu	CD28	37.51	Fluidigm	5
176Yb	CD278 (ICOS)	7E.17G9	Fluidigm	5
161Dy	CD134 (OX-40)	OX-86	BD Biosciences	5
143Nd	CD357 (GITR)	DTA1	Fluidigm	5
173Yb	CD152 (CTLA-4)	UC10-4B9	ThermoFisher	5
174Yb	CD223 (LAG-3)	C9B7W	Fluidigm	5
159Tb	CD279 (PD-1)	J43	Fluidigm	5
152Sm	TIGIT	GIG7	ThermoFisher	5
158Gd	IL-10	JES5-16E3	Fluidigm	5
160Gd	IL-21	FFA21	ThermoFisher	5
166Er	IL-4	11B11	Fluidigm	5
168Er	AHR	RPT9	ThermoFisher	5
154Sm	BATF	D7C5	Fluidigm	5
163Dy	BCL-6	K11291	Fluidigm	5
170Er	CEBPalpha	Rb Poly	ThermoFisher	5
116Cd	EGR2	EPR4004	Abcam	5
145Nd	FoxP3	FJK-16s	ThermoFisher	5
147Sm	ID2	E991	Abbaxa	5
149Sm	IRF1	EPR18301	Abcam	5
155Gd	IRF4	3E4	Fluidigm	5
146Nd	LEF1	EPR2029Y	Abcam	5
141Pr	cMaf	sym0F1	ThermoFisher	5
162Dy	NFIL3	1218A	R&D systems	5
175Lu	PRDM1 (Blimp1)	6D3	BD Biosciences	5
150Nd	STAT3	232209	R&D systems	5
156Gd	STAT4	2H9L5	ThermoFisher	5
153Eu	TBX21 (Tbet)	4B10	BioLegend	5
171Yb	phospho-ITK	A16064A	BioLegend	5

*Labeling was done using Maxpar X8 or MCP9 Antibody Labeling Kits (Fluidigm) except Fluidigm antibodies

Supplementary Table 5. Differential expression of 106 TR1/TFH/Treg-relevant genes in the Blimp1-dependent (TR1) and independent (TR1-like) vs. the TFH subcluster from pMHCII-NP-treated mice (10x scRNAseq)

Gene type	Gene name	TR1 vs TR1-like	TR1-like vs TFH	TR1 vs TFH
Cell adhesion molecules	Cd226	-	++	NS
	Itga2 (CD49b)	NS	NS	NS
	Itgae (CD103)	NS	NS	NS
	Ocln	NS	NS	NS
	S1pr2	NS	-	-
	Sell (CD62L)	+	NS	+
	Selplg (Psgl1)	+	+	+
Chemokyne receptors	Ccr5	+	NS	+
	Ccr7	-	+	+
	Cxcr3	NS	+	+
	Cxcr4	NS	-	-
	Cxcr5	-	-	--
Co-estimulatory molecules	Cd28	NS	NS	NS
	Cd40lg	-	NS	-
	Icos	+	+	+
	Klrk1 (NKG2D)	NS	NS	NS
	Sh2d1a (SAP)	NS	-	-
	Tnfrsf4 (Ox40)	NS	++	++
	Tnfrsf18 (GITR)	+	+	+
	Tnfsf4 (Ox40L)	NS	NS	NS
Co-inhibitory molecules	Ctla4	++	NS	++
	Fasl	NS	NS	NS
	Havcr2 (TIM-3)	+	NS	+
	Lag3	+	+	++
	Pdcd1 (PD-1)	-	--	--
	Tigit	NS	NS	NS
Cytokines	Ebi3 (IL27b)	NS	NS	NS
	Ifng	NS	+	+
	Il10	++	NS	++
	Il21	NS	+	+
	Il4	NS	-	-
	Il5	NS	NS	NS
	Mcub (Areg)	NS	NS	NS
	Tgfb1	NS	+	NS
Cytokine receptors	Il10ra	NS	NS	NS
	Il12rb2	NS	NS	NS
	Il21r	NS	NS	NS
	Il27ra	NS	NS	NS
	IL7r (CD127)	NS	NS	NS
	IL17rc	NS	NS	NS
	Il2ra (CD25)	NS	NS	+
	Tgfbr1	NS	NS	NS
	Tgfbr2	NS	-	-
	Tgfbr3	NS	NS	NS
	Atf6	NS	NS	NS
	Ahr	+	+	+
	Ajuba	NS	NS	NS
	Ascl2	NS	-	-
	Bach2	NS	NS	NS
	Batf	NS	+	NS
	Bcl6	-	-	--
	Bhlhe40	-	+	NS
	Bmyc	-	NS	NS
	Cbfa2t3	NS	-	-

Transcription factors	Cebpa	NS	-	-
	Dbp	NS	NS	NS
	E2f1	NS	NS	NS
	Egr2	NS	NS	NS
	Elk4	-	NS	-
	Eomes	NS	NS	NS
	FoxP1	NS	+	+
	FoxP3	NS	NS	NS
	Grhl1	NS	NS	NS
	Hmgb2	NS	NS	NS
	Id2	NS	+	+
	Id3	-	-	--
	Irf1	NS	+	+
	Irf4	NS	+	+
	Jdp2	NS	NS	NS
	Klf2	-	++	+
	Lef1	NS	-	-
	Lilrb4a	NS	NS	NS
	Maf	+	-	+
	Myb	NS	NS	NS
	Mybl2	NS	NS	NS
	Myc	NS	NS	NS
	Nfia	NS	-	-
	Nfil3	NS	NS	NS
	Nr1h3 (LXRα)	NS	NS	NS
	Pax5	NS	NS	NS
	Pax9	NS	NS	NS
	Pou2af1 (OcaB)	NS	-	-
	Prdm1 (Blimp-1)	++	NS	++
	Rbpj	NS	+	NS
	Runx2	NS	+	+
	Rora	NS	NS	NS
	S1pr1	NS	+	NS
	Six5	NS	NS	NS
	Sox4	NS	NS	NS
	Sox8	NS	NS	NS
	Stat1	-	+	NS
	Stat3	NS	NS	NS
	Stat4	-	NS	-
	Tbx21 (T-bet)	NS	NS	NS
	Tcf7	--	-	--
	Tox2	-	-	--
	Vdr	NS	NS	NS
	Zbtb16 (PLZF)	NS	NS	NS
Secretion proteins	Chgb	NS	NS	NS
	Gzmb (Granzyme B)	NS	NS	NS
Enzymes	Cblb	NS	NS	NS
	Entpd1 (CD39)	NS	NS	+
	Itk	NS	-	-
	Nt5e (CD73)	NS	NS	NS
	Serpinb6b	NS	NS	NS
	Serpinb9	NS	NS	NS

+++ FC>4
++ 4>FC>2
+ 2>FC>1
NS 1>FC>-1
- -1>FC>-2
- - -2>FC>-4
- - - FC<-4

(orange)	*Upregulated*
(blue)	*Downregulated*
NS	*Non-significant*

Suppl. Fig. 1

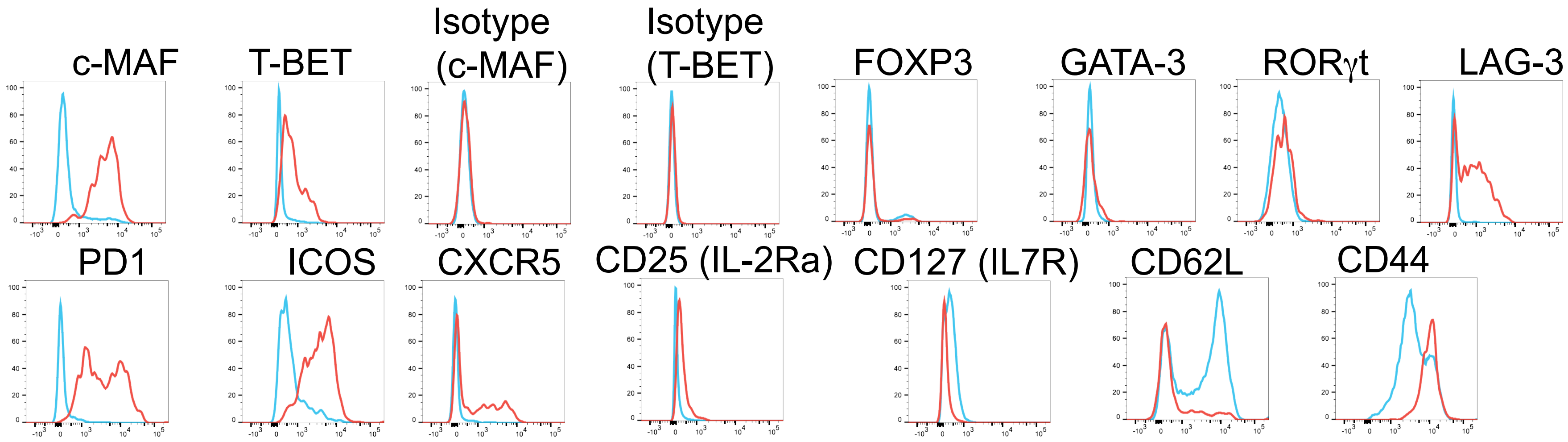

c-MAF
T-BET
Isotype (c-MAF)
Isotype (T-BET)
FOXP3
GATA-3
RORγt
LAG-3
PD1
ICOS
CXCR5
CD25 (IL-2Ra)
CD127 (IL7R)
CD62L
CD44

Supplementary Figure 1. Representative flow cytometry staining profiles of splenocytes from BDC2.5mi/IA^{g7}-NP-treated NOD mice (n=3 each from 3 experiments). The histogram overlays correspond to levels of expression of the different markers in the Tet^{+} (red) vs. Tet– (blue) CD4^{+}B220^{-} subsets.

Suppl. Fig. 2

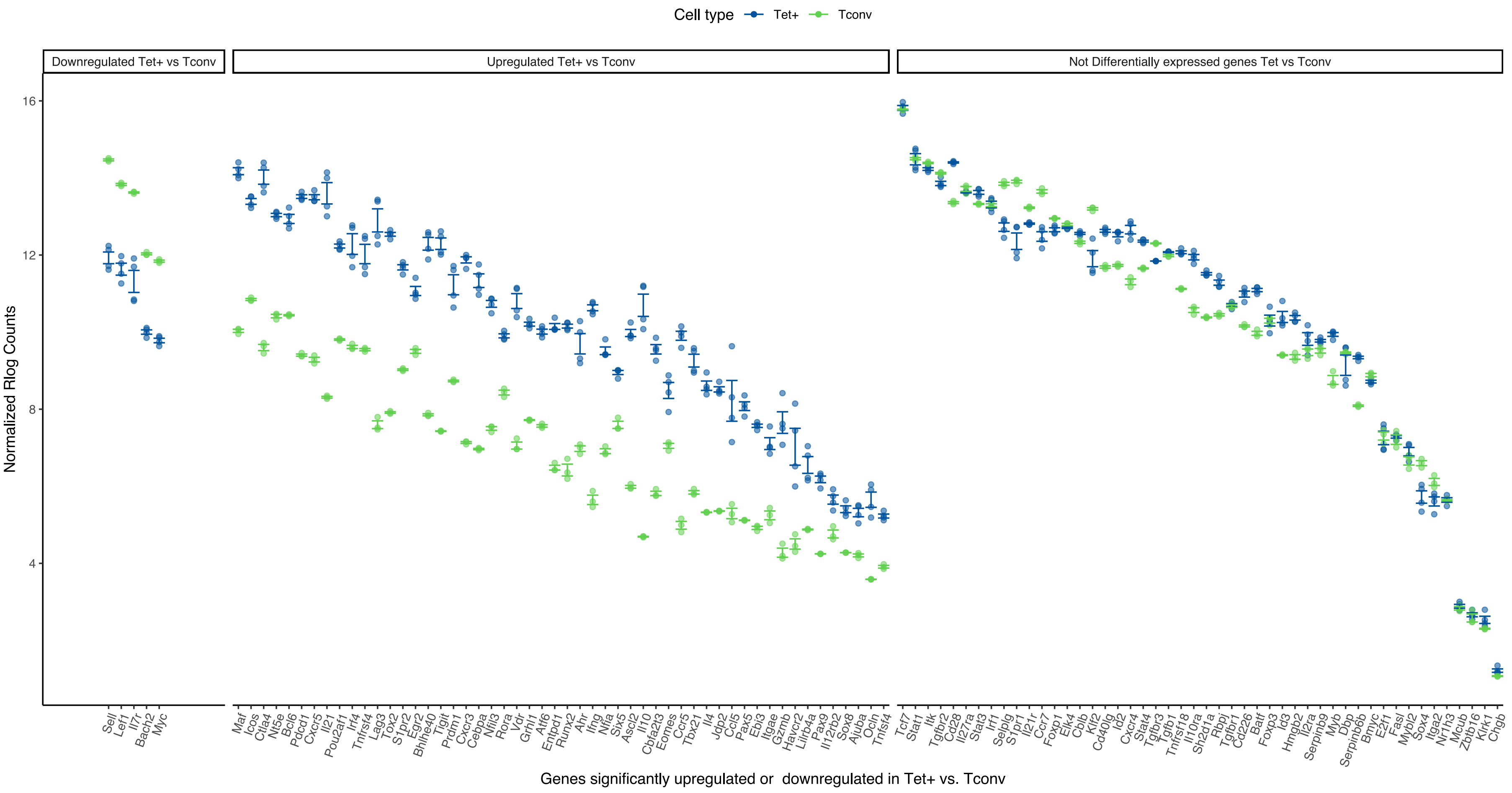

Cell type
Tet+
Tconv
Downregulated Tet+ vs Tconv
Upregulated Tet+ vs Tconv
Not Differentially expressed genes Tet vs Tconv
Normalized Rlog Counts
16
12
8
4
Sell Lef1 Il7r Bach2 Myc
Maf Icos Ctla4 Nt5e Bcl6 Pdcd1 Cxcr5 Il21 Pou2af1 Irf4 Tnfrsf4 Lag3 Tox2 S1pr2 Egr2 Bhlhe40 Tigit Prdm1 Cxcr3 Cebpa Nfil3 Rora Vdr Grhl1 Atf6 Entpd1 Runx2 Ahr Ifng Nfia Six5 Ascl2 Il10 Cbfa2t3 Eomes Ccr5 Tbx21 Il4 Jdp2 Ccl5 Pax5 Ebi3 Itgae Gzmb Havcr2 Lilrb4a Pax9 Il12rb2 Sox8 Ajuba Ocln Tnfsf4
Tcf7 Stat1 Itk Tgfbr2 Cd28 Il27ra Stat3 Irf1 Selplg S1pr1 Il21r Ccr7 Foxp1 Elk4 Cblb Klf2 Cd40lg Id2 Cxcr4 Stat4 Tgfbr3 Tgfb1 Tnfrsf18 Il10ra Sh2d1a Rbpj Tgfbr1 Cd226 Batf Foxp3 Id3 Hmgb2 Il2ra Serpinb9 Myb Dbp Serpinb6b Bmyc E2f1 Fasl Mybl2 Sox4 Itga2 Nr1h3 Mcub Zbtb16 Klrk1 Chgb
Genes significantly upregulated or downregulated in Tet+ vs. Tconv

Supplementary Figure 2. Normalized counts for 106 TR1/TFH/Treg-relevant genes in pMHCII-NP-induced TR1-like vs. Tconv CD4+ T-cells. Histogram of rlog normalized counts (normalization by counts per million (CPM)) of the 106 gene selection from **Supplementary Table 1** for BDC2.5mi/IA^{g7} Tet+ and Tconv subsets from the samples in **main Fig. 1A**. In blue, counts in Tet+ cells; in green, counts in Tconv cells. Genes are divided according to whether they are significantly upregulated or downregulated (or not differentially expressed) in Tet+ vs. Tconv cells (FC$\geq$4 or FC$\leq$-4, respectively, and FDR$\leq$0.01). Genes with <10 total counts (for all three replicates) are not plotted. Data correspond to the average $\pm$ SEM from 3 samples/cell type, from 1 experiment.

Suppl. Fig. 3

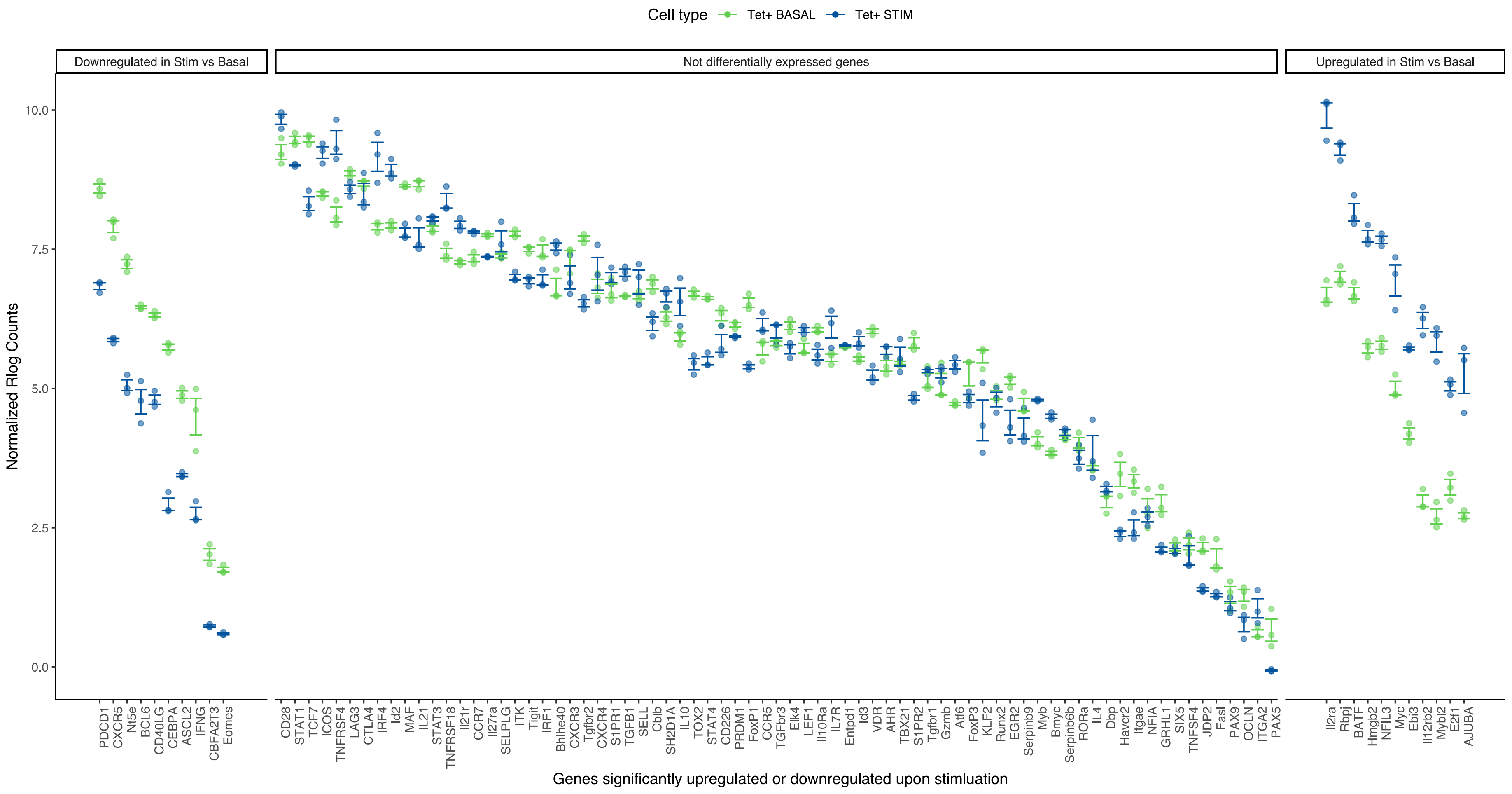

Cell type
Tet+ BASAL
Tet+ STIM
Downregulated in Stim vs Basal
Not differentially expressed genes
Upregulated in Stim vs Basal
Normalized Rlog Counts
10.0
7.5
5.0
2.5
0.0
PDCD1
CXCR5
Nt5e
BCL6
CD40LG
CEBPA
ASCL2
IFNG
CBFA2T3
Eomes
CD28
STAT1
TCF7
ICOS
TNFRSF4
LAG3
CTLA4
IRF4
Id2
MAF
IL21
STAT3
TNFRSF18
Il21r
CCR7
Il27ra
SELPLG
ITK
Tigit
IRF1
Bhlhe40
CXCR3
Tgfbr2
CXCR4
S1PR1
TGFB1
SELL
Cblb
SH2D1A
IL10
TOX2
STAT4
CD226
PRDM1
FoxP1
CCR5
TGFbr3
Elk4
LEF1
Il10Ra
IL7R
Entpd1
Id3
VDR
AHR
TBX21
S1PR2
Tgfbr1
Gzmb
Atf6
FoxP3
KLF2
Runx2
EGR2
Serpinb9
Myb
Bmyc
Serpinb6b
RORa
IL4
Dbp
Havcr2
Itgae
NFIA
GRHL1
SIX5
TNFSF4
JDP2
Fasl
PAX9
OCLN
ITGA2
PAX5
Il2ra
Rbpj
BATF
Hmgb2
NFIL3
Myc
Ebi3
Il12rb2
Mybl2
E2f1
AJUBA
Genes significantly upregulated or downregulated upon stimluation

Supplementary Figure 3. Changes in gene expression in pMHCII-NP-induced Tet+ cells upon stimulation *ex vivo*. Histogram of rlog normalized counts (CPM) of the 106 gene selection from **Supplementary Table 1** in basal and stimulated conditions from the Tet+ samples from main **Fig. 1A** (n=3/condition). In green and blue, counts in BDC2.5mi/IA^{g7} Tet+ cells in basal and stimulated conditions, respectively. Genes are divided according to whether they are significantly upregulated or downregulated (or not differentially expressed) upon stimulation (FC$\geq$4 or FC$\leq$-4, respectively, and FDR$\leq$0.01). Genes with <10 total counts (for all three replicates) are not plotted. Data correspond to the average $\pm$ SEM from 3 samples/condition, from 1 experiment.

Suppl. Fig. 4

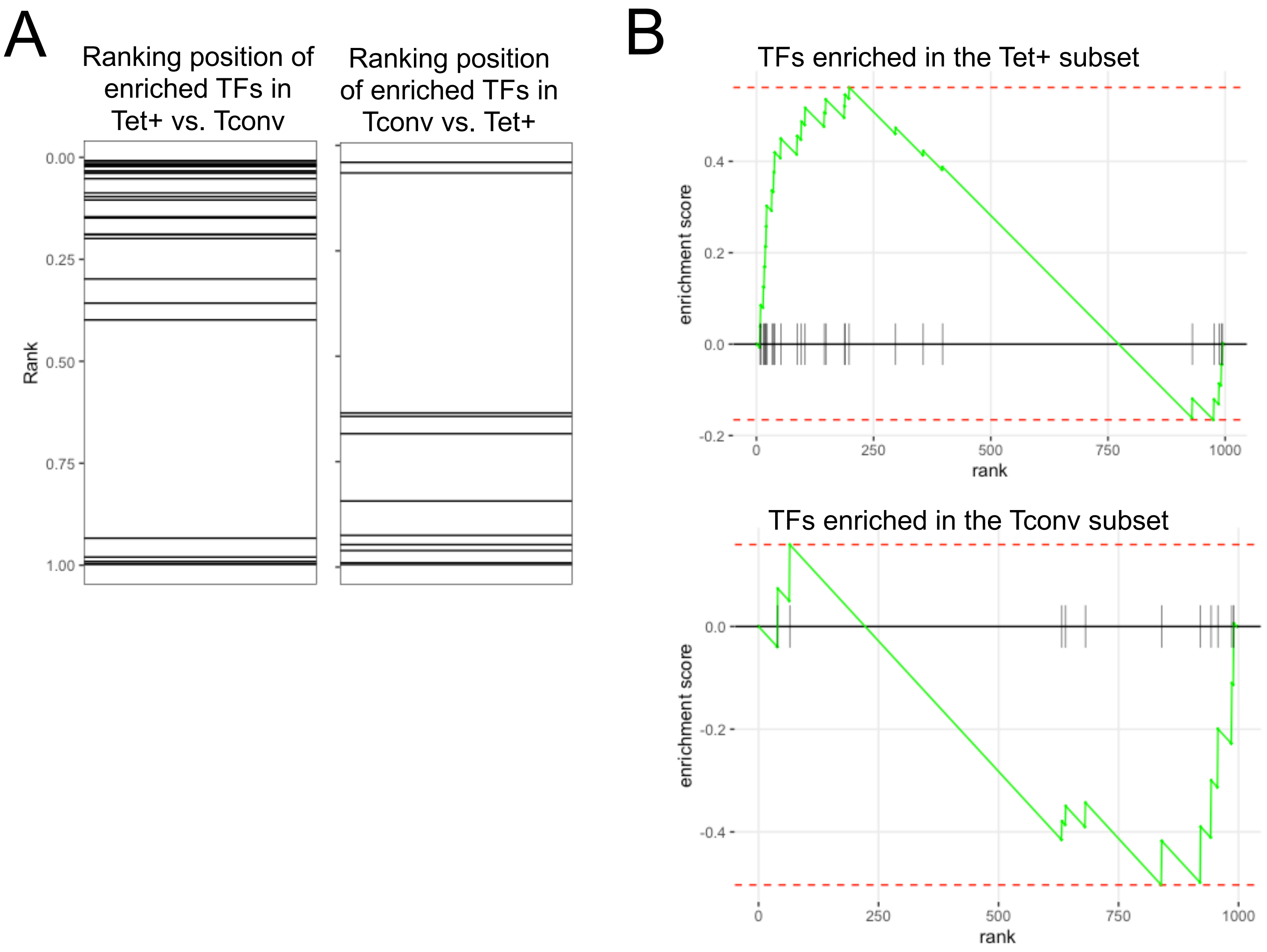

A
Ranking position of enriched TFs in Tet+ vs. Tconv
Ranking position of enriched TFs in Tconv vs. Tet+
Rank
0.00
0.25
0.50
0.75
1.00
B
TFs enriched in the Tet+ subset
enrichment score
0.4
0.2
0.0
-0.2
0
250
500
750
1000
rank
TFs enriched in the Tconv subset
enrichment score
0.0
-0.2
-0.4
0
250
500
750
1000
rank

Supplementary Figure 4. Transcription factor upregulation vs. gene expression. **A**, Validation transcription factors from **Supplementary Table 1** represented over a relative rank (from 0 to 1) of activity obtained from VIPER based on ARACNe regulon. More active transcription factors in BDC2.5mi/IA^{g7} Tet+ cells appear at the top of the list, while less active appear at the bottom. Left shows transcription factors that are described to be active and right, those that are expected to be inactive/less active in the Tet+ subset. **B**, Pre-ranked GSEA of transcription factors expected to be active (left) or inactive (right) in the BDC2.5mi/IA^{g7} Tet+ subset.

Suppl. Fig. 5

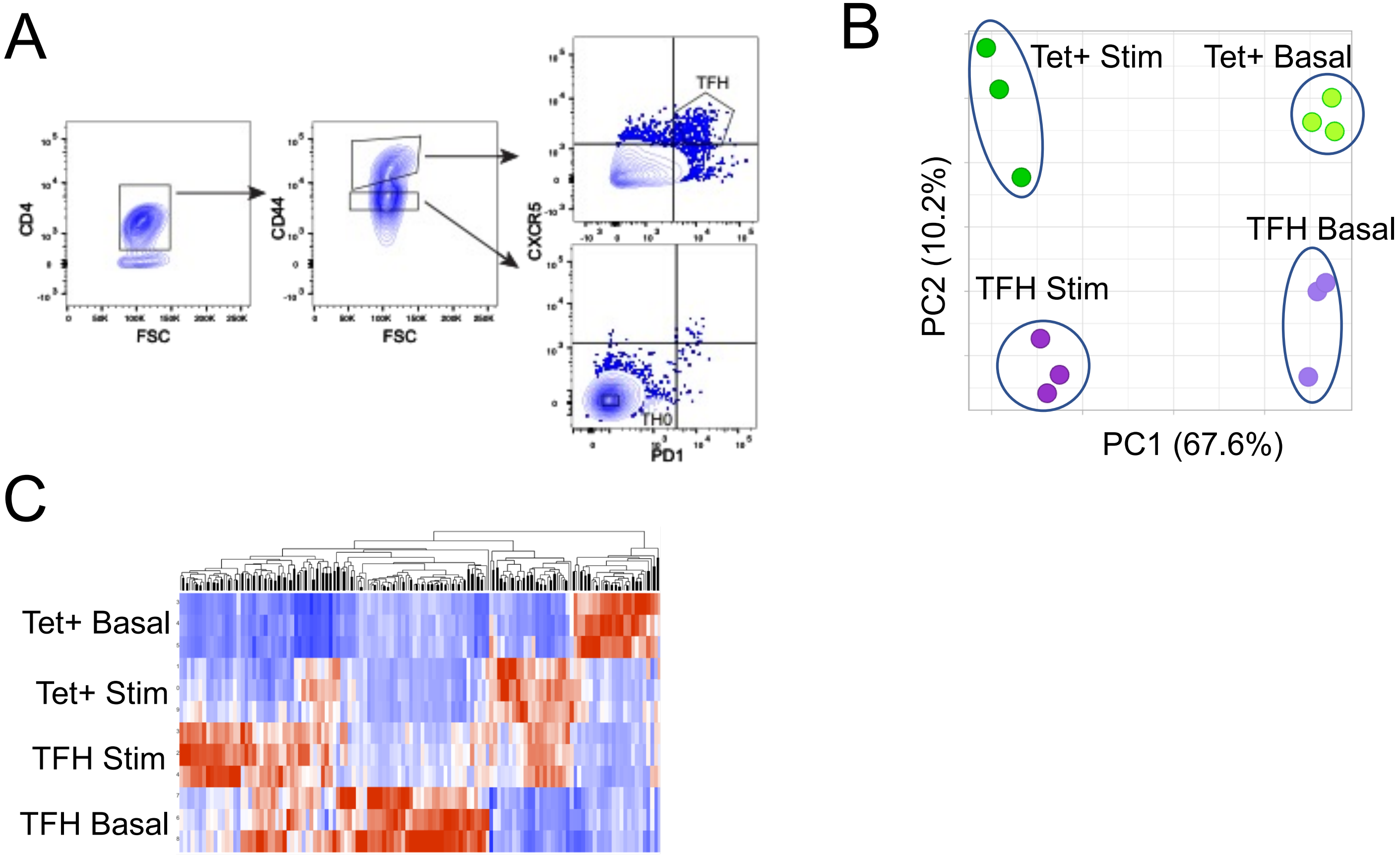
A
CD4
FSC
CD44
FSC
CXCR5
TFH
TH0
PD1
B
Tet+ Stim
Tet+ Basal
TFH Basal
TFH Stim
PC2 (10.2%)
PC1 (67.6%)
C
Tet+ Basal
Tet+ Stim
TFH Stim
TFH Basal

Supplementary Figure 5. pMHCII-NP-induced Tet+ cells vs. TFH cells. A, Gating strategy for TFH cell sorting for RNAseq. Three KLH-immunized NOD mice were processed in 2 batches (1 and 2 mice each, respectively) to obtain 3 samples of TFH cells ($CD4^{+}CD44^{hi}CXCR5^{hi}PD1^{hi}$) and Tconv cells ($CD4^{+}CD44^{-}CXCR5^{-}PD1^{-}$). **B**, PCA plot comparing basal and stimulated BDC2.5mi/IA^{g7} Tet+ and TF cells (light green and light purple vs. dark green and dark purple, respectively) (n=3 samples per cell type, from 1 experiment). **C,** Heatmap comparing differentially-expressed genes (|FC|$\geq$4 and FDR$\leq$0.01) for all 4 populations from **B**. In red, upregulated genes for the corresponding population; in blue, downregulated genes.

Suppl. Fig. 6

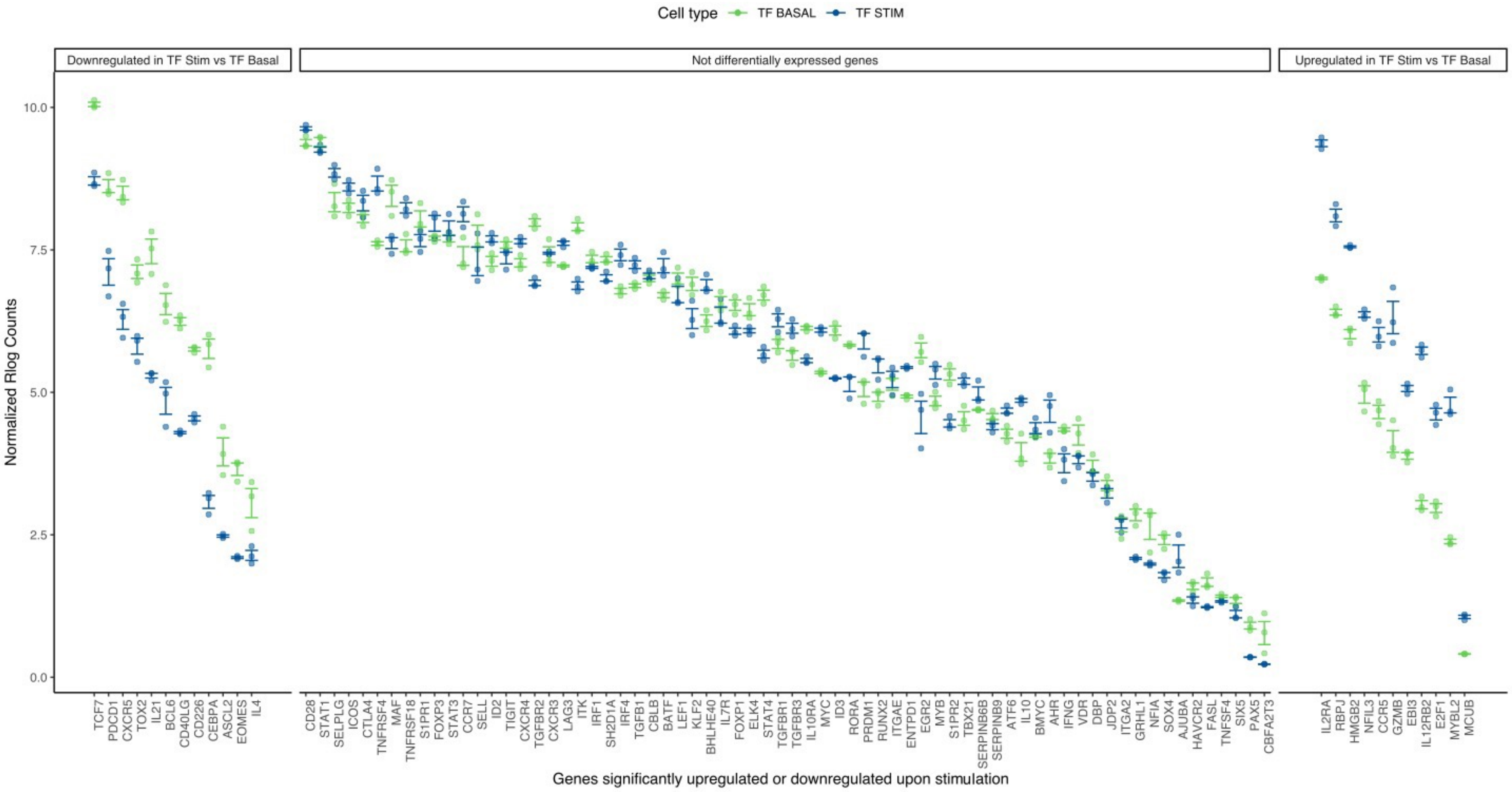

Cell type
TF BASAL
TF STIM
Downregulated in TF Stim vs TF Basal
Not differentially expressed genes
Upregulated in TF Stim vs TF Basal
Normalized Rlog Counts
10.0
7.5
5.0
2.5
0.0
TCF7 PDCD1 CXCR5 TOX2 IL21 BCL6 CD40LG CD226 CEBPA ASCL2 EOMES IL4
CD28 STAT1 SELPLG ICOS CTLA4 TNFRSF4 MAF TNFRSF18 S1PR1 FOXP3 STAT3 CCR7 SELL ID2 TIGIT CXCR4 TGFBR2 CXCR3 LAG3 ITK IRF1 SH2D1A IRF4 TGFB1 CBLB BATF LEF1 KLF2 BHLHE40 IL7R FOXP1 ELK4 STAT4 TGFBR1 TGFBR3 IL10RA MYC ID3 RORA PRDM1 RUNX2 ITGAE ENTPD1 EGR2 MYB S1PR2 TBX21 SERPINB6B SERPINB9 ATF6 IL10 BMYC AHR IFNG VDR DBP JDP2 ITGA2 GRHL1 NFIA SOX4 AJUBA HAVCR2 FASL TNFSF4 SIX5 PAX5 CBFA2T3
IL2RA RBPJ HMGB2 NFIL3 CCR5 GZMB EBI3 IL12RB2 E2F1 MYBL2 MCUB
Genes significantly upregulated or downregulated upon stimulation

Supplementary Figure 6. Changes in gene expression in endogenous TFH cells upon stimulation *ex vivo*. Histogram of rlog normalized counts (CPM) of the 106 gene selection from **Supplementary Table 1** in TFH cells before and after stimulation *ex vivo* with anti-CD3/anti-CD28 mAb-coated beads (n=3/condition). In green and blue, counts in TFH cells in basal and stimulated conditions, respectively. Genes are divided according to whether they are significantly upregulated or downregulated (or not differentially expressed) upon stimulation (FC$\geq$4 or FC$\leq$-4, respectively, and FDR$\leq$0.01). Genes with <10 total counts (for all three replicates) are not plotted. Data correspond to the average $\pm$ SEM from 3 samples/condition from 1 experiment.

Suppl. Fig. 7

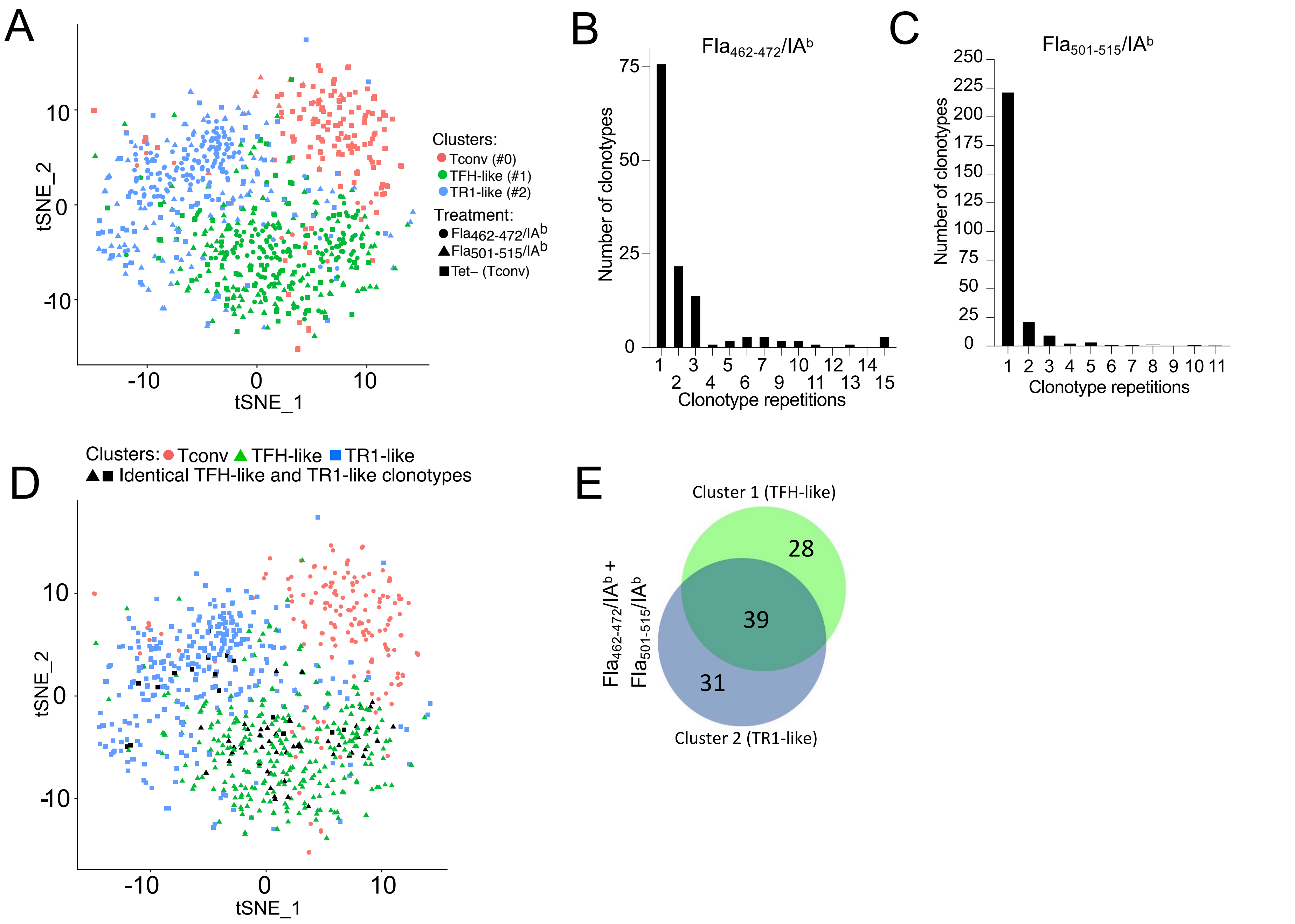

A
tSNE_2
tSNE_1
Clusters:
Tconv (#0)
TFH-like (#1)
TR1-like (#2)
Treatment:
Fla462-472/IAb
Fla501-515/IAb
Tet– (Tconv)
B
Fla462-472/IAb
Number of clonotypes
Clonotype repetitions
C
Fla501-515/IAb
Number of clonotypes
Clonotype repetitions
D
Clusters: Tconv TFH-like TR1-like
Identical TFH-like and TR1-like clonotypes
tSNE_2
tSNE_1
E
Cluster 1 (TFH-like)
Cluster 2 (TR1-like)
Fla462-472/IAb +
Fla501-515/IAb
28
39
31

Supplementary Figure 7. pMHCII-NP-induced TR1-like cells are transcriptionally homogeneous but oligoclonal and co-exist with a Tet$^+$ TFH-like subpopulation that contains identical clonotypes. A, Seurat clustering analysis of the Tet$^+$ pools from $Fla_{462-472}/IA^b$- and $Fla_{501-515}/IA^b$-NP-treated C57BL/6 mice (n=6 and 5 mice, respectively, from 2 experiments). Data correspond to Smartseq2-based scRNAseq data for sorted Tet$^+$ and Tet$^-$ cells. **B-C**, Distribution of unique TCR sequences in the Tet$^+$ pools arising in response to treatment with $Fla_{462-472}/IA^b$- or $Fla_{501-515}/IA^b$-NPs in C57BL/6 mice, respectively, from 1 experiment each. The histogram plot shows the distribution of the different TCRαβ clonotypes identified vs. the number of cells (clones) expressing each TCRαβ pair. **D,** tSNE plot from **A** showing the cluster location for TCRαβ pairs expressed by more than one cell. Data correspond to $Fla_{462-472}/IA^b$- or $Fla_{501-515}/IA^b$-NP-treated C57BL/6 mice. **E**, Venn diagram from **D** showing the distribution of repeated TCRαβ pairs in clusters 1 vs. 2. Most (39/67) of the clonotypes found in the TFH-like cluster (#1) were also found in the TR1-like cluster (#2) (39/70).

Suppl. Fig. 8

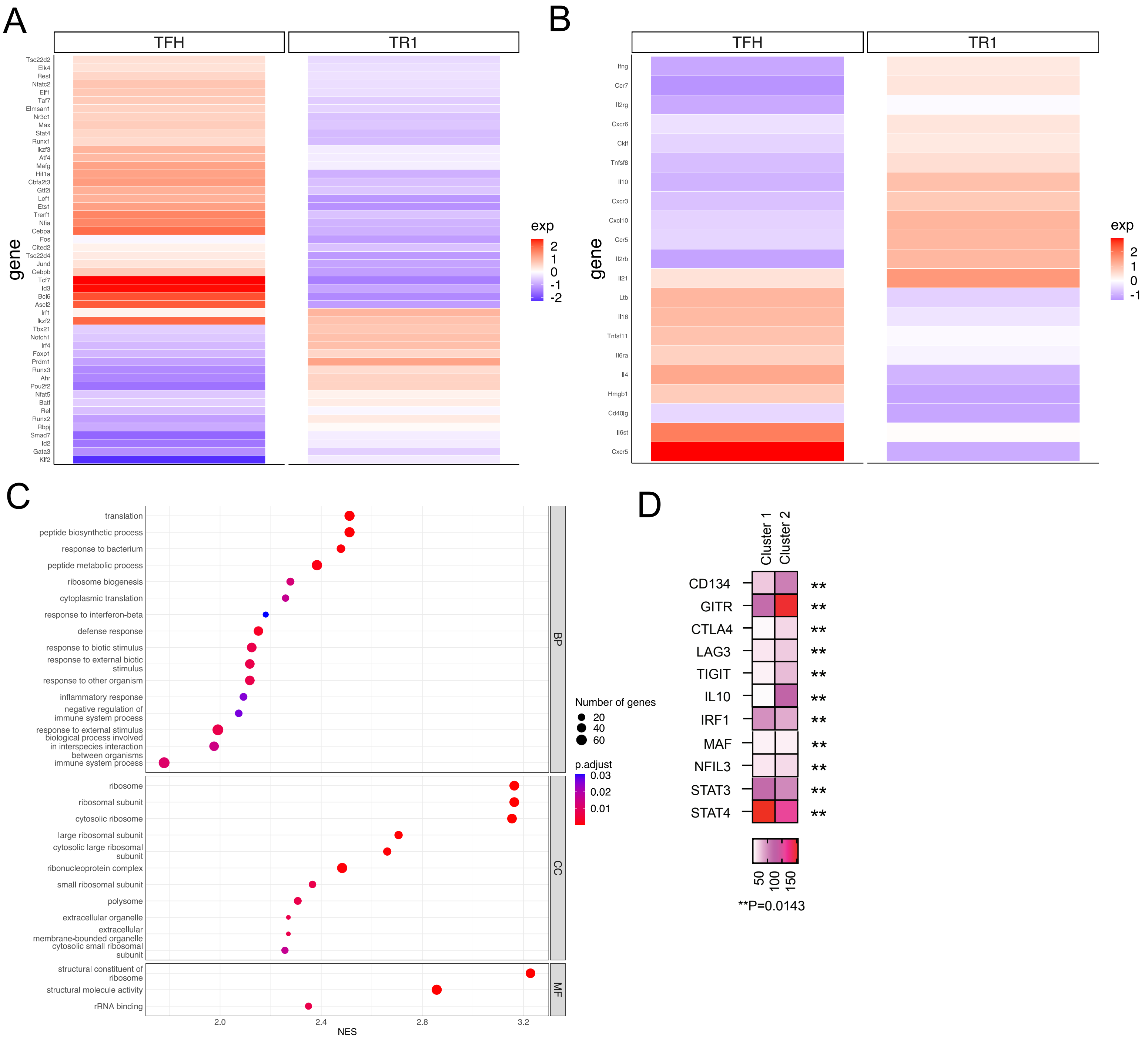
A
TFH
TR1
gene
Tsc22d2
Elk4
Rest
Nfatc2
Elf1
Taf7
Elmsan1
Nr3c1
Max
Stat4
Runx1
Ikzf3
Atf4
Mafg
Hif1a
Cbfa2t3
Gtf2i
Lef1
Ets1
Trerf1
Nfia
Cebpa
Fos
Cited2
Tsc22d4
Jund
Cebpb
Tcf7
Id3
Bcl6
Ascl2
Irf1
Ikzf2
Tbx21
Notch1
Irf4
Foxp1
Prdm1
Runx3
Ahr
Pou2f2
Nfat5
Batf
Rel
Runx2
Rbpj
Smad7
Id2
Gata3
Klf2
exp
2
1
0
-1
-2
B
TFH
TR1
gene
Ifng
Ccr7
Il2rg
Cxcr6
Cklf
Tnfsf8
Il10
Cxcr3
Cxcl10
Ccr5
Il2rb
Il21
Ltb
Il16
Tnfsf11
Il6ra
Il4
Hmgb1
Cd40lg
Il6st
Cxcr5
exp
2
1
0
-1
C
translation
peptide biosynthetic process
response to bacterium
peptide metabolic process
ribosome biogenesis
cytoplasmic translation
response to interferon-beta
defense response
response to biotic stimulus
response to external biotic stimulus
response to other organism
inflammatory response
negative regulation of immune system process
response to external stimulus
biological process involved in interspecies interaction between organisms
immune system process
BP
ribosome
ribosomal subunit
cytosolic ribosome
large ribosomal subunit
cytosolic large ribosomal subunit
ribonucleoprotein complex
small ribosomal subunit
polysome
extracellular organelle
extracellular membrane-bounded organelle
cytosolic small ribosomal subunit
CC
structural constituent of ribosome
structural molecule activity
rRNA binding
MF
2.0
2.4
2.8
3.2
NES
Number of genes
20
40
60
p.adjust
0.03
0.02
0.01
D
Cluster 1
Cluster 2
CD134 **
GITR **
CTLA4 **
LAG3 **
TIGIT **
IL10 **
IRF1 **
MAF **
NFIL3 **
STAT3 **
STAT4 **
50
100
150
**P=0.0143

Supplementary Figure 8. Differential expression of transcription factor, cytokines/chemokine and cytokine/chemokine receptor genes, and gene ontology pathways between the Tet^{+} TFH-like and Tet^{+} TR1-like/TR1 clusters. **A-B,** Heatmaps showing scaled average gene expression for differentially expressed (|FC|>2 and FDR<0.05) transcription factor (GO:0003700) (**A**) and cytokine/chemokine and cytokine/chemokine receptor genes (GO:0005125, GO:0004896, GO:0019956) (**B**) in the TFH- and TR1-like/TR1 cell sub-clusters found within the BDC2.5/IAg7-NP-induced tetramer+ cell pool. Data were obtained from 10x genomics-based scRNAseq data for sorted Tet+ and Tet– cells from NOD mice treated with BDC2.5mi/IA^{g7}-NP (from n=5 mice, from 1 experiment). **C**, Dot plot showing the differentially enriched gene ontology (GO) pathways (adjusted P value < 0.05) for differentially expressed genes (|FC|>2 and FDR<0.05) between the TFH- and TR1-like/TR1 sub-clusters from **A-B**. Pathways are ordered based on the normalized enrichment score. Color represents the value of the adjusted P value. Dot size represents the number of genes found for each pathway. Pathways are classified based on their ontology: BP: Biological process; CC: Celular Components; and MF: Molecular function. **D**, Most differentially expressed markers (P<0.02) between the TFH- and TR1-like/TR1 cell sub-clusters (#1 and #2, respectively), as determined via mass cytometry (n=4 mice, from 1 experiment).

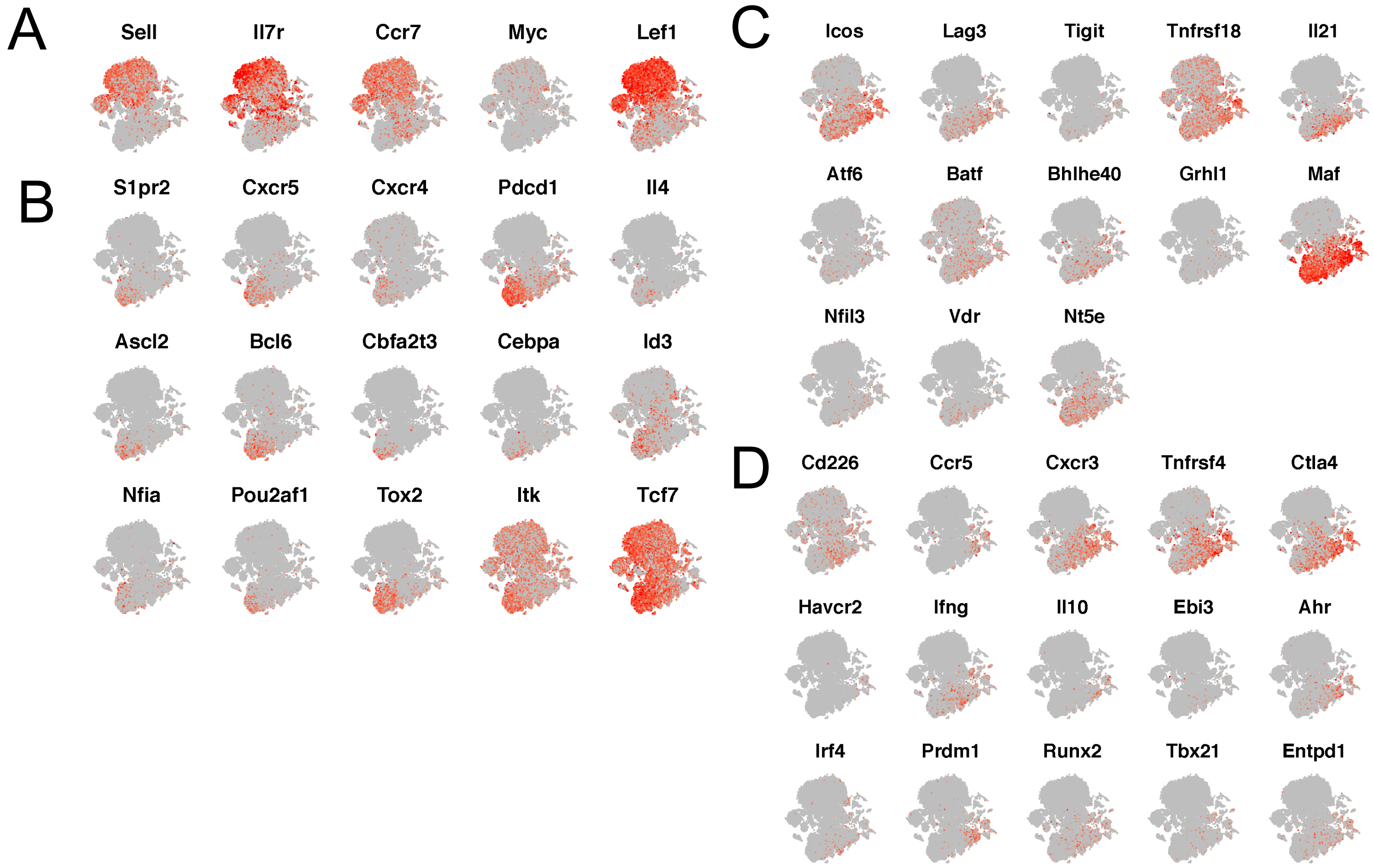
A
Sell
Il7r
Ccr7
Myc
Lef1
B
S1pr2
Cxcr5
Cxcr4
Pdcd1
Il4
Ascl2
Bcl6
Cbfa2t3
Cebpa
Id3
Nfia
Pou2af1
Tox2
Itk
Tcf7
C
Icos
Lag3
Tigit
Tnfrsf18
Il21
Atf6
Batf
Bhlhe40
Grhl1
Maf
Nfil3
Vdr
Nt5e
D
Cd226
Ccr5
Cxcr3
Tnfrsf4
Ctla4
Havcr2
Ifng
Il10
Ebi3
Ahr
Irf4
Prdm1
Runx2
Tbx21
Entpd1

Supplementary Figure 9. **Differences in gene expression for representative genes in the cell clusters identified via 10x genomics scRNAseq**. **A**, enriched in Tconv cells from main **Fig. 3**. **B**, enriched in the BDC2.5mi/IA^{g7}/InsB$_{9-23}$/IA^{g7} Tet+ TFH-like sub-cluster from **Fig. 3**. **C**, shared between the Tet+ TFH- and Tet+ TR1-like sub-clusters from **Fig. 3**. **D**, enriched in the Tet+ TR1-like sub-cluster from **Fig. 3**.

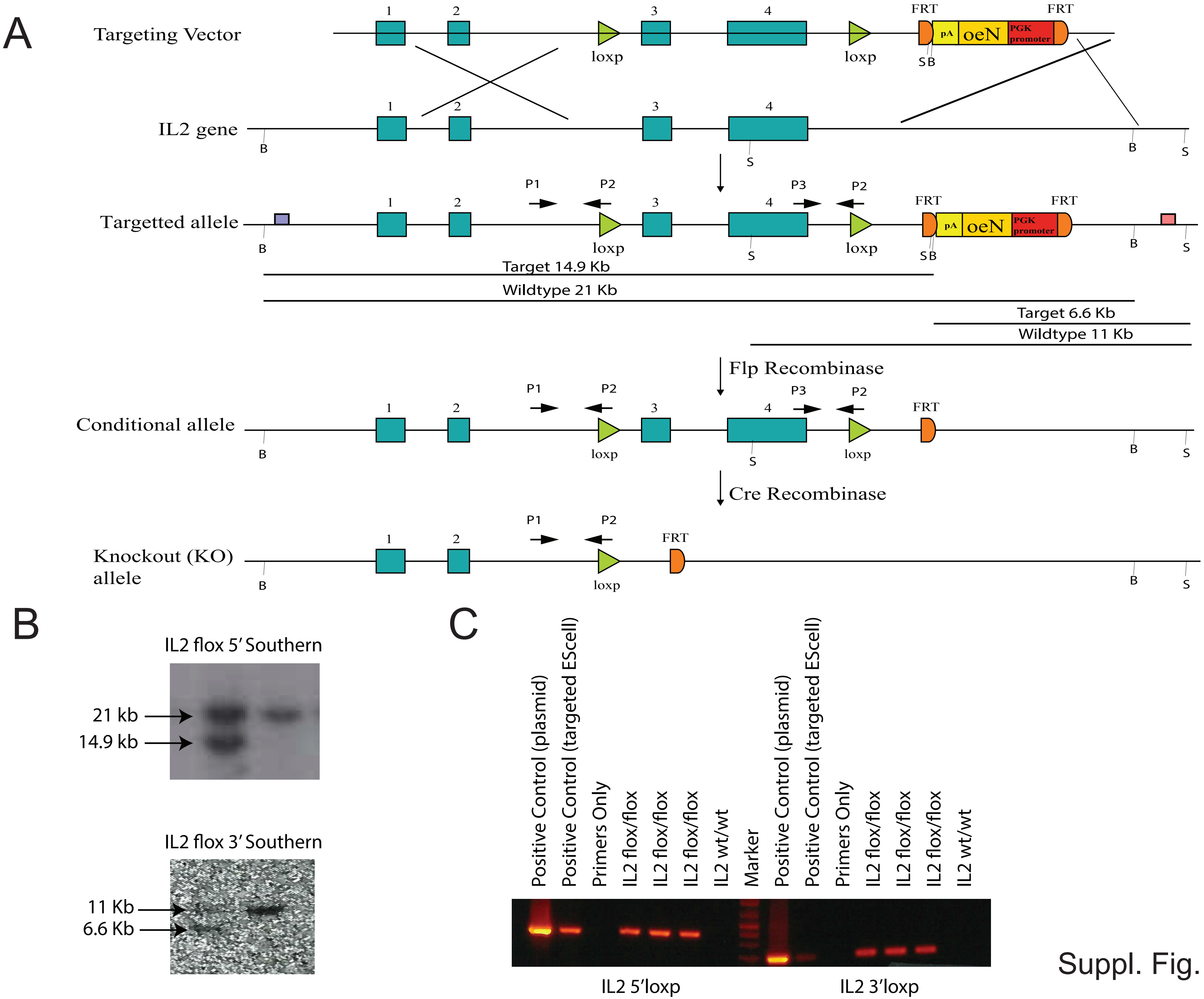
A
Targeting Vector
1
2
loxp
3
4
loxp
FRT
pA
oeN
PGK promoter
FRT
S B
IL2 gene
B
S
B
S
P1
P2
P3
P2
Targetted allele
loxp
loxp
Target 14.9 Kb
Wildtype 21 Kb
Target 6.6 Kb
Wildtype 11 Kb
Flp Recombinase
Conditional allele
Cre Recombinase
Knockout (KO) allele
B
IL2 flox 5′ Southern
21 kb
14.9 kb
IL2 flox 3′ Southern
11 Kb
6.6 Kb
C
Positive Control (plasmid)
Positive Control (targeted EScell)
Primers Only
IL2 flox/flox
IL2 flox/flox
IL2 flox/flox
IL2 wt/wt
Marker
Positive Control (plasmid)
Positive Control (targeted EScell)
Primers Only
IL2 flox/flox
IL2 flox/flox
IL2 flox/flox
IL2 wt/wt
IL2 5′loxp
IL2 3′loxp

Suppl. Fig. 10

Supplementary Figure 10. **Production of a conditional gene-targeted NOD.*Il2*^{loxP} strain**. **A,** Targeting strategy in embryonic stem cells and location of primers and probes for genotyping. **B-C**, Southern blotting (**B**) and PCR (**C**) documenting the presence of the loxP sites downstream of exon 3 and upstream of exon 4 of *Il2*.

Suppl. Fig. 11

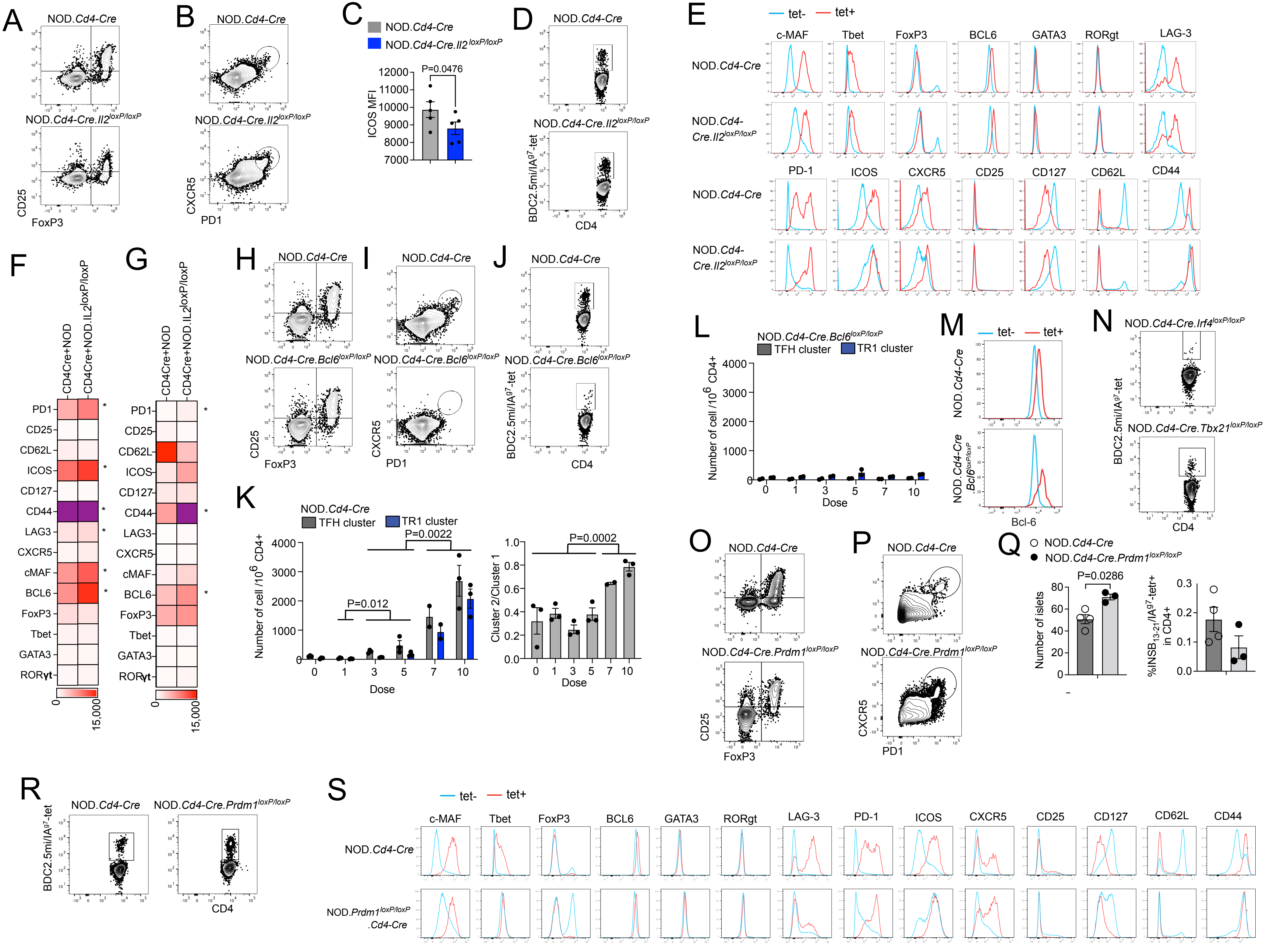

A
NOD.Cd4-Cre
NOD.Cd4-Cre.Il2loxP/loxP
CD25
FoxP3
B
NOD.Cd4-Cre
NOD.Cd4-Cre.Il2loxP/loxP
CXCR5
PD1
C
NOD.Cd4-Cre
NOD.Cd4-Cre.Il2loxP/loxP
P=0.0476
ICOS MFI
12000
11000
10000
9000
8000
7000
D
NOD.Cd4-Cre
NOD.Cd4-Cre.Il2loxP/loxP
BDC2.5mi/IAg7-tet
CD4
E
tet-
tet+
c-MAF
Tbet
FoxP3
BCL6
GATA3
RORgt
LAG-3
NOD.Cd4-Cre
NOD.Cd4-Cre.Il2loxP/loxP
PD-1
ICOS
CXCR5
CD25
CD127
CD62L
CD44
F
CD4Cre+NOD
CD4Cre+NOD.IL2loxP/loxP
PD1
CD25
CD62L
ICOS
CD127
CD44
LAG3
CXCR5
cMAF
BCL6
FoxP3
Tbet
GATA3
RORγt
0
15,000
G
CD4Cre+NOD
CD4Cre+NOD.IL2loxP/loxP
PD1
CD25
CD62L
ICOS
CD127
CD44
LAG3
CXCR5
cMAF
BCL6
FoxP3
Tbet
GATA3
RORγt
0
15,000
H
NOD.Cd4-Cre
NOD.Cd4-Cre.Bcl6loxP/loxP
CD25
FoxP3
I
NOD.Cd4-Cre
NOD.Cd4-Cre.Bcl6loxP/loxP
CXCR5
PD1
J
NOD.Cd4-Cre
NOD.Cd4-Cre.Bcl6loxP/loxP
BDC2.5mi/IAg7-tet
CD4
K
NOD.Cd4-Cre
TFH cluster
TR1 cluster
P=0.0022
P=0.012
Number of cell /10^6 CD4+
4000
3000
2000
1000
0
0
1
3
5
7
10
Dose
P=0.0002
Cluster 2/Cluster 1
1.0
0.8
0.6
0.4
0.2
0.0
Dose
L
NOD.Cd4-Cre.Bcl6loxP/loxP
TFH cluster
TR1 cluster
Number of cell /10^6 CD4+
4000
3000
2000
1000
0
Dose
M
tet-
tet+
NOD.Cd4-Cre
NOD.Cd4-Cre.Bcl6loxP/loxP
Bcl-6
N
NOD.Cd4-Cre.Irf4loxP/loxP
NOD.Cd4-Cre.Tbx21loxP/loxP
BDC2.5mi/IAg7-tet
CD4
O
NOD.Cd4-Cre
NOD.Cd4-Cre.Prdm1loxP/loxP
CD25
FoxP3
P
NOD.Cd4-Cre
NOD.Cd4-Cre.Prdm1loxP/loxP
CXCR5
PD1
Q
NOD.Cd4-Cre
NOD.Cd4-Cre.Prdm1loxP/loxP
P=0.0286
Number of islets
80
60
40
20
0
%INSB13-21/IAg7-tetr+ in CD4+
0.3
0.2
0.1
0.0
R
NOD.Cd4-Cre
NOD.Cd4-Cre.Prdm1loxP/loxP
BDC2.5mi/IAg7-tet
CD4
S
tet-
tet+
c-MAF
Tbet
FoxP3
BCL6
GATA3
RORgt
LAG-3
PD-1
ICOS
CXCR5
CD25
CD127
CD62L
CD44
NOD.Cd4-Cre
NOD.Prdm1loxP/loxP.Cd4-Cre

Supplementary Figure 11. Representative flow cytometry profiles for FoxP3+ Tregs and TFH cells in conditional knockout mice from Figures 4 and 5. A, Representative staining profiles for $CD25^+FoxP3^+$ CD4+ T-cells in NOD.*Cd4-Cre.Il2*$^{loxP/loxP}$ and NOD.*Cd4-Cre* mice. **B**, Representative staining profiles for endogenous PD-1^{high}/$CXCR5^{high}$ cells in splenocytes from NOD.*Cd4-Cre.Il2*$^{loxP/loxP}$ and NOD.*Cd4-Cre* mice. **C**, Mean fluorescence intensity for splenic PD-1^{high}/$CXCR5^{high}$ cells of NOD.*Cd4-Cre.Il2*$^{loxP/loxP}$ and NOD.*Cd4-Cre* mice. Data correspond to 5 mice per strain, from 2 experiments. **D,** Representative flow cytometry profiles for BDC2.5mi/IA^{g7} Tet^+ CD4+ T-cells from the spleen of BDC2.5mi/IA^{g7}-NP-treated NOD.*Cd4-Cre.Il2*$^{loxP/loxP}$ and NOD.*Cd4-Cre* mice. **E**, Representative FACS staining profiles for TR1-associated and -non-associated cell markers in the BDC2.5mi/IA^{g7} Tet^- and Tet^+ CD4+ T-cells of the mice from main Fig. 4 panel **E**. **F and G**, heatmaps comparing the mean fluorescence intensity for the markers from panel **E** in Tet– and Tet+ cells, respectively. **H,** Representative staining profiles for $CD25^+FoxP3^+$ CD4+ T-cells in NOD.*Cd4-Cre.Bcl6*$^{loxP/loxP}$ and NOD.*Cd4-Cre* mice. **I**, Representative staining profiles for PD-1^{high}/$CXCR5^{high}$ (TFH) cells in the spleens of NOD.*Cd4-Cre.Bcl6*$^{loxP/loxP}$ and NOD.*Cd4-Cre* mice. **J**, Representative flow cytometry profiles for BDC2.5mi/IA^{g7} Tet^+ CD4+ T-cells from the spleen of BDC2.5mi/IA^{g7}-NP-treated NOD.*Cd4-Cre.Bcl6*$^{loxP/loxP}$ and NOD.*Cd4-Cre* mice. **K,** Changes in the absolute numbers of BDC2.5mi/IA^{g7} Tet+ TFH- and TR1-like cells (left) or cluster 2 (TR1):cluster 1 (TFH) ratios (right) in NOD.*Cd4-Cre* mice as a function of BDC2.5mi/IA^{g7}-NP dose numbers, as measured by mass cytometry. Data correspond to 3 mice per dose number, except the 7-dose group (n=2), from 3 experiments. **L,** Changes in the absolute numbers of BDC2.5mi/IA^{g7} Tet+ TFH- and TR1-like cells in NOD.*Cd4-Cre.Bcl6*$^{loxP/loxP}$ mice as a function of BDC2.5mi/IA^{g7}-NP dose numbers. Data correspond to 2 mice per dose number, from 3 experiments. **M,** Representative BCL-6 staining profile for Tet+ vs. Tet– cells of BDC2.5mi/IA^{g7}-NP-treated NOD.*Cd4-Cre.Bcl6*$^{loxP/loxP}$ and NOD.*Cd4-Cre* mice. **N**, Representative flow cytometry profiles for BDC2.5mi/IA^{g7} Tet^+ CD4+ T-cells from the spleen of BDC2.5mi/IA^{g7}-NP-treated NOD.*Cd4-Cre.Irf4*$^{loxP/loxP}$ and NOD.*Cd4-Cre.Tbx21*$^{loxP/loxP}$ mice. **O,** Representative flow cytometry profiles for $CD25^+FoxP3^+$ CD4+ T-cells in NOD.*Cd4-Cre.Prdm1*$^{loxP/loxP}$

and NOD.*Cd4-Cre* mice. **P**, Representative flow cytometry profiles for PD-1^{high}/$CXCR5^{high}$ (TFH) cells in the spleens of NOD.*Cd4-Cre.Prdm1*$^{loxP/loxP}$ and NOD.*Cd4-Cre* mice. **Q,** Left, average number (± SEM) of isolated islets in pancreata from NOD.*Cd4-Cre.Prdm1*$^{loxP/loxP}$ and NOD.*Cd4-Cre* mice. Right, average percentage (± SEM) of $InsB_{13-21}$/IA^{g7}-specific CD4+ T-cells in islets from NOD.*Cd4-Cre.Prdm1*$^{loxP/loxP}$ and NOD.*Cd4-Cre* mice. Data correspond to n=3 and n=4 mice, respectively. P values were calculated via Mann-Whitney U. **R**, Representative flow cytometry profiles for BDC2.5mi/IA^{g7} Tet^{+} CD4+ T-cells from the spleen of BDC2.5mi/IA^{g7}-NP-treated NOD.*Cd4-Cre.Prdm1*$^{loxP/loxP}$ and NOD.*Cd4-Cre* mice. **S**, Representative flow cytometry profiles for TR1-associated and -non-associated cell markers in the BDC2.5mi/IA^{g7} Tet^{-} and Tet^{+} CD4+ T-cells of the mice from main Fig. 5 panel **L**.

Suppl. Fig. 12

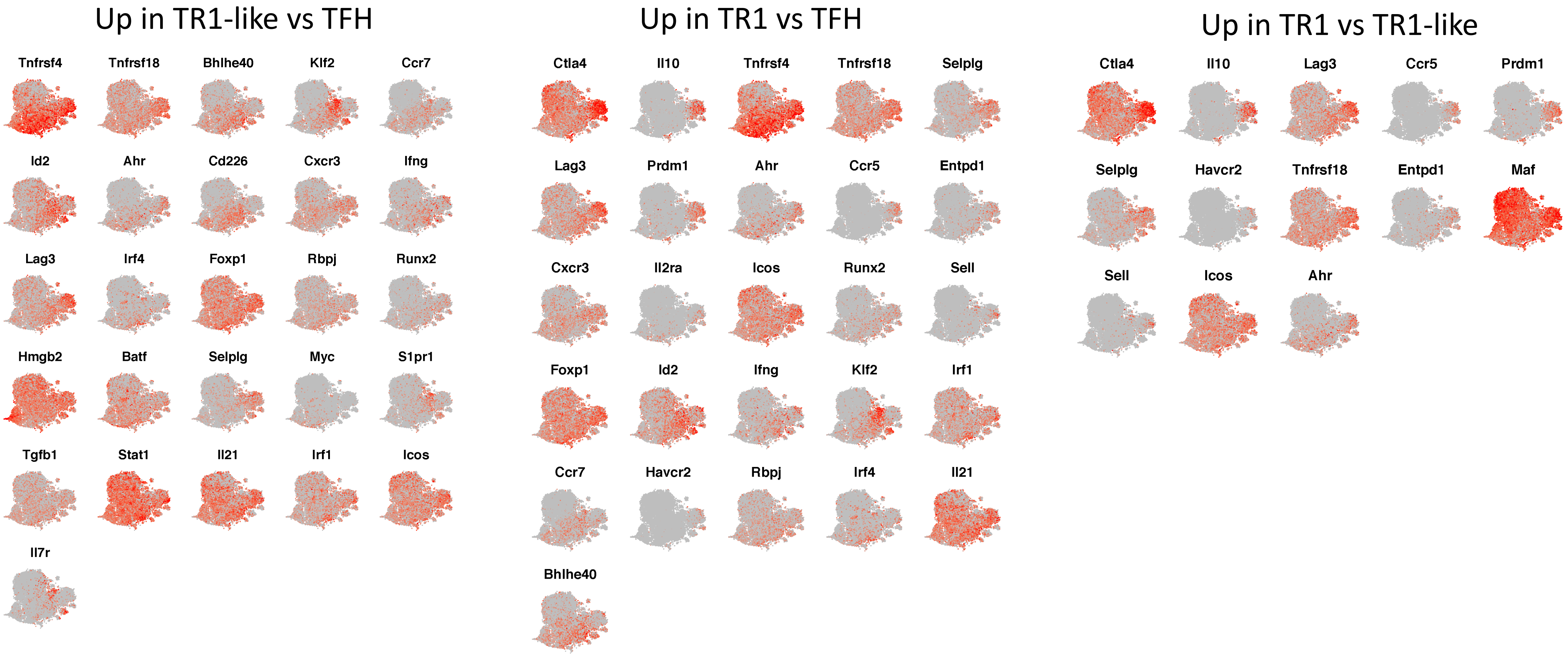
Up in TR1-like vs TFH
Tnfrsf4
Tnfrsf18
Bhlhe40
Klf2
Ccr7
Id2
Ahr
Cd226
Cxcr3
Ifng
Lag3
Irf4
Foxp1
Rbpj
Runx2
Hmgb2
Batf
Selplg
Myc
S1pr1
Tgfb1
Stat1
Il21
Irf1
Icos
Il7r
Up in TR1 vs TFH
Ctla4
Il10
Tnfrsf4
Tnfrsf18
Selplg
Lag3
Prdm1
Ahr
Ccr5
Entpd1
Cxcr3
Il2ra
Icos
Runx2
Sell
Foxp1
Id2
Ifng
Klf2
Irf1
Ccr7
Havcr2
Rbpj
Irf4
Il21
Bhlhe40
Up in TR1 vs TR1-like
Ctla4
Il10
Lag3
Ccr5
Prdm1
Selplg
Havcr2
Tnfrsf18
Entpd1
Maf
Sell
Icos
Ahr

Supplementary Figure 12. **Differences in gene expression for representative genes in Tet+ TFH, transitional TR1-like and terminally differentiated TR1 clusters identified via 10x genomics scRNAseq**. Feature plots correspond to the Tet+ cells isolated from NOD.*Cd4-Cre* and NOD.*Prdm1*$^{loxP/loxP}$ mice from main Fig. 6A. Only genes listed in Supplementary Table 1 are shown. **Left**, specifically enriched in Tet+ TR1-like vs. Tet+ TFH. **Middle**, specifically enriched in terminally differentiated Tet+ TR1 vs. Tet+ TFH. **Right**, specifically enriched in terminally differentiated Tet+ TR1 vs. transitional Tet+ TR1-like.

Suppl. Fig. 13

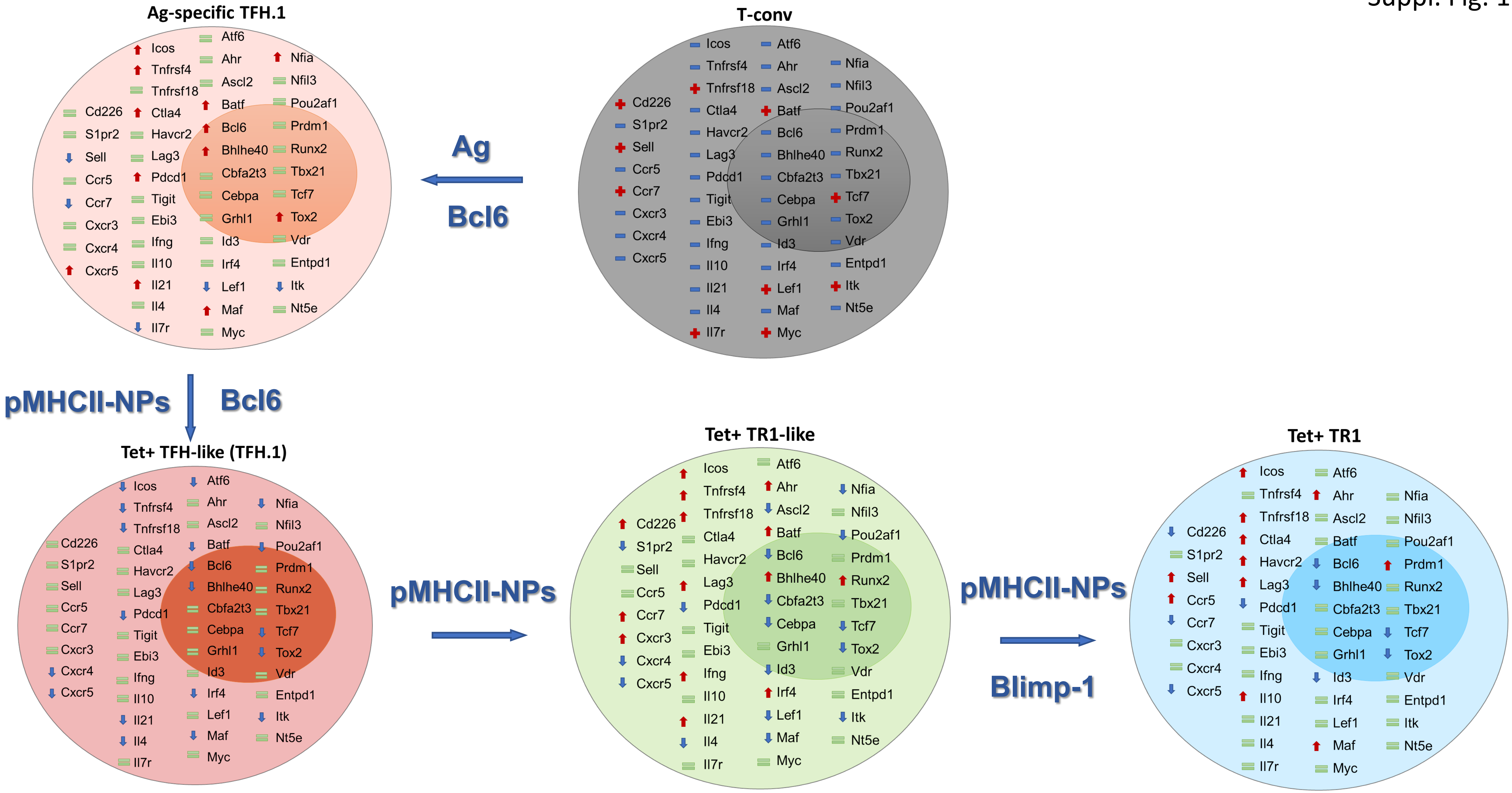
Ag-specific TFH.1
T-conv
Ag
Bcl6
pMHCII-NPs
Bcl6
Tet+ TFH-like (TFH.1)
pMHCII-NPs
Tet+ TR1-like
pMHCII-NPs
Blimp-1
Tet+ TR1
Icos
Tnfrsf4
Tnfrsf18
Cd226
Ctla4
S1pr2
Havcr2
Sell
Lag3
Ccr5
Pdcd1
Ccr7
Tigit
Cxcr3
Ebi3
Cxcr4
Ifng
Cxcr5
Il10
Il21
Il4
Il7r
Atf6
Ahr
Ascl2
Batf
Bcl6
Bhlhe40
Cbfa2t3
Cebpa
Grhl1
Id3
Irf4
Lef1
Maf
Myc
Nfia
Nfil3
Pou2af1
Prdm1
Runx2
Tbx21
Tcf7
Tox2
Vdr
Entpd1
Itk
Nt5e

Supplementary Figure 13. **Cartoon summarizing progressive changes in the expression of key Tconv, TFH and TR1 genes from Suppl. Table 1 in the different clusters from main Fig. 8A**. Differential analysis compares each cell type to the hypothetical preceding cell state (Ag-induced TFH.1 vs Tconv, Tet+ TFH-like (TFH.1) vs Ag-induced TFH.1, Tet+ TR1-like vs Tet+ TFH-like (TFH.1), and Tet+ TR1 vs Tet+ TR1-like). T conventional markers were obtained by differential analysis relative to the other cell subsets. Data are consistent with pMHCII-NP-induced conversion of autoantigen-experienced TFH cells into transitional TR1-like and terminally differentiated TR1 cells *in vivo*.

Suppl. Fig. 14

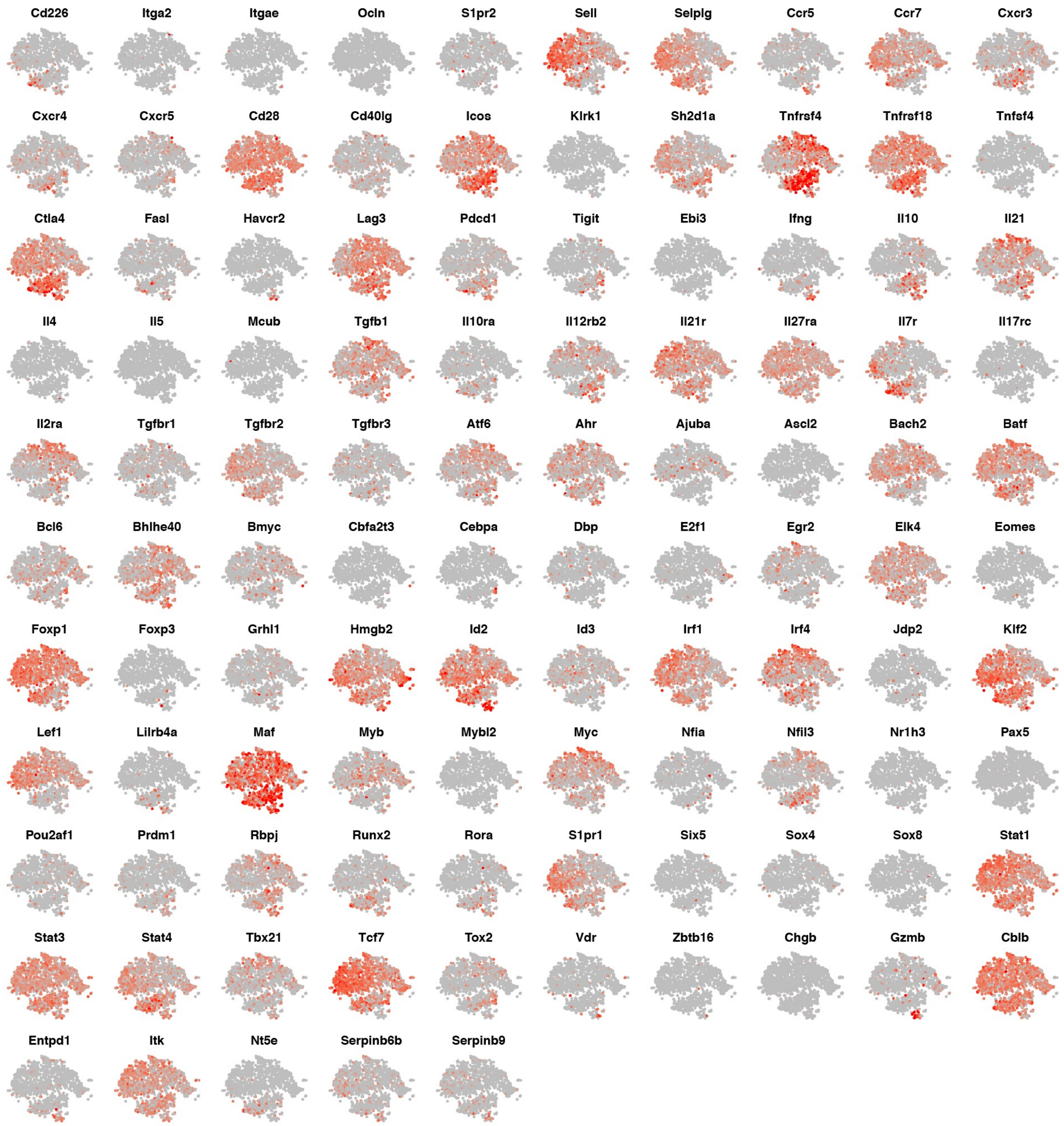
Cd226
Itga2
Itgae
Ocln
S1pr2
Sell
Selplg
Ccr5
Ccr7
Cxcr3
Cxcr4
Cxcr5
Cd28
Cd40lg
Icos
Klrk1
Sh2d1a
Tnfrsf4
Tnfrsf18
Tnfsf4
Ctla4
Fasl
Havcr2
Lag3
Pdcd1
Tigit
Ebi3
Ifng
Il10
Il21
Il4
Il5
Mcub
Tgfb1
Il10ra
Il12rb2
Il21r
Il27ra
Il7r
Il17rc
Il2ra
Tgfbr1
Tgfbr2
Tgfbr3
Atf6
Ahr
Ajuba
Ascl2
Bach2
Batf
Bcl6
Bhlhe40
Bmyc
Cbfa2t3
Cebpa
Dbp
E2f1
Egr2
Elk4
Eomes
Foxp1
Foxp3
Grhl1
Hmgb2
Id2
Id3
Irf1
Irf4
Jdp2
Klf2
Lef1
Lilrb4a
Maf
Myb
Mybl2
Myc
Nfia
Nfil3
Nr1h3
Pax5
Pou2af1
Prdm1
Rbpj
Runx2
Rora
S1pr1
Six5
Sox4
Sox8
Stat1
Stat3
Stat4
Tbx21
Tcf7
Tox2
Vdr
Zbtb16
Chgb
Gzmb
Cblb
Entpd1
Itk
Nt5e
Serpinb6b
Serpinb9

Supplementary Figure 14. Expression of 106 TR1/TFH/Treg-relevant genes in eGFP (IL-10)+ CD4+ T-cells from anti-CD3 mAb-treated NOD.*Il10-eGFP* and NOD.*Il10-eGFP*.*Cd4-Cre Bcl6$^{loxP/loxP}$ m*ice. Figure shows feature plots for the 106 TR1/TFH/Treg genes from **Supplementary Table 1**. Since gene expression in individual cells as detected by scRNAseq is less sensitive that eGFP reporter expression, a significant number of the eGFP+ cells used for scRNAseq express low levels of IL-10 mRNA and are therefore not coloured in red in the feature plots.

Suppl. Fig. 15

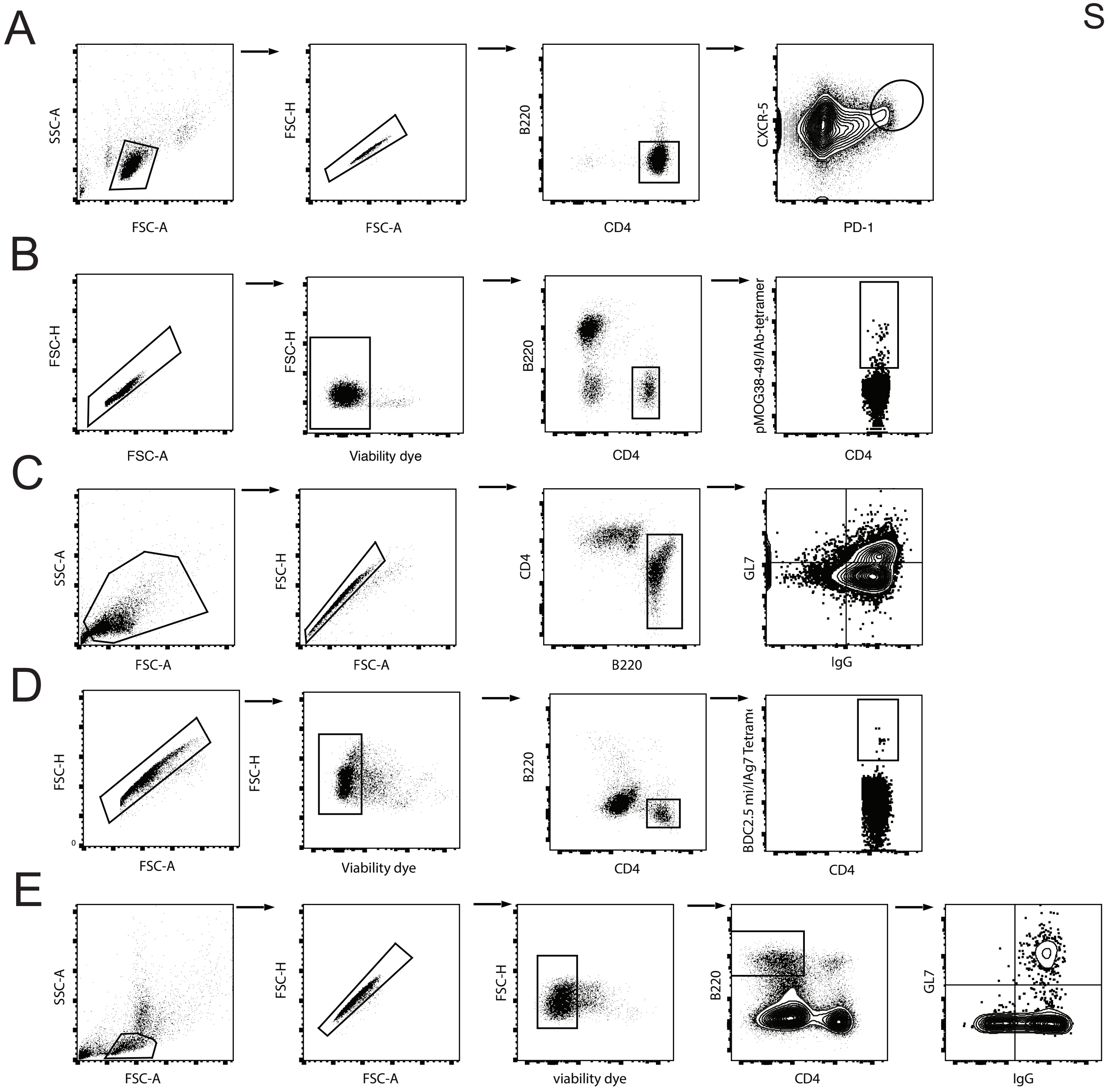
A
SSC-A
FSC-A
FSC-H
FSC-A
B220
CD4
CXCR-5
PD-1
B
FSC-H
FSC-A
FSC-H
Viability dye
B220
CD4
pMOG38-49/IAb-tetramer
CD4
C
SSC-A
FSC-A
FSC-H
FSC-A
CD4
B220
GL7
IgG
D
FSC-H
FSC-A
FSC-H
Viability dye
B220
CD4
BDC2.5 mi/IAg7 Tetram
CD4
E
SSC-A
FSC-A
FSC-H
FSC-A
FSC-H
viability dye
B220
CD4
GL7
IgG

Supplementary Figure 15. Representative flow cytometry gating profiles for main Figures 9 and 10. A, Gating profile for CXCR5^{hi}PD-1^{high} CD4+ T-cells transfused into NOD.*Scid* hosts in **Fig. 9A. B,** Gating profile for pMOG$_{38\text{-}49}$/I-A^{b} Tet+ cells in mice treated with pMOG$_{38\text{-}49}$/I-A^{b}-NPs. **C,** Gating profile for B220^{+}/GL7^{+}/IgG^{+} B cells from **Fig. 10C**. **D**, Gating profile for the BDC2.5mi/I-A^{g7} Tet+ cells of the NOD.*Scid* hosts from **Fig. 10E**. **E**, Gating profiles for the GL7^{+}sIgG^{+}, GL7^{-}sIgG^{+} and GL7^{-}sIgG^{-} cells from **Fig. 10F**.

Suppl. Fig. 16

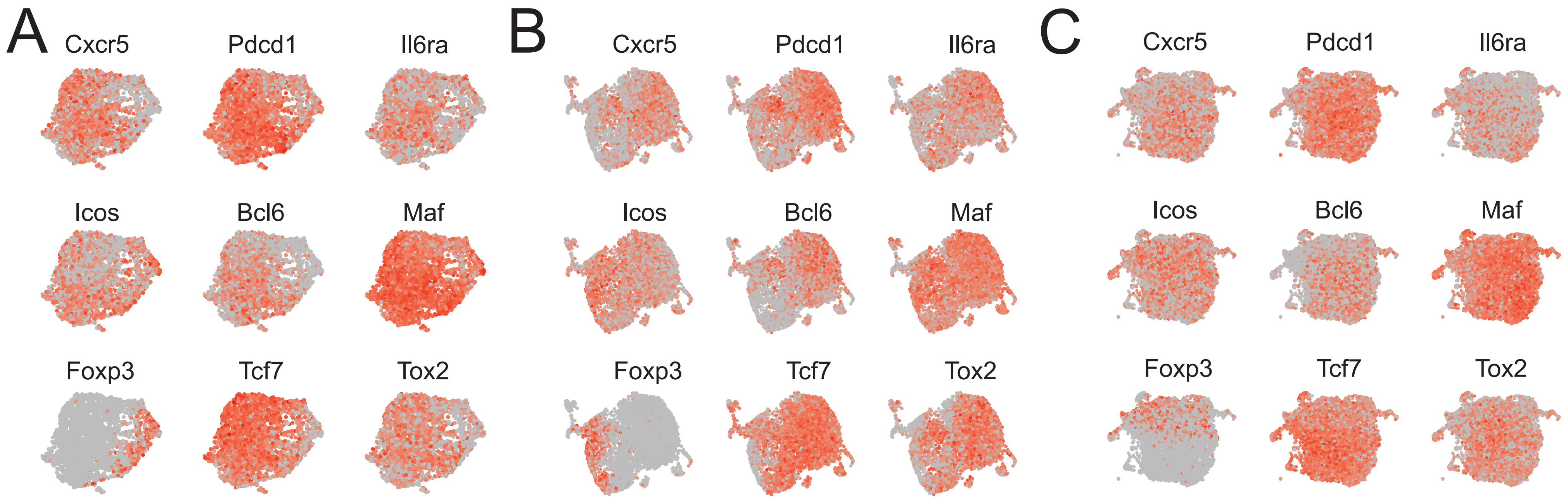
A
Cxcr5
Pdcd1
Il6ra
Icos
Bcl6
Maf
Foxp3
Tcf7
Tox2
B
Cxcr5
Pdcd1
Il6ra
Icos
Bcl6
Maf
Foxp3
Tcf7
Tox2
C
Cxcr5
Pdcd1
Il6ra
Icos
Bcl6
Maf
Foxp3
Tcf7
Tox2

Supplementary Figure 16. UMAP-based feature plots for representative TFH-associated gene transcripts (and FoxP3) corresponding to the CXCR5^{hi}PD-1^{high} CD4^{+} T-cells from NOD (A), NOD.*Cd4-Cre* (B) and NOD.*Cd4-Cre/Prdm1*$^{loxP/loxP}$ mice (C) used for the NOD.*Scid* transfer experiments from Fig. 9.

Suppl. Fig. 17

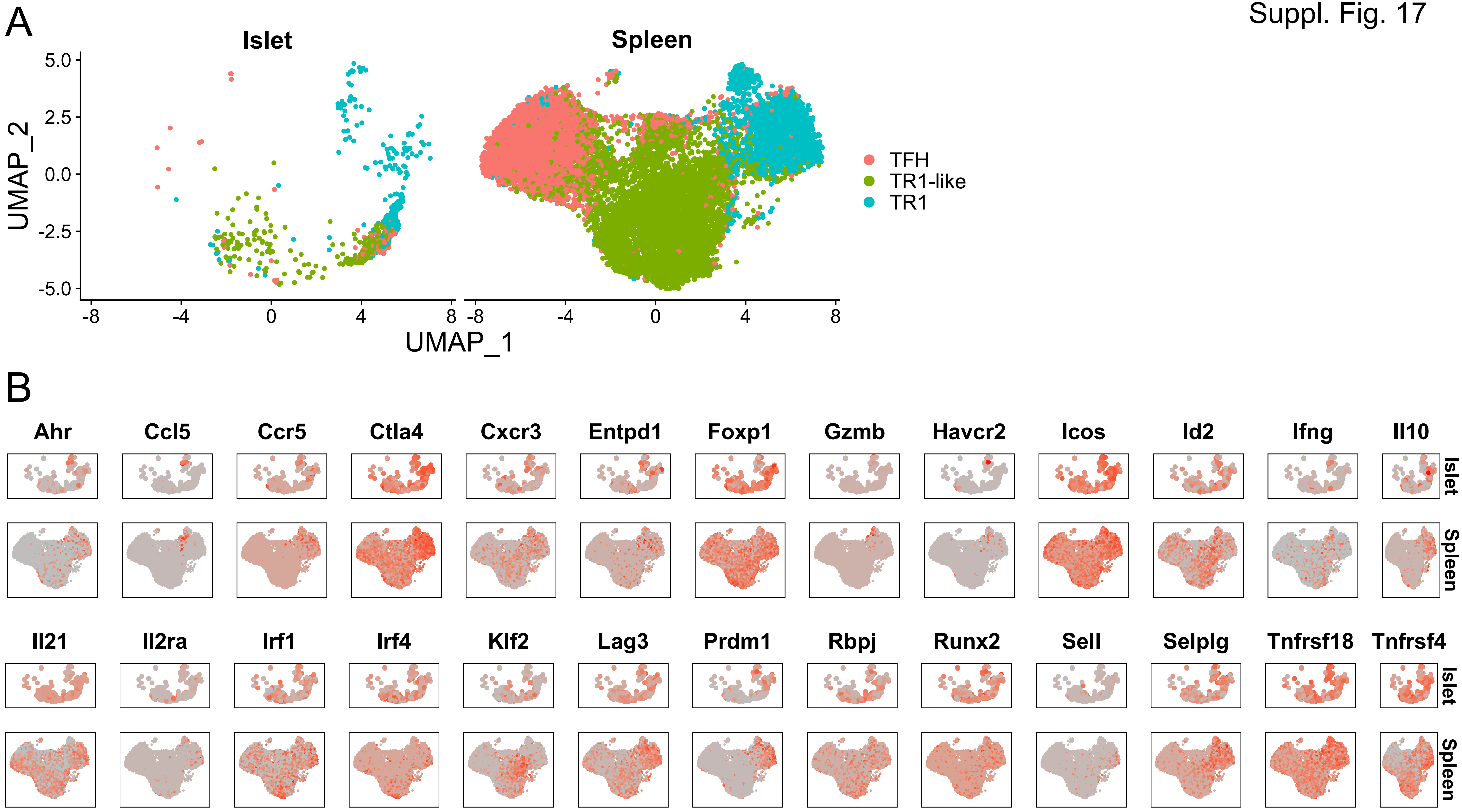
A
Islet
Spleen
UMAP_2
UMAP_1
5.0
2.5
0.0
-2.5
-5.0
-8
-4
0
4
8
TFH
TR1-like
TR1
B
Ahr
Ccl5
Ccr5
Ctla4
Cxcr3
Entpd1
Foxp1
Gzmb
Havcr2
Icos
Id2
Ifng
Il10
Il21
Il2ra
Irf1
Irf4
Klf2
Lag3
Prdm1
Rbpj
Runx2
Sell
Selplg
Tnfrsf18
Tnfrsf4
Islet
Spleen

Supplementary Figure 17. UMAP plots and UMAP-based feature plots for TR1-associated gene transcripts for tetramer^{+} CD4^{+} cells isolated from islets vs spleen of pMHCII-NP-treated NOD mice. A, UMAP plots. **B,** Comparison of the expression of genes from Suppl. Table 1 that are differentially upregulated by terminally differentiated TR1 cells vs. TR1-like and/or TFH cells (from Fig. 6C), by the tetramer^{+} cells isolated from islets vs spleen of BDC2.5mi/IA^{g7}-NP-treated NOD mice.
